# Decoding the metabolic dialogue in the tumor microenvironment: from immune suppression to precision cancer therapies

**DOI:** 10.1186/s40164-025-00689-6

**Published:** 2025-07-22

**Authors:** Ruoli Wang, Jincheng Zhuang, Qi Zhang, Wantao Wu, Xinrui Yu, Hao Zhang, Zongyi Xie

**Affiliations:** 1 Department of Neurosurgery, The Second Affiliated Hospital, Chongqing Medical University, Chongqing, China; 2 Department of Thyroid and Breast Surgery, The Second Affiliated Hospital, Chongqing Medical University, Chongqing, China

**Keywords:** Tumor microenvironment, Immune cells metabolism, Metabolic reprogramming, Immunotherapy resistance, Therapeutic targeting

## Abstract

The tumor microenvironment (TME) represents a metabolic battleground where immune cells and cancer cells vie for essential nutrients, ultimately influencing antitumor immunity and treatment outcomes. Recent advancements have shed light on how the metabolic reprogramming of immune cells, including macrophages, T cells, and DCs, determines their functional polarization, survival, and interactions within the TME. Factors such as hypoxia, acidosis, and nutrient deprivation drive immune cells toward immunosuppressive phenotypes, while metabolic interactions between tumors and stromal cells further entrench therapeutic resistance. This review synthesizes new insights into the metabolic checkpoints that regulate immune cell behavior, focusing on processes like glycolysis, oxidative phosphorylation (OXPHOS), lipid oxidation, and amino acid dependencies. We emphasize how metabolic enzymes (e.g., IDO1, ACLY, CPT1A) and metabolites (e.g., lactate, kynurenine) facilitate immune evasion, and we propose strategies to reverse these pathways. Innovations such as single-cell metabolomics, spatial profiling, and AI-driven drug discovery are transforming our understanding of metabolic heterogeneity and its clinical implications. Furthermore, we discuss cutting-edge therapeutic approaches—from dual-targeting metabolic inhibitors to biomaterial-based delivery systems—that aim to reprogram immune cell metabolism and enhance the effectiveness of immunotherapy. Despite the promise in preclinical studies, challenges persist in translating these findings to clinical applications, including biomarker validation, metabolic plasticity, and interpatient variability. By connecting mechanistic discoveries with translational applications, this review highlights the potential of immunometabolic targeting to overcome resistance and redefine precision oncology.

## Introduction

Tumors are currently one of the most important causes of human death. Past studies have shown that tumorigenesis is closely related to the metabolic processes of immune cells in tumors, and the concept of the tumor microenvironment (TME) has been proposed [1, 2]. TME contains tumor cells, non-tumor cells, and other tumor-related small molecule compounds, and immune cells are among the most critical components of the TME. Immune cells significantly influence the TME through a series of their roles, including metabolic reprogramming and signaling factors, which affect the processes of tumorigenesis and development [3]. The immune system is an essential component of the human body, which protects against foreign pathogens and removes cells that grow abnormally. The most important part of the immune system is the immune cells, which are the main body of the immune system that performs its function. The immune cells are also used as a target for disease treatment [4]. Metabolism is the study of how cells utilize nutrients, environment, and the products of cellular metabolism profoundly influence the surrounding tissue environment [5]. In recent years, the mechanisms of metabolism and disease development have been continuously discovered, and the diseases of various systems throughout the human body have a close relationship with metabolism [6–8]. Past studies have confirmed that immune cells, through their metabolism, can influence the nutrient composition of the TME and the pH of the environment to cause corresponding pathological changes [9].

Recent research highlights an urgent need for innovative approaches to tumor treatment, making the exploration of metabolism in this context a promising avenue for therapeutic development [10]. Additionally, considering the crucial role of immune cells within tumor cells and the TME, investigating the metabolism of these immune cells and integrating metabolic strategies with immunotherapy and targeted therapy presents a viable path forward [11]. Quite several recent studies have also proved the feasibility of this direction: including the existing research on nanomaterials, which can be used for transferring the mitochondria between the immune cells and the tumor cells by making the metabolic shifts in therapy [12]. A research team found that azithromycin administered after hematopoietic stem cell transplantation for hematologic malignancies inhibits glucose metabolism as well as mitochondrial metabolism leading to immunosuppressive effects of T cells on tumor cells resulting in recurrence [13]. There are also studies showing that the S100A4 gene promotes macrophage differentiation towards a tumor phenotype and suggesting that this gene is a possible target for tumor therapy [14]. In conclusion, recognizing the significance of immune cell metabolism in tumor development and treatment is crucial. A thorough understanding of the metabolic mechanisms governing immune cells is vital for either inhibiting or promoting tumor growth.

**Fig. 1:**
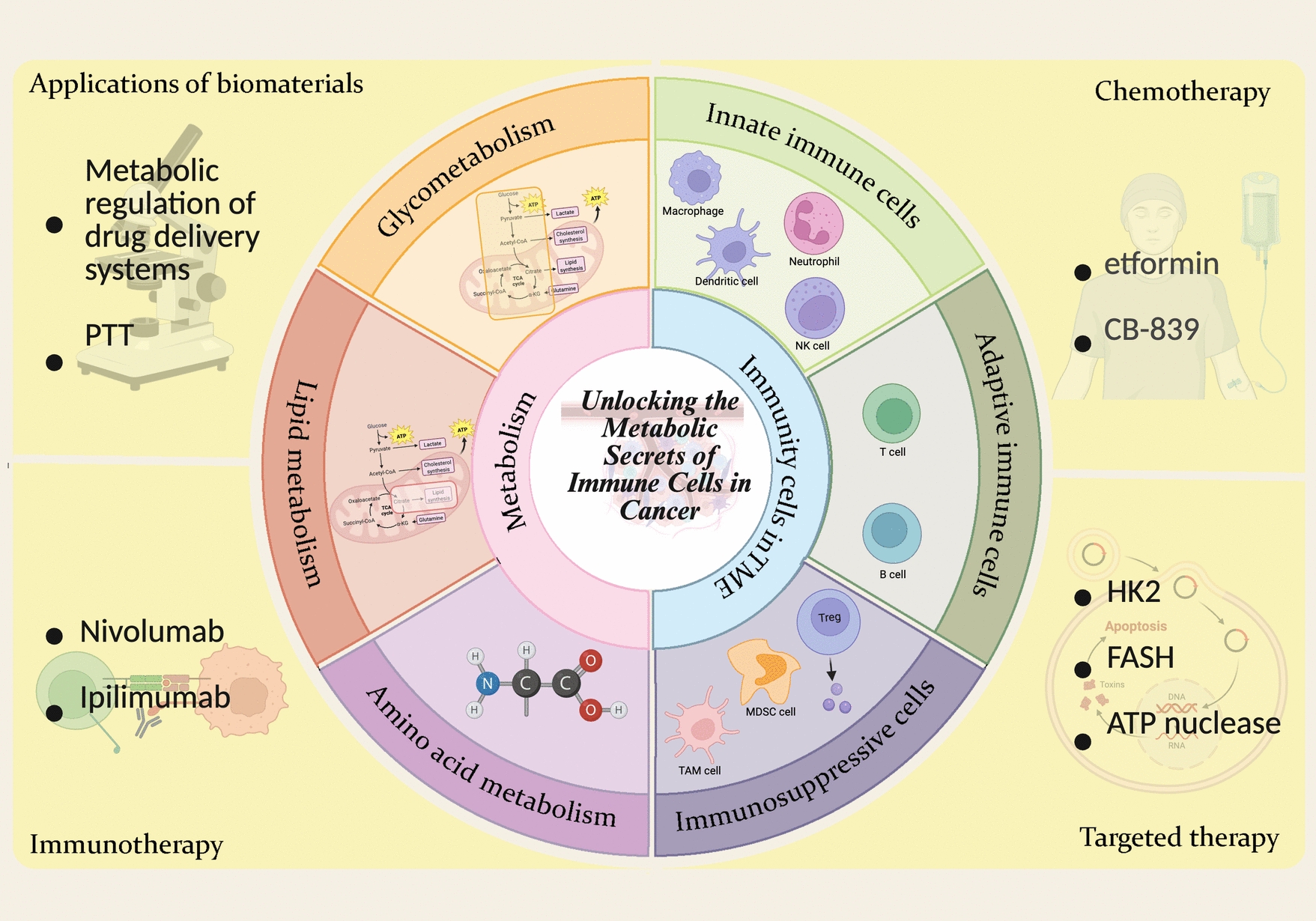
The metabolism-related mechanisms of immune cells in tumors and the existing treatment strategies targeting immune cell metabolism. Created with BioRender.com

This review delves into the metabolism of tumor cells as well as various immune cells within the TME. We will explore how immune cells influence tumor growth and progression through their metabolic activities, examine current findings on the application of immune cell metabolism in tumor therapies, and outline potential future directions for utilizing immune cell metabolism in treatment strategies (Fig. 1).

## TME

### Introduction to the TME

**Fig. 2:**
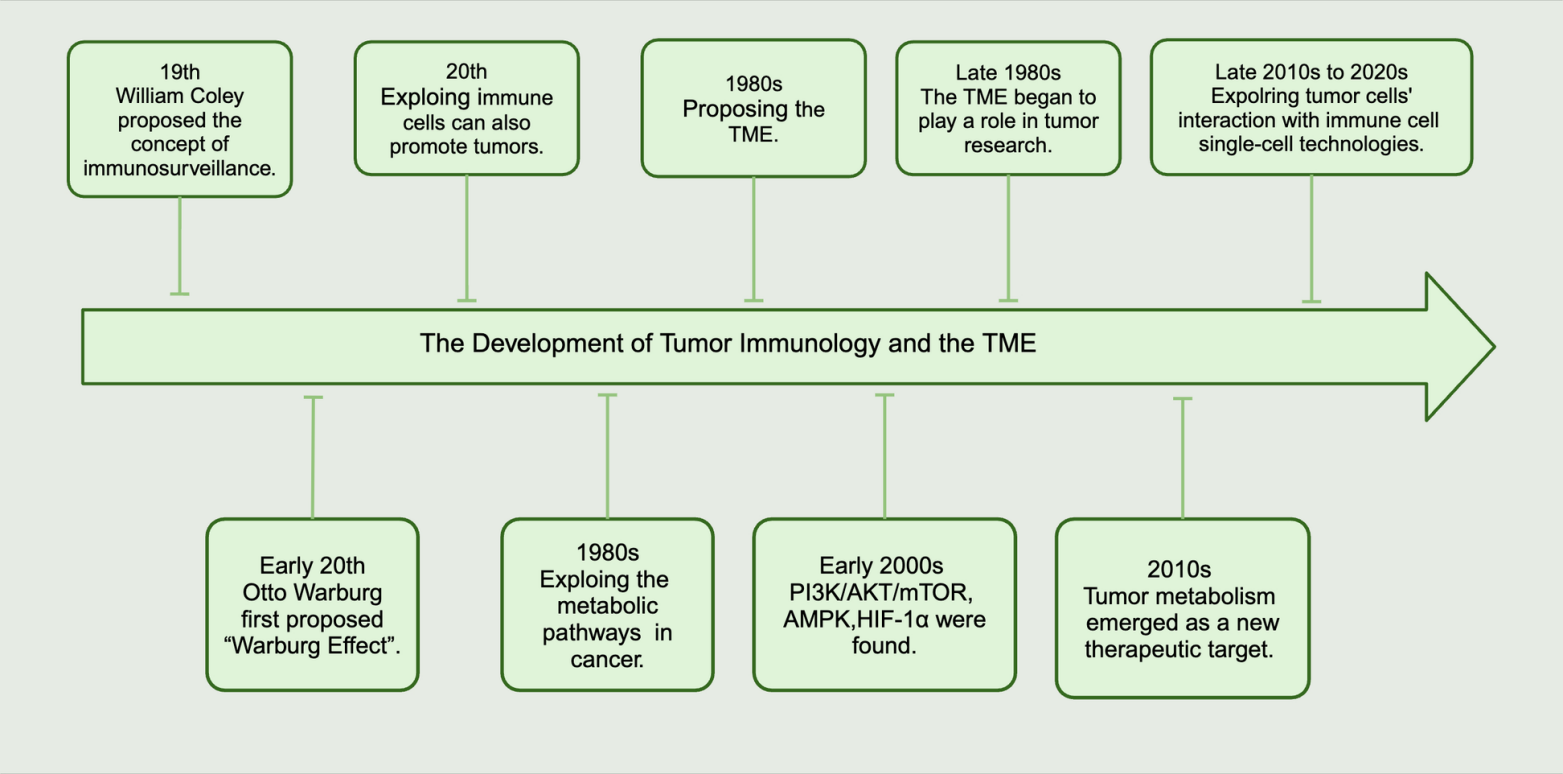
A timeline for the development of tumor immunology and the TME. The upper section primarily discusses recent developments in cancer research, whereas the lower section highlights advancements in metabolism. Created with BioRender.com

Over the past decade, many breakthroughs have been made in tumor research (Fig. 2), and our understanding of tumors as a disease has changed dramatically [15], and the concept of the TME is one of these achievements. From the nineteenth century to the early twentieth century, renowned scientist William Coley proposed the concept of “immunosurveillance,” suggesting that the immune system can recognize and eliminate cancer cells [16]. This combined immunology and oncology research, and from then on, immunity has been recognized as a central theme in tumor research [17]. As tumor immunology progressed, scientists realized that tumor cells could escape immune surveillance through various mechanisms, especially with regulatory T cells (Treg), myeloid-derived suppressor cells (MDSC), and macrophages, which suppress anti-tumor immune responses by secreting cytokines (such as IL-10 and TGF-β). These findings showed that not all immune cells in the immune system inhibit tumors [18]. At the same time, German biochemist Otto Warburg first proposed that tumor cells, even with sufficient oxygen, prefer to generate energy via glycolysis rather than OXPHOS, a phenomenon now known as the “Warburg Effect”. This discovery highlighted the metabolic differences between cancer cells and normal cells and laid the foundation for subsequent research on tumor metabolism [19]. In the 1980 s, with the development of biochemical and molecular biology techniques, researchers explored the metabolic pathways involved in cancer in depth, such as glycolysis, fatty acid synthesis (FAS), and amino acid metabolism. Scientists began to realize that cancer development was not solely a result of genetic mutations, but that the microenvironment also played a critical role in tumor progression [20]. In the early 2000 s, advancements in molecular biology technologies (such as genomics, transcriptomics, and proteomics) allowed for a deeper understanding of the molecular mechanisms of tumor metabolism. Key metabolic regulators, such as PI3K/AKT/mTOR and AMPK, were found to regulate the metabolic reprogramming in tumor cells [21]. The discovery of HIF-1α helped researchers understand how hypoxic conditions promote tumor metabolic alterations, influencing tumor growth and metastasis [22]. In the 2010 s, tumor metabolism emerged as a new therapeutic target. Researchers found that cancer cells have specific metabolic pathways, such as glucose, glutamine, and fatty acids, which could be potential targets for treatment. Also, researchers have focused on remodeling the TME to enhance the efficacy of immunotherapy, primarily by activating or inhibiting immune cell metabolism. [23, 24] By the late 2010 s to 2020 s, with the advent of single-cell technologies, researchers began analyzing the metabolism of individual cells within the TME, revealing metabolic heterogeneity in tumor cells and their interaction with immune cells and cancer stem cells [25].

The TME is a large, complex and dynamic system composed of tumor cells and other non-cancerous components, which has many essential relationships with cancer cell proliferation, neovascularization, immune surveillance, and immune escape, and has brought about a dramatic change in our understanding of tumor refractoriness, which was often caused by metastasis and recurrence of advanced tumors [26]. The TME has made us realize that tumor cells can form their own ecosystems and put themselves at the top of these ecosystems, which can make factors in the environment work for them, causing tumor infiltration and progression [27]. The TME triggers tumor cells to survive for a long time in many ways [28–30]. One of the essential things is that the TME will show heterogeneity in many cases, that is, with the continuous growth of tumor cells, they will secrete many cytokines and chemokines, making immune cells in the TME undergo reprogramming continuously. Reprogramming is mainly caused by metabolism, including hypoxic conditions, which will promote the reprogramming of cells in the TME and cause metabolic changes, resulting in tumor metastasis. Hypoxic environments will generate lactic acid, resulting in the TME becoming an acidic environment, which is conducive to the growth of tumors, because the acidic environment can damage the double strands of DNA and delay the time of its repair [31]. On a broader level, acidic conditions can also induce chromosomal damage [32]. Moreover, histone modification, which leads to chromosome remodeling, is one of them [33]. Due to the inherent uncertainty associated with the changes resulting from reprogramming, research on the TME can be divided into two main areas [34]. First, considering that immune cells are a vital component of the TME, what are the mechanisms of interaction between immune cells and tumor cells? Second, in what ways do the immune cells within the microenvironment influence tumor cells through metabolic pathways? Additionally, what new strategies can be developed to inhibit tumor growth based on these insights?

### Metabolism of tumor cells in the TME

#### Glucose metabolism

Tumor cells, as the product of the mutation of normal cells, also take in relevant nutrients from the external environment to maintain their growth as normal cells do. The primary metabolic nutrients of tumor cells are glucose and amino acids, which are converted into cellular components after metabolism [35]. The most crucial metabolism of tumor cells is glucose metabolism, and the aerobic glucose metabolism and glycolysis of tumor cells are more potent than those of other cells. At the same time, tumor cells will preferentially carry out glycolysis over aerobic glucose metabolism. Producing pyruvate will be directly converted into lactic acid, further inhibiting aerobic respiration. This phenomenon is known as the Warburg effect and is the most representative of the glucose metabolism of tumor cells [36]. Some recent studies on the Warburg effect have shown that NADH regeneration from GAPDH by lactate dehydrogenase-catalyzed NAD + promotes glycolysis because the increased ratio of NADH to NAD + inhibits GAPDH [37], and p53 has been found to inhibit the onset of this process in a breast cancer model [38]. Meanwhile, some studies have found that cadmium exposure is also positively correlated with the Warburg effect in tumor cells, but the specific mechanism needs to be further studied and discovered [39]. Tumor cell glucose metabolism is regulated by many factors: Glucose transporter protein 1 (GLUT1), a membrane protein, plays a key role in the glucose transport. Inhibition of GLUT1-mediated glucose transport can effectively block tumor glycolysis. Additionally, suppression of Zinc Finger DHHC-Type Palmitoyltransferase 9 (DHHC9) can lead to the failure of GLUT1 to localize in the plasma membrane and thus reduce the level of glucose metabolism in tumor cells [40]. Moreover, a correlation has been observed between GLUT1 and the retinoblastoma inhibitory factor RB1. Inhibition of GLUT1 expression can cause the expression of RB1 to inhibit tumor growth [41]. Transcription factor chimeric forkhead box M1- proteolysis targeting chimera (FOXM1-PROTAC) can also inhibit the expression of GLUT1, so the tumor cells lose the ability to metabolize glucose, achieving the purpose of the inhibition [42].

The expression of tumor cells'genes can promote the tumor’s glucose metabolism. The expression of METTL5 in hepatocellular carcinoma causes enhancement of glucose metabolism and then promotes the growth of tumors, which is achieved by promoting the stability of c-Myc, and further by promoting the translation of USP5, which in turn regulates the ubiquitylation of c-Myc [43, 44], in breast cancer, the high level of tumor cell-derived of extracellular vesicle-encapsulated miR-122 targeting PKMin-β cells can inhibit the gluconeogenesis process leading to glucose metabolism disorders in the human body, thus allowing tumor cells to obtain more glucose resources in competition [45]. Under hypoxic conditions, HIF-1α can cause changes in Smad's structural domains, promoting the function of TGF-β and the glycolysis of tumor cells. Glycolysis of tumor cells [46]. In addition to promoting glycolysis, some genes in tumor cells can convert glycolysis into lipid metabolism to meet their own metabolic needs. Sirtuin1 (SIRT1) plays a central role in regulating glycolipid metabolism in tumor cells in colorectal cancer. It promotes lipid metabolism while inhibiting glycolysis by deacetylating β-catenin, facilitating translocation from the nucleus to the cytoplasm. This mechanism is closely associated with disease prognosis [47].

#### Amino acid metabolism

The amino acid most predominantly utilized by tumor cells is glutamine, whose metabolism is second only to glucose metabolism in importance. The glutamine metabolism requires glutaminase (GLS) to convert it to glutamate [48], which promotes the growth of tumor cells through the reprogramming of glutamine metabolism that occurs in tumor cells [49]. For example, transporter proteins such as SLC1A1 and SLC7A11 encourage the development of tumors by influencing the process of glutamine transport to the mitochondria, illustrating the importance of this metabolic process [50, 51]. However, we are unfamiliar with the specific process by which glutamine promotes tumor cell growth. SUCLA2-coupled succinylation regulation of GLS plays a key role in the regulation of glutamine, which enhances the activity of GLS, leading to the ability of tumor cells to counteract oxidative stress and promote their growth [52], and is also associated with the down-regulation of glutamine transaminase 2 (GOT2) in hepatocellular carcinoma (HCC) cells. Among them, we found that GOT2 silencing enhances glutamine catabolism, nucleotide synthesis, and glutathione synthesis by reprogramming glutamine metabolism to support the cellular antioxidant system and its activation of the PI3K/AKT/mTOR pathway to promote HCC progression. [53]

Besides glutamine, other amino acids are also metabolized in tumor cells: Serine and glycine are essential sources of one-carbon metabolism, and the metabolism of serine is mainly due to the interconversion of serine and glycine [54], which provides one-carbon units for the synthesis of DNA and RNA, key components required for cellular proliferation. This synthesis process is also closely linked to the mitochondria in the tumor cells [55]. Additionally, it contributes to the synthesis of glutathione (glycine, cysteine, and glutamate), which helps maintain cellular redox balance by eliminating reactive oxygen species, thereby promoting tumor cell survival [56]. Proline is the only secondary amino acid contained in the body, which is inextricably linked to collagen synthesis, and collagen is also involved in tumorigenesis and metastasis [57]. Therefore, proline metabolism is also a part of amino acid metabolism that cannot be ignored by the tumor cells. Arg (Arg) is an essential amino acid that plays a role mainly in the urea cycle [58], and Arg methylation affects the growth and development of tumor cells by regulating the synthesis of polyamines, nitric oxide, and immune complexes [59]. Arg and its methylation products also act as a signal that modulates various metabolic pathways, including glucose metabolism [60]. Many studies have confirmed its association with tumorigenesis [61], highlighting its critical role in amino acid metabolism within tumor cells.

#### Lipid metabolism

**Fig. 3:**
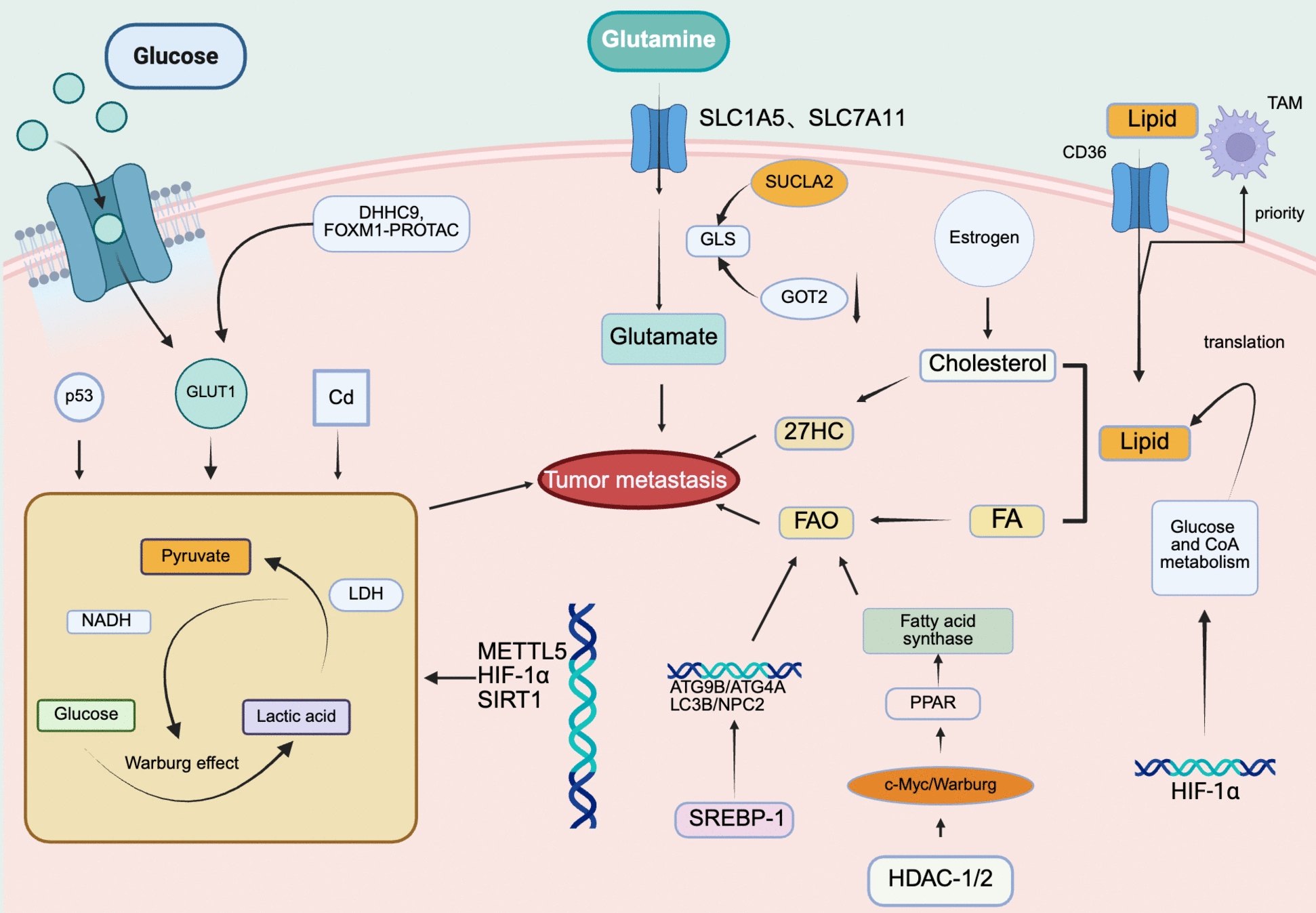
The main metabolism of tumor cells promotes their growth. Cells and their three most essential metabolic pathways—glucose metabolism, lipid metabolism, and amino acid metabolism—play a crucial role in cellular function. Among these pathways, the Warburg effect stands out as a hallmark of tumor glucose metabolism. The mechanisms discussed in the article are illustrated on the left side of the figure, along with recent related studies. In tumor cells, amino acid metabolism largely centers around glutamine, which is primarily regulated by transporter proteins. Additionally, lipid metabolism in tumor cells encompasses pathways associated with cholesterol and fatty acids, which are vital for supporting tumor growth and metastasis. Created with BioRender.com. *DHHC9* Asp—His—His—Cys box—containing protein, *9FOXM1—PROTAC* Forkhead box M1—Proteolysis—Targeting Chimera, *p53* Tumor protein p53, *GLUT1* Glucose Transporter 1, *Cd* Cadmium, *SLC1A5* Solute Carrier Family 1 Member 5, *SLC7A11* Solute Carrier Family 7 Member 11, *SUCLA2* Succinate—CoA Ligase, *GOT2* Glutamic—Oxaloacetic Transaminase 2, *CD36* CD36 Molecule, *27HC* 27—Hydroxycholesterol; *PPAR* Peroxisome Proliferator—Activated Receptor, *HDAC—1/2* Histone Deacetylase ½, *SREBP-1* Sterol Regulatory Element Binding Protein 1, *NPC2* Niemann—Pick Disease Type C2 Protein, *METTL5* Methyltransferase Like 5, *SIRT1* Sirtuin 1

Lipid metabolism is also an essential part of tumor cell metabolism. Lipid acquisition and oxidation are inextricably linked to tumorigenesis and metastasis. Tumor cells need the CD36 transporter protein to obtain lipids through uptake. Various studies have confirmed the association between CD36 expression and malignant tumors [62]. After uptake of fatty acids, CD36 preferentially transports them to metastasis-associated macrophages (MAMs) in the TME to enhance their metabolic activity and thus play a tumor-promoting role [63]. In breast cancer specimens, cholesterol uptake can be converted to 27-hydroxycholesterol (27HC) via CYP27A1, which can lead to tumor metastasis, and tumor growth is also reduced after using CYP27A1 inhibitors [64]. In hepatocellular carcinoma, estrogens have been found to up-regulate Lecithin-Cholesterol Acyltransferase (LCAT), which promotes high-density lipoprotein (HDL) cholesterol uptake via the low-density lipoprotein receptor (LDLR) and SCARB1 pathways. This process impairs sterol regulatory element-binding protein F (SREBPF), which is a key component in the metabolic pathway of the tumor. This process impairs SREBP2 maturation, inhibiting cholesterol uptake and synthesis to attenuate hepatocellular carcinoma metastasis [65]. Tumor cells can synthesize lipids by themselves. In recent years, studies have shown that fatty acid synthase is overexpressed in tumors, indicating the role of fatty acids in tumor development [66]. This process is often associated with hypoxia in tumor cells, and HIF-1α induces the production of fatty acid synthase in cancer cells, thus shifting the metabolism from glucose metabolism and acetyl coenzyme A to lipid metabolism [67]. Also, lipolysis promotes tumor growth, and the association between lipolysis and cancer development has been confirmed by the increase in lipoprotein lipase (LPL) found in several studies [68], which also allows us to assess the prognosis of tumor therapy [69]. Fatty acid metabolism is highly active in various tumors, according to recent studies [70]. Fatty acid metabolism requires the activation of PPAR, a regulator of fatty acid metabolism. In glioblastoma cells, histone deacetylase 1/2 (HDAC-1/2) can contribute to the Warburg effect through the c-Myc pathway. Inhibition of this pathway results in the activation of peroxisome proliferator-activated receptor D (PPARD), resulting in the dependence of tumor metabolism on fatty acid oxidation (FAO). These findings indicate that PPARD may be an essential receptor for fatty acid metabolism in tumor cells [71]. Cholesterol, as a constituent of cell membranes, has a high activity in tumor cells, and to maintain cholesterol content in glioblastomas, the up-regulation of critical Autophagy genes, including ATG9B, ATG4A, LC3B, and NPC2, cause hydrolysis of cholesteryl esters in tumor cells to maintain cholesterol for tumor cell metabolism [72] (Fig. 3 and Table 1).

**Table 1:** Tumor metabolism-related targets

Target	Metabolic types	Mechanism	Refs.
GLUT1	Glucose metabolism	Playing a key role in glucose transport and blocking GLUT1 inhibits tumor glycolysis	[40]
METTL5	Glucose metabolism	Promoting tumor growth; Regulating the ubiquitination of c-Myc and its stability	[43, 44]
miR-122	Glucose metabolism	Inhibiting the process of glucose metabolism	[45]
HIF-1α	Glucose metabolism	Causing changes in the structural domain of Smad	[46]
SIRT1	Glucose metabolism	Deacetylating β-actinin	[47]
SLC7A11	Amino acid metabolism	Enhancing glutamine metabolism by affecting the process of glutamine transport to mitochondria	[50, 51]
SUCLA2	Amino acid metabolism	Enhancing GLS activity	[52]
GOT2	Amino acid metabolism	Promoting tumor growth	[53]
CD36	Lipid metabolism	Promoting tumor cell growth	[63]
SREBP-1	Lipid metabolism	Promoting the uptake of high-density lipoprotein cholesterol and the metastasis of tumor cells	[72]
CYP27A1	Lipid metabolism	Converting cholesterol to 27HC, which triggers the metastasis of tumors	[64]
LCAT	Lipid metabolism	Promoting the uptake of high-density lipoprotein cholesterol and the metastasis of tumor cells	[66]
HDAC-1	Lipid metabolism	PPARD activation leads to tumor metabolism dependent on FAO	[71]
HDAC-2	Lipid metabolism	PPARD activation leads to tumor metabolism dependent on FAO	[71]

### Immune cell-metabolism linkages in the TME

Many immune cells are in the TME, including fibroblasts, macrophages, neutrophils, dendritic cells (DCs), NK cells, etc [73]. These immune cells and their secreted cytokines and metabolites constitute and maintain the ecology of the TME. In recent years, there has been sustained interest in understanding the role of immune metabolism within the TME. More and more studies have shown the critical role of immune metabolism in cancer escape, invasion, and metastasis. The metabolism of immune cells includes glycolysis, lipid metabolism, and amino acid metabolism. On the one hand, metabolites produced by these metabolisms can affect the growth of tumor cells. For example, by studying the fate of lactic acid by 13 C isotope labeling, we found that lactic acid produced by immune cells’ glycolysis in the TME is converted to lipids, which can promote the growth of tumor cells [74]. Acetyl coenzyme A is an essential metabolite that is the hub of various metabolisms in immune cells, the abundance and activity of enzymes that regulate acetyl coenzyme A are altered by receiving influences from the TME, thus promoting tumor growth [75]. Alterations in these enzymes primarily lead to changes in the acetyl-CoA metabolic pathway, including the mevalonate pathway, DNL, and protein acetylation. Numerous studies and clinical trials have shown that tumor progression relies heavily on the mevalonate pathway. The anti-cancer effects observed through targeting acetyl-CoA may act via this pathway, although definitive evidence is still lacking. DNL enhances the production of acetyl-CoA in tumor lipid metabolism, and the downstream utilization of acetyl-CoA, particularly through protein acetylation, has been shown to promote tumor cell growth. This process has been supported by substantial evidence. Overall, these three pathways are closely associated with tumor growth, and future studies may focus on these targets to develop more effective therapeutic agents [76]. Conversely, these metabolites can also promote tumor growth by stimulating immune cells to produce immune factors. Recent studies show that tumor cell metabolites can promote tumorigenesis by stimulating T-cells to produce MOESIN and TGF-β [77]. Additionally, NAD regulates tumor immunity by controlling CD8 + T-cells and can induce PD-L1 expression in tumor cells [78]. These examples illustrate that immune cells'metabolic mechanisms can significantly impact tumor growth and development. Below, we will provide a detailed description of the tumor-associated metabolic mechanisms.

## Innate immune cells

### Macrophages

#### Introduction to macrophages

Macrophages are a key component of innate immune cells in the TME, exhibiting heterogeneous phenotypes depending on the growth and developmental microenvironment [79]. These macrophages are essential in normal states, pregnancy, metabolic diseases, obesity, and autoimmune diseases [80]. Macrophages in tumor tissues are referred to as tumor-associated macrophages (TAMs), which play critical roles in tumor development and metastasis. TAMs are functionally classified into two subtypes, M1 and M2, whereas M1-type macrophages mainly play anti-tumor roles by killing tumor cells through cytotoxicity (ADCC) [81–84]. M2 macrophages are primarily involved in the metastasis of tumor cells and suppress T-cell function in tumor immunity, thereby promoting tumor growth. Given the importance of TAMs in tumors, nowadays, it has been used as an essential target for tumor therapy, by inhibiting or promoting the TAMs function, which can achieve a perfect effect on tumor inhibition [85].

#### Antitumor metabolic processes among macrophages

Anti-tumor macrophages are mainly M1-type macrophages, and the metabolism of M1 cells is usually strongly associated with tumors. Although M2-type macrophages are primarily involved in tumor progression and metastasis, recent studies show that M2-type macrophages are closely associated with tissue repair. Perhaps they also have a role in the anti-tumor process [86]. Among them, high glucose metabolism is a key metabolic process that kills tumor cells. This process is achieved through metabolic reprogramming that promotes M1 polarization, rapidly provides energy, generates ROS and NO (via iNOS), and enhances antigen presentation to activate T cells [87]. In the initial stage of tumorigenesis, macrophages mainly use glycolysis to obtain energy. With the gradual depletion of oxygen, OXPHOS eventually becomes the central metabolic pathway [88], which ultimately kills tumor cells through the coupling of glycolysis and OXPHOS as well as the production of ROS [89]. HIF-1α is a key factor in M1 macrophage glycolysis, and glycolysis can induce its overexpression. HIF-1α can inhibit glutamine synthetase (GS), which leads to the impaired motility of cancer cells, thus achieving the effect of tumor suppression [90].

The role of lipid metabolism in macrophage-mediated tumor cell growth remains controversial. Here we will discuss the lipid metabolism of macrophages related to anti-tumor. In previous studies, macrophages produce high levels of IFN-β during lipid metabolism, which causes the recruitment of natural killer cells to exert anti-tumor effects. Macrophages regulate anti-tumor immune responses through phospholipid metabolism. Phospholipid metabolism determines macrophage function and influences their polarization status in the TME. M1-type macrophages typically promote inflammatory responses and are dependent on FAO, whereas M2-type macrophages promote immunosuppression through phospholipid metabolism. PI3K pathway and prostaglandin E2 (PGE2) synthesis play essential roles in the antitumor effects of macrophages, regulating their polarization toward antitumor [91]. Antitumor activity of macrophage arachidonic acid metabolism has also been found in a model of lung cancer [92], Arachidonic acid is converted to a range of biologically active lipids such as prostaglandins, leukotrienes, and epinephrine via the lipoxygenase (LOX) and cyclooxygenase (COX) pathways [93]. These molecules are essential in macrophage polarization, affecting the tumor immune response. Arachidonic acid is converted to PGE2, which induces macrophage polarization toward the M2 type, suppressing the immune response and promoting tumor immune escape [94]. Conversely, Leukotrienes are synthesized through the LOX pathway and can regulate macrophage migration, proliferation, and immune response to tumor cells [95].

Amino acid metabolism in macrophages is mainly related to macrophage polarization. Among these, glutamine metabolism also plays a crucial role in macrophage activation [96]. It generates glutamate to support the tricarboxylic acid cycle (TCA), which promotes metabolic switching in macrophages to inhibit tumor progression [97]. Arg is converted to urea and ornithine in M1 macrophages, which are used to maintain their polarization and thus exert anti-tumor effects [98].

#### Tumor-promoting metabolic processes among macrophages

Tumor-promoting macrophages are predominantly M2 macrophages, and hypoxia in the TME promotes the polarization of macrophages to the M2 phenotype, thereby causing them to promote the process of immune escape of tumor cells [99, 100].

Regarding glucose metabolism, M2 macrophages metabolize mainly through aerobic glycolysis, producing metabolites such as lactate to form an acidic tumor microenvironment. In an environment of high aerobic glycolysis, hexokinase 2 (HK2) dissociates from mitochondria and its subsequent binding and phosphorylation of IκBα at T291, leading to increased expression of PD-L1 [101]. Additionally, the transcription factor Zeb1 promotes glycolytic activity and stimulates the PKA/CREB signaling pathway, thereby inducing the polarization of macrophages towards the immunosuppressive M2 macrophage phenotype [102]. Meanwhile, metabolites such as lactate can promote the reprogramming of macrophages in the TME and change them to the M2 phenotype, promoting immune escape [103]. In the hypoxic environment of the TME, the HIF-1α regulates the transcription of macrophage glycolysis-associated enzymes, and promotes the enhancement of glycolysis by enhancing the Toll-like receptor/nuclear factor-kB (NF-kB) and the mammalian target of PI3K/AKT/mTOR [104]. Later, as glucose is depleted in the TME, M2 macrophages utilize OXPHOS primarily for metabolism [105]. M2 macrophages maintain their metabolic stability through OXPHOS, which supports the TCA and provides ATP [88]. This metabolic profile facilitates the production of immunosuppressive factors such as IL-10 and TGF-β, inhibiting T cell activity [106]. In addition, these factors can promote the proliferation, migration, and metastasis of tumor cells and help the tumor establish an immune escape mechanism, so OXPHOS is an essential metabolism for macrophages that promotes tumor growth.

The tumor-promoting metabolism of M2 macrophages is mainly lipid metabolism, through which ATP is generated and involved in tumor escape. Fatty acid metabolism promotes metabolic reprogramming and phenotypic changes in macrophages [107], which have been shown to ingest fatty acids through high expression of the scavenger receptor CD36. Rather than relying on glycolysis, they metabolize the accumulated lipids. The ingested fatty acids are then catabolized for OXPHOS by lysosomal acid lipase (LAL) [108], thereby promoting the metastatic progression of tumors [109]. In addition to providing energy, fatty acid metabolism in macrophages can promote immune escape by upregulating tumor cell metabolism through regulatory factors, including those containing ABHD5, monoglyceride lipase (MGLL), and acyl-coenzyme A dehydrogenase medium chain (ACADM) [86]. At the same time, the corresponding pathways can promote lipid accumulation and thus activate lipid metabolism. Carnitine palmitoyltransferase-1A (CPT1A), a key enzyme in the transport of fatty acid intermediates, can be activated by lipid accumulation and therefore play a role in causing immune escape [110]. Also, free cellular glycerol is broken down by MGLL, which is often absent from M2 macrophages, leading to lipid accumulation [111]. Besides lipid accumulation, receptor-interacting protein kinase 3 (RIPK3) is down-regulated in the TME, reducing cystatin 1-mediated ROS production. This decrease subsequently activates proliferator-activated receptors 10 (PPARs), contributing to FAO-induced M2 polarization of macrophages [112].

Cholesterol is a metabolite of lipid uptake by macrophages and a raw material for synthesizing related anti-tumor factors. It has been found that in the TME, M2 macrophages show altered cholesterol metabolism that influences their function. However, the cholesterol content is relatively high, and cholesterol efflux is also high, which leads to a decrease in the synthesis of related anti-tumor factors [113]. Cholesterol efflux mediated by cholesterol transporter ATP-binding cassette A1/G1 (ABCA1/G1) promotes IL4-mediated inhibition of IFN-γ, leading to the formation of M2 macrophage phenotype that enhances the pro-tumorigenic effects [114], and the ABCA1/G1-mediated translocation of intracellular cholesterol to the extracellular compartment, which is conducive to the maintenance of the M2 pro-tumorigenic phenotype [88].

Amino acid metabolism also plays a pivotal role in the metabolism of M2 macrophages: glutamine metabolism promotes macrophage reprogramming and maintains macrophage polarization to the M2 phenotype [115]. Inhibition of GS synthesis by inhibiting its synthesis predisposes macrophages to the M1 phenotype, a process that was found to be related to the increased capacity of GS-suppressed macrophages to exhibit increased recruitment of T cells and decreased T cell inhibition. This process was found to be related to the fact that GS-inhibited macrophages showed an increased ability to recruit T cells, a weakened inhibitory effect on T cells, and a reduction in cancer cell motility [90]. α-Ketoglutaric acid (αKG) produced by glutamine catabolism promotes the M2 polarization of macrophages, and one important point is that metabolites produced by catabolism are involved in the oxidation of fatty acids, which supports the progression of tumors [116], in addition to promoting the polarization, studies have indicated that the TME is generally unfavorable for the maintenance or induction of M1 phenotype. Moreover, macrophages in the TME have been found to synthesize excess glutamine, which is transported to the extracellular compartment through transporter proteins such as SLC7A11 and used to support tumor cell growth [117].

Arg is another amino acid primarily utilized in the metabolism of M2 macrophages, which can up-regulate the transcription of Arginase 1 (ARG-1), which catabolizes intracellular Arg to ornithine and urea, and catabolism leads to Arg depletion thereby enhancing the Warburg effect to promote tumor cell growth [118]. In contrast, ARG-1 promotes the polarization of the M2 phenotype, which causes immune escape by inhibiting T cell function causing immune escape [87]. Ornithine produced by spermidine catabolism can be converted by ornithine decarboxylase (ODC) to polyamines, including putrescine, spermidine, and spermine, promoting M2 macrophage polarization to promote tumorigenesis [119, 120].

In the TME, macrophages can metabolize tryptophan (Trp) to cause immunosuppression [121], a process that is mainly mediated by the metabolism of Trpase (IDO) to produce kynurenine, an endogenous aromatic hydrocarbon receptor (AHR) ligand, which is effective in suppressing the immune response of T cells [122]. IFN-c can activate IDO, and then the overexpression of IDO promotes the macrophage tendency towards an M2 phenotype [123]. In addition to the three amino acids mentioned above, recent studies have also found that Trp metabolism can influence macrophage polarization by affecting interferon synthesis, increasing IGF1 expression through S-adenosylmethionine-dependent promoter abundance of histone H3 lysine 27 trimethylation, and then IGF1 activation of the p38-dependent JAK-STAT1 axis to promote macrophage M2 polarization [124] (Table 2).

**Table 2:** Metabolic targets related to macrophages

Cell	Effect	Metabolism type	Target	Mechanism	Refs.
Macrophage	Antitumor	Glucose metabolism	Glycolysis	Enhancing energy supply via glycolysis supports a stronger immune response	[86]
			OXPHOS	Coupling with glycolysis and production of reactive oxygen species (ROS)	[89]
			HIF-1α	GS inhibition impairs tumor cell motility	[90]
		Lipid metabolism	IFN-β	Inducing the recruitment of natural killer cells to exert their antitumor effect	[91]
			PI3K	Promoting the synthesis of prostaglandin E2 (PGE2); Regulating the polarization of macrophages to the M1 phenotype	[91]
			LOX	Affecting the tumor immune response	[93]
		Amino acid metabolism	NO	The iNOS pathway metabolizes Arg into NO	[98]
	Oncogenic	Glucose metabolism	HK2	Increasing PD-L1 expression	[101]
			Zeb1 transcription factor	Promoting glycolytic activity	[102]
			Lactic acid	Forming an acidic and hypoxic environment by glycolysis accumulates	[101]
			HIF-1α	Enhancing glycolysis by activating Toll-like receptor/nuclear factor-kB (NF-kB), reprogramming to the M2 phenotype occurs	[104]
			IL-10、TGF-β	Inhibiting the activity of T cells reduces the anti-tumor immune response	[106]
		Lipid metabolism	CD36	Promoting tumorigenesis	[108]
			LAL	Degrading fatty acids to support the energy demands of tumor cells	[108]
			ABHD5, MGLL, ACADM	Promotes immune escape by upregulating the metabolism of tumor cells	[86]
			COX-2	Promoting M2 polarization of macrophages	[94]
			IL-4	Inhibiting IFN-γ expression promotes the generation of the M2 phenotype	[114]
			CPT1A	Causing immune escape	[110]
			MGLL	Leading to the accumulation of lipids, causing immune escape	[111]
			RIPK3	Promoting the M2 polarization of FAO-induced macrophages	[112]
			ABCA1/G1	Promoting IL4-mediated IFN-γ suppression leads to the formation of a M2 macrophage phenotype	[88]
		Amino acid metabolism	GS	Inhibiting macrophages tends towards the M1 phenotype	[90]
			αKG	Promoting tumor progression	[116]
			SLC7A11	Transporting glutamine outside the cell can power tumor cells	[117]
			ARG-1	Leading to Arg depletion and enhancing the Warburg effect	[87]
			ODC	Promoting the polarization of M2 macrophages	[119, 120]
			IDO	Inhibiting the immune response of T cells	[122]
			IGF1	Promoting macrophage M2 polarization	[124]

### Neutrophils

#### Introduction to neutrophils

Neutrophils are the most numerous leukocytes in the human body, after their development and maturation in the bone marrow, they are released into the bloodstream and then play the role of innate immunity, mainly involved in acute infections, trauma and other processes, and their ability to promote angiogenesis is closely related to the development and metastasis of tumors, in which neutrophils related to the tumorigenesis of the cancer associated with the tumor-associated neutrophils (TAN), and it has a heterogeneity, N1 phenotype has an anti-tumor effect, N2 phenotype has a tumor-promoting effect [125]. The metabolism of neutrophils changes with their growth and differentiation. When they are still located in the bone marrow, i.e., as hematopoietic stem cells, their central metabolism is glycolysis due to the hypoxic environment. When the bone marrow is stereotyped, it undergoes the TCA, which provides oxidative energy for the activities of the neutrophils [126].

#### Antitumor metabolic processes among neutrophils

Neutrophils can acquire ATP through glycolysis and exert anti-tumor effects [127]. First of all, they can be elevated by the accumulation of GLUT1 in the cell membrane to regulate phagocytosis [128]. In hyperglycemic tumor-bearing mice, neutrophils can be energized by glycolysis to produce G-CSF, which kills tumors through cytotoxicity [119]. In recent years, it has also been found that glycolysis may be related to the immune memory of intrinsic immune cells, through neutrophils from mice treated with β-glucan exerted anti-tumor effects in a ROS-dependent manner after transferring to recipient mice, which may be related to neutrophil reprogramming [129].

In addition to glycolysis, the pentose phosphate pathway (PPP) of neutrophils is also related to their anti-tumor process, and its main way of killing tumors is to metabolize ROS to inhibit proliferation [127]. G-CSF can stimulate PPP to produce ROS in neutrophils, and can also prompt neutrophils to undergo reprogramming to become anti-tumor phenotype [130]. Neutrophil extracellular traps (NETs) are killing complexes composed of histones and antimicrobial peptides produced by neutrophils. In breast cancer, neutrophils were found to be able to use glycolytic metabolism intermediates in PPP for NETs production. When glycolysis is inhibited in neutrophils, the production of NETs by neutrophils is also reduced, indicating a strong link between glycolysis and ROS production [131]. G-CSF can stimulate PPP production of ROS and also promote reprogramming of neutrophils to an anti-tumor phenotype, and it’s closely associated with this progress [132].

Lipid metabolism in neutrophils tends to act as a complementary metabolic component to glycolysis, and is equally capable of producing ROS for tumor killing [133]. Fatty acid metabolism tends to be upregulated in neutrophils in the TME, blocking tumor growth in a T-cell-dependent manner [134]. Other lipid metabolism in neutrophils plays a role mainly in tumor promotion, as will be described later.

Amino acid metabolism is an essential metabolic pathway in neutrophils, and the main one related to antitumor immunity is Arg metabolism. Neutrophils contain ARG-1 to catabolize Arg, and its associated endoplasmic reticulum (ER) stress pathway can induce apoptosis in cancer cells. It has been shown that ARG-1 and Arg deprivation released from neutrophils enhance the ER targeting of antitumor alkyl phospholipid analogs on pancreatic cancer cells [135].

#### Tumor-promoting metabolic processes among neutrophils

Neutrophil glucose metabolism plays a pro-tumorigenic role, mainly in the TME. Firstly, regarding glycolysis, recent studies have shown that neutrophils in the TME express more GLUT1 receptors, which significantly increase the renewal rate of TANs and provide more glucose to the tumor cells to enhance their growth and development [136]. TANs are enriched in LDH activity and pyruvate kinase type M2 dimers, two enzymes that promote the Warburg effect in tumor cells [137]. Neutrophil glycolysis also impairs antitumor activity by generating a glucose-deprived microenvironment, which induces T-cell incompetence by impairing pyruvate kinase M2 and its action on STAT5 [138], and stimulates the expression of HIF-1α in the hypoxic environment of the TME, which then stimulates the expression of PD-L1, inhibiting T-cell activation [139]. As mentioned above, hyperglycemia inhibits the growth of primary tumors, but the number of metastasis-limiting neutrophils in them decreases, leading to easier metastasis and dissemination of tumor cells [140, 141].

Neutrophils undergo the PPP to generate NADPH for the production of ROS, which not only inhibits the proliferation of cancer cells but also inhibits the proliferation of T cells, promoting the immune reprogramming of TANs towards an immunosuppressive phenotype [127, 142]. NO is an important regulator of glucose metabolism. TANs can affect the glucose metabolism of CD8 + T cells by producing NO to cause their apoptosis and inhibit their ability to process, causing apoptosis and inhibiting their ability to delay tumor growth [143].

FAO serves as a metabolic pathway to complement glucose metabolism in neutrophils, and neutrophils do not just use one type of lipid. In 4T1 breast cancer, c-Kit + neutrophils can produce NADPH through fatty acid metabolism and subsequently produce ROS to promote immunosuppression [133]. FAO is also an important metabolism in neutrophil growth and development, and the provision of fatty acids promotes the differentiation of neutrophils into TANs in the TME, which leads to T-cell suppression [144] Arachidonic acid is also a substrate for neutrophil metabolism. It can metabolize PGE2, a substance that mediates immunosuppression. PGE2 has an inhibitory effect on the generation of ROS, leukotriene biosynthesis, and migration of neutrophils, which is achieved through the action of PGE2 on the EP1 and EP2 receptors [145], and through the inhibition of the ROS mentioned just now, the metabolic effect of arachidonic acid on neutrophils can be inhibited. The metabolism of arachidonic acid exerts an immunosuppressive effect on neutrophils [146]. GM-CSF can stimulate the production of fatty acid transporter protein 2 (FATP2) receptors in neutrophils to enable them to transfer more arachidonic acid to promote tumor cell growth [146].

Accumulation of lipids may be closely linked to tumor metastasis [147], lipoproteins are carriers of transported lipids in the blood, and the synergistic role of lipoprotein metabolism with neutrophils in tumors is the key to understanding tumor metabolism, In a study of neutrophil to HDL cholesterol ratio in patients with hepatocellular carcinoma, patients with a higher NHR had a higher risk of death, perhaps related to the involvement of lipoproteins in Tumor metastasis [148]. LOX-1 is expressed in neutrophils and promotes the differentiation of neutrophils into immunosuppressive phenotypes. It was reported that tumor cells can promote the up-regulation of LOX-1 in neutrophils to promote tumor growth [149], and the production of LOX-1 is derived from the ER, which induces a tumorigenic response to ER stress by placing the neutrophils into the ER. Exposure of granulocytes to the conditioned medium from squamous carcinoma cells treated with ER stress inducers enhances the T cell-suppression activity of neutrophils, highlighting a potential link between ER stress and lipid metabolism in neutrophil-mediated tumor promotion [127].

Amino acid metabolism in neutrophils can also play a pro-tumor role in addition to the anti-tumor effects mentioned above. Neutrophils utilize glutamine for metabolism, and glutamine catabolism produces NADPH for the synthesis of ROS, which is capable of suppressing tumor immunity in the same way as described above [150, 151]. Glutamine promotes the development of an immunosuppressive phenotype in neutrophils by activating the PSTAT3/RAB10/ARF4 signaling pathway. Application of glutamate-releasing inhibitors restored neutrophil-killing function and significantly reduced tumor volume [152]. Neutrophils can produce ARG-1, which competes with T cells for Arg metabolism. Since Arg is an important metabolite for T cells to perform their killing function, it can play a role in both tumor suppression and promotion [153]. In breast and lung cancer models, TGF-β can stimulate the TAN to express ARG1, which restricts the immune-suppressing effect of T cells [154]. In addition. Leucine can upregulate the expression of antigen-presenting molecules (e.g., HLA-DR) in neutrophils and enhance their anti-tumor ability. Leucine influences genomic epigenetic modifications by remodeling mitochondrial function and regulating acetyl-CoA production. Leucine-enriched diets or CD74 + neutrophil overdose therapy also significantly improved the efficacy of anti-PD-1 therapies [155]. Multiple neutrophil subpopulations in the TME have different properties, among which amino acids can activate the HLA-DR + CD74 + subpopulation to have significant antigen-presenting and anti-tumor functions. These subpopulations are abundant in patients with good prognosis, demonstrating their potential as immunotherapeutic targets [156], and many amino acid components, such as glutamate and Arg, can reprogram neutrophils in the TME, which can have an important effect on tumor cell proliferation, survival, and immune escape [157] (Table 3).

**Table 3:** Metabolic targets related to neutrophils

Cell	Effect	Metabolism type	Target	Mechanism	Refs.
Neutrophils	Antitumor	Glucose metabolism	GLUT-1	Transporting glucose for glycolysis to fuel phagocytosis	[128]
			glycolysis	Producing G-CSF, which kills tumors through cytotoxic effects	[130]
			PPP	Reprogramming of neutrophils into an anti-tumor phenotype and produce NETs	[131]
		Lipid metabolism	–	Producing ROS and enhancing T cell–mediated tumor killing	[134]
		Amino acid metabolism	ARG1	Inducing apoptosis of cancer cells	[135]
	Oncogenic	Glucose metabolism	GLUT-1	Increasing the turnover rate of TANs	[136]
			PKM2	Promoting the Warburg effect in tumor cells and Inducing T cell incompetence	[137]
			LDH	Promoting the Warburg effect in tumor cells	[137]
			HIF-1α	Inhibiting the activation of T cells	[139]
			PPP	Inhibiting T cell proliferation and promoting an immunosuppressive phenotype	[127, 142]
			NO	Inhibiting CD8 + T’s ability	[143]
		Lipid metabolism	c-Kit(CD117)	Producing NADPH and subsequently ROS	[133]
			EP1/EP2	Inhibiting the production of reactive oxygen species, leukotriene biosynthesis	[145]
			FATP2	Promoting tumor cell growth	[146]
			HDL	Promoting tumor cell growth	[148]
			LOX-1	Promoting the differentiation of neutrophils into an immunosuppressive phenotype	[149]
		Amino acid metabolism	Glutamate	Activation of the pSTAT3/RAB10/ARF4 signaling pathway	[152]
			ARG1	Competing with T cells for the metabolism of Arg	[153]
			Leucine	Upregulating antigen presentation molecule expression to promote antitumor immunity	[155]

### Dendritic cells

#### Introduction to DCs

DCs are important members of the intrinsic immune cells and play a major role in the immune response by presenting antigens and transmitting the presentation information to T cells [158]. During the maturation process of DCs, DCs need a lot of energy, so they mainly rely on OXPHOS in the immature period. In the maturation process, their main metabolic process is slowly converted to glycolysis because the latter can give more energy to DCs [159]. In the process of tumor immunity, the main role of DCs is to present tumor cell-associated antigens to T cells, which can help them carry out specific anti-tumor immunity process after the recognition and activation of DCs by TOLL-like receptors (TLRs) [160–162]. The DCs undergoes the above mentioned metabolic changes to promote maturation, and after the response of the ligand of the TLRs, the DC up-regulates the PI3K/Ser/AKT pathway and TBK1- IKKε pathway mediate the uptake of several nutrients such as glucose by DCs and begin to function as antigen presenters [163].

According to the role of DCs in tumor metabolism, they can be classified into three subtypes: cDC1, cDC2, and pDC (plasma cell-like DCs), and have a role in anti-tumor effects, tumor progression, and metastasis [164].

#### Antitumor metabolic processes among DC

As mentioned above, DCs rely on glycolysis to fulfill their antigen-presenting role. After TLR activation, DCs undergo glycolysis rapidly. It is divided into two pathways: one is rapid glycolysis within a few minutes of activation, which is mainly used to satisfy the energy requirements for DCs maturation; the other is glycolysis that produces carbon monoxide to satisfy the energy requirements for subsequent immune function. In antitumor glycolysis, DCs utilize glucose mainly from intracellular glycogenolysis [163].

On the one hand, the DCs glycolysis mainly provides energy for cytokines induced by TLR receptors, such as IL-12 and Th-1. Meanwhile, other studies have shown that inhibition of glycolysis for some time after DCs activation enhances the anti-tumor function of DC-induced T cells [165]. Glycolysis produces lactate, which is metabolized by DCs and promotes tumor growth via G protein-coupled receptor (GPR81), a G protein-coupled lactate receptor. Lactate activates GPR81 in mouse DCs to inhibit the delivery of MHC II on the DC surface. Still, lactate controls this in both directions by paracrine activation of GPR81 on stromal DCs, inhibiting tumor growth and immune evasion. An immune evasion that inhibits tumor growth [166]. ROS produced by DC are important for activating the adaptive immune system. Still, the amount of ROS they produce is usually small [167], and these ROS activate the mechanism of DCs anti-tumor function by regulating the STING signaling pathway. This study found that ROS, as metabolic by-products, enhance the ability of DCs to sense DNA-related signals and their anti-tumor effects [168].

DCs exhibit significant lipid metabolic features in tumor immunity. In the TME, fatty acid β-oxidation is an important metabolic pathway for DCs function. It helps maintain their maturation state and promotes anti-tumor immune responses [169], and cholesterol is an important regulator of DCs function. Studies have shown that high cholesterol levels in the TME may lead to inhibition of DCs function, whereas the antigen-presenting capacity of DCs can be enhanced by promoting cholesterol efflux or reducing its accumulation [170].

The process of glycolysis has been shown to enhance subsequent lipid synthesis, which facilitates DCs to continue synthesizing relevant immune protein molecules involved in the anti-tumor immune response [171].

Amino acid metabolism in DCs is an important factor affecting their development, function, and anti-tumor immunity, with special regulatory significance, especially in the TME. Glutamine is an important metabolic fuel for DCs function, and its metabolism affects the antigen-presenting capacity of DCs by supplying energy and supporting biosynthetic processes.

In the TME, glutamine competition leads to impaired DCs function. Still, studies have shown that catabolism provides a carbon source for the TCA, supports DCs energy requirements and metabolic reprogramming, and promotes DCs antigen presentation function [172]. Besides glutamine, Arg is another key amino acid involved in DCs maturation and T cell activation. Nitric oxide synthase (NOS) and ARG-1 are two major Arg metabolizing enzymes, both of which play essential roles in the antitumor activity and immunosuppressive effects of DCs: high levels of NO can inhibit tumor growth by regulating DCs function; Arg metabolism indirectly affects the antitumor ability of DCs by regulating their maturation and functional status, and also by modulating T cell activation and proliferation [173]. In addition to the above two common amino acids, serine/threonine metabolism is also related to the antitumor effects of DCs. Insufficient one-carbon metabolism significantly impairs the antitumor immune function of DCs. Serine is essential for DCs proliferation and function through its involvement in one-carbon metabolism in support of DNA and lipid synthesis [174]. STK11 can encode tumor suppressor liver kinase b1(Lkb1), an important enzyme that promotes dendritic cell maturation and functions as a tumor antigen presenter. So the deficiency of STK11 may lead to spontaneous tumor formation [175].

#### Tumor-promoting metabolic processes among DCs

Glucose metabolism promotes tumors mainly related to several mechanisms, when DCs are activated, it can stimulate the anti-tumor effect of T cells, but the activity of T cells will accelerate the competition for glucose and inactivate the mTORC1/HIF-1α/NOS2 glucose-sensing signaling pathway, leading to the dysfunction of DCs [176]. Lactose is a product of DCs glycolysis. Lactate can promote tumor growth by activating the GPR81 protein receptor on the DC surface and inhibiting its function of antigen presentation [177], and its mechanism may be related to accelerating the degradation of antigens through the inhibition of type I IFNs downstream of TLR3 and STING by lactate [178, 179]. At the same time, lactate attenuates the intracellular Ca2 + mobilization triggered by the DC cell-surface GPR81 receptor. The induction of IFN-α by DCs is mediated by intracellular Ca2 + mobilization, which is triggered by the GPR81 receptor on the DC surface. At the same time, lactate can be directly imported into DCs via monocarboxylate transporter proteins. This import affects their metabolic capacity and, in turn, inhibits the activation of antitumor immune cells. In addition, lactate enhances the Trp metabolism and kynurenine production of DCs, which induces immune-suppression production in Tregs [180]. Besides the above two mechanisms, lactic acid can make the tumor microenvironment acidic, and the charge in the acidic environment can affect the binding of DCs to antigens, leading to the instability of their complexes, which affects their antigenic presentation and the subsequent process [181].

In addition to the effect of lactate, DC glycolysis can promote tumor growth through some other related pathways: DC glycolysis can be reprogrammed by HIF-1, and in a recent study, it was found that CCR7 chemokines activate the HIF-1α transcription factor pathway in DC, and depending on this pathway, DC can undergo glycolytic reprogramming and migrate for antigen presentation. The non-coding RNA lnc Dpf3 directly binds to HIF-1α and represses HIF-1α-dependent transcription of glycolytic genes, thereby inhibiting DC glycolytic metabolism and migration [182]. It should be noted that the glycolytic metabolism of DC generates a large amount of ROS. However, appropriate ROS can inhibit the growth of tumor cells. The function of DC will be affected accordingly due to the excessively large amount of ROS, especially the high mobility group protein B1 (HMGB1), which is scavenged by ROS, leading to the inability of DC to mature and function normally [183].

Accumulation of lipids plays an important role in influencing the immune presentation function of DCs through a process that mainly impairs T cell-dependent effects on DCs by decreasing the antigen processing capacity of DCs [184], down-regulating the co-stimulatory molecule CD86, and overexpressing the tolerogenic cytokine IL-10 [185]. It was found that FASN is a key enzyme in FAS. Increased activity of FASN leads to increased FAS in tumor tissues, and high concentration of fatty acids in TME leads to accumulation of fatty acids in DCs, which affects their function [186]. Radiation-induced DCs also showed an abnormal accumulation of lipids, resulting in the inability to present tumor antigens to T cells, revealing the association between radiation carcinogenesis and DCs [187]. Lipid peroxidation promotes the activation of X-box binding protein 1 (XBP-1), a key mediator of tumor activation. Under oxidative stress induced by ROS, XBP-1 enhanced lipid biosynthesis, leading to lipid accumulation and impairing the antitumor effects of T cells [188].

FAO is another pro-tumor metabolism in DCs. It has been found that melanoma can promote FAO in DCs through the PPAR-γ signaling pathway, which can upregulate the expression of CPT1A fatty acid transporter protein. This, in turn, suppresses the production of the cytokines IL-6 and IL-12, thereby affecting the production of T-cells [189]. Peroxisome also activates PPAR-α in response to fatty acids, which promotes metabolic reprogramming of DCs undergoing FAO, leading to DC immune dysfunction [190].

Sterol metabolism mainly inhibits the antitumor effects of DCs. Tumor-secreted factors can promote cholesterol accumulation in DCs, leading to reduced MHC-I expression, and cause impaired antigen presentation and inability to activate T-cells [191]. It has been reported that Liver X Receptor (LXR) can inhibit the expression of CC Chemokine Receptor-7(CCR7), which promotes the chemotactic migration of DCs. By suppressing CCR7, LXR impairs DCs migration, which eventually leads to immune escape [192].

Among amino acid metabolism, Trp metabolism is important in DCs tumor promotion. IDO-1 catalyzes the kynurenine pathway of Trp degradation, which can trigger the onset of tumor immunosuppression [193]. The pathway produces 3-hydroxy-octylamino benzoic acid, a metabolite increasing Foxp3 + Treg cell appearance in an NCOA7-dependent manner, contributing to an immunosuppressive tumor microenvironment [194]. When Trp is depleted to a certain level, it activates serine-threonine kinase 2 (GCN2), a metabolic stress sensor, and GCN2 is capable of causing dysfunction and immune escape by activating immune reprogramming in DCs [195].

In addition to Trp, Arg metabolism in DCs also promotes tumor growth, and ARG-1 in DCs can catabolize Arg to polyamine production. Polyamines can regulate DCs immune reprogramming to an immunosuppressive phenotype through activation of steroid receptor coactivator 1 kinase [196].

Hypoxia is also a metabolic process that promotes tumor development in DCs. HIF-1α can inhibit the pharmacological effects of dendritic vaccines in a 4T1 breast cancer model, suggesting that hypoxia may inhibit the anti-tumor effects of DCs to act as a tumor-promoting agent [197] (Table 4).

**Table 4:** Metabolic targets related to DCs

Cell	Effect	Metabolism type	Target	Mechanism	Refs.
Dendritic cell	Antitumor	glucose metabolism	TLR	Producing IL-12, Th-1 and other cytokines to exert anti-tumor effects	[165]
			GPR81	Inhibiting tumor growth	[166]
			STING	Activating the antitumor function of DCs	[168]
			CCR7	Leading to reprogramming of glucose metabolism and migration for antigen presentation	[182]
		Lipid metabolism	–	Maintaining their mature state, promoting antitumor immune responses, and enhancing antigen-presenting capacity	[169]
		Amino acid metabolism	NOS	Inhibiting tumor growth	[173]
			ARG-1	Affecting the antitumor ability of DCs	[174]
			Stk11	Encoding Lkb1	[175]
	Oncogenic	Glucose metabolism	mTORC1/HIF-1 α/NOS2	Leading to a dysfunction of DCs	[176]
			GPR81	Promoting tumor growth and inhibiting antigen presentation function	[177]
			TLR3	Downstream targets of GPR81	[178, 179]
			IFN-α	STING downstream target, accelerates antigen degradation	[180]
			lnc-Dpf3	Inhibiting HIF-1a-dependent transcription of glycolytic genes	[182]
			HMGB1	Leading to abnormal maturation and function of DCs	[183]
		Lipid metabolism	CD86	Reducing DC antigen processing capacity	[184, 185]
			FASN	Affecting DCs function	[186]
			XBP-1	An important mediator of tumor activation	[188]
			PPAR-γ	Inhibiting the expression of IL-6 and IL-12 cytokines and affecting T cell production	[189]
			PPAR-α	Promoting FAO and leading to immune dysfunction in DCs	[190]
			cholesterol	Inhibition of DC function	[191]
			LXR	Suppress DC expression of CCR7	[192]
		Amino acid metabolism	IDO1	Stimulating the occurrence of tumor immunosuppression	[193]
			3-HAA	Increasing the appearance of Foxp3 + Tregs to exert immunosuppressive functions	[194]

### NK cells

#### Introduction to NK cells

NK cells can spontaneously kill target cells without prior antigenic stimulation and are widely distributed in the blood, peripheral lymphoid tissues, liver, and spleen. The metabolism of NK cells continues to change as they grow in the bone marrow [198–200]. In the early stages of growth, NK cells express amino acid transporter proteins during early development stages to maintain the metabolic required for their growth. As they mature, NK cells become less dependent on glycolysis and glucose uptake [201]. It has also been shown that after NK cells reach maturity, their metabolic state is maintained at a low level, including glycolysis and OXPHOS. These low levels of metabolism results in a decrease in the level of interferon secretion by the NK cells [202]. There are two different subpopulations of NK cells, CD56bright and CD56dim, and these two phenotypes of NK cells have different functions. CD56bright is mainly known to secrete cytokines for tumor immunity, so the metabolic level of this phenotype is relatively higher [203].

#### Antitumor metabolic processes among NK cells

Hypoxia is an important feature of the TME and is of great significance for the activation of NK cells. HIF-1α can promote the IL-2 and IL-12 cytokine-stimulated OXPHOS response, and promote the killing effect of NK cells on the tumor tissues [204]. It can also allow NK cells to transform from the OXPHOS metabolism to glycolysis in the early hypoxic environment. And after the transformation, NK cells can obtain more powerful killing power, which enhances their cytotoxicity against tumor cell lines such as K562, CEM, and A375 [205]. However, the effects of hypoxia on NK cells are two-sided. Hypoxic conditions and lactic acid accumulation impair mitochondrial function and induce apoptosis in NK cells [206]. These stressors also promote the fragmentation of dynamin-related protein 1 (mTOR-Drp1), a process regulated by mTOR signaling, thereby disrupting normal energy metabolism [207], and adenosine is produced in large quantities in cells following hypoxia. These metabolites inhibit the NK cell-dependent IL-2 activation pathway, resulting in immune suppression [208]. More directly, a research team inhibited the expression of HIF-1α and found that the tumor-killing factors secreted by NK cells were increased after the inhibition, which also illustrates the double-sided role of hypoxia in the anti-tumor effects of NK cells [209]. After the above transformation from OXPHOS, NK cells'anti-tumor metabolism is mainly glucose metabolism. Under the stimulation of cytokines, NK cells'surface expression of GLUT1 protein will be transported to the intracellular supply of energy and play an anti-tumor role [210]. Fructose-1,6-bisphosphatase (FBP), in this process, activates NK cells, giving them a more potent anti-tumor effect, especially the production of IFN- γ. SREBP, a cytokine that regulates glycolysis and OXPHOS in NK cells, is associated with IFN- γ production, which is essential for the cytotoxicity of NK cells [211]. The mTORC1 mentioned above, can promote central metabolic regulators such as the glycolytic pathway and can participate in NK cell activation and metabolic reprogramming, promoting the antitumor effects of NK cells [212].

NK cell-associated lipid metabolism was thought to be related to the inhibition of their tumor-killing function in the past. Still, recent studies have uncovered this complexity, with NK cells, like T cells, can express TLRs and recognize lipid antigens. The TLRs have a diversity, either semi-invariant (type I) or varied (type II), wherein type I can promote anti-tumor immunity in NK tumors by recognizing the lipid antigens and generating a series of metabolic responses [213].

Amino acids are important metabolic raw materials for NK cells, and glutamine is the main amino acid related to their anti-tumor activity. Glutamine metabolism can regulate c-Myc transcription factor in NK cells, which can induce NK cells to secrete IL-2 and IL-12 to kill tumors [204]. It can also stimulate the up-regulation of glycolysis through the mTOR pathway to enhance the anti-tumor ability of NK cells. Meanwhile, in addition to killing tumors, IL-2/12 can stimulate NK cells to carry out metabolic reprogramming to increase glycolysis activity. Glutamine metabolism can also promote the up-regulation of c-Myc to stimulate the anti-tumor metabolism of NK cells. However, studies have shown that glutamine is not an indispensable nutrient for the anti-tumor effect of NK cells, and it is unnecessary for NK cells to carry out anti-tumor effects. 

#### Tumor-promoting metabolic processes among NK cells

NK cells'glucose metabolism is the key to their cytotoxicity, but in the TME, tumor cells compete with other immune cells for glucose. Glucose depletion leads to a series of weakening of mTOR activity, glycolytic capacity, and IFN-γ production, resulting in anti-tumor dysfunction of NK cells [214]. Glycolytic intermediates and small molecules can also inhibit NK cell function. In Kras-driven lung cancer, it has been found that the presence of aberrant FBP in NK cells can inhibit glycolysis and normal cellular function, which is often a cause of immune escape [215], increasing anti-tumor therapy modalities based on this pathway are being proven to be very effective [203]. The SREBP transcription factor mentioned above induces metabolic reprogramming of NK cells by promoting glycolysis and the citrate-malate shuttle pathway. However, interferons in the TME, such as 27-hydroxycholesterol, can inhibit this transcription factor and cause immune escape [216, 217]. The mTOR is a serine/threonine kinase that promotes glycolytic pathways and plays a key role in the immune defense system. The mTOR is divided into two types: mTOR1 and mTOR2. mTOR2 inhibits the expression of SLC7A5 in STAT5 to inhibit the glycolysis of NK cells regulated by mTORC1 [218]. In addition to other regulatory mechanisms, mTOR1 is also inhibited by TGF-β, a major immunosuppressive cytokine in the TME. Conversely, mTOR activation induced by IL-15 can suppress antitumor immunity [219].

In addition to glucose metabolism, the accumulation of lactate produced by NK cell glycolysis is also an essential factor causing immune escape. The TME is acidic due to the accumulation of lactate, which can inhibit the cytotoxicity of NK cells [220]. In addition, the accumulation of lactate can lead to a decrease in the production of IFN-γ through the up-regulation of the nuclear factor of activated T-cells (NFAT) in the tissues, which results in the immune evasion by the tumors [221]. Ocular sinus homology cassette homolog 1 (SIX1) is closely associated with NK cell dysfunction in tumor tissues. In pancreatic cancer, SIX1 directly binds to the promoter region of LDHA, triggering glycolysis in tumor cells to promote lactate accumulation, leading to NK cell dysfunction [222].

Lipid metabolism is an important energy source for NK cells, but excessive accumulation of lipids is the main reason affecting their metabolic function. Lipids can affect the relevant anti-tumor functions of NK cells through the lipid antigen repertoire and the regulation of CD1d expression [213], PPAR-γ can up-regulate CD1d to promote the lipid metabolism of NK cells, however, recently some researchers found that lipid accumulation may inhibit this expression process, leading to suppression of the normal anti-tumor function of NK cells [223]. In addition to this aspect, lipid accumulation can lead to sustained NK cell dysfunction by inhibiting P300-mediated c-Myc acetylation [224].

Arg and Trp metabolism are closely linked to inhibiting NK cell function. In the TME, tumor cells compete with NK cells for Arg sources, and Arg depletion reduces the expression of NK-92 cell-activated receptors, NKp46 and NKp30, as well as the production of IFN-γ, which leads to the dysfunction of NK cells [225]. Recently, it was shown that the mitochondrial damage-associated molecular pattern (mitoDAMP) can affect the cytotoxicity mechanism of NK cells via mitochondrial damage, and mass spectrometry analysis of mitoDAMP reveals that it is metabolized by highly active Arg, which confirms the ability of Arg to cause NK cell dysfunction through mitochondrial damage [226]. Trp and kynurenine pathways have been implicated in developing many immune cells and tumors. In NK cells, kynurenine can cause apoptosis through ROS. On the other hand, it can also affect the expression of NK cell activation receptor NKG2D and natural cytotoxicity triggering receptor 1 (NCR1, also known as NKp46), resulting in impaired activation of NK cells and facilitating the occurrence of immune escape [203].

**Fig. 4:**
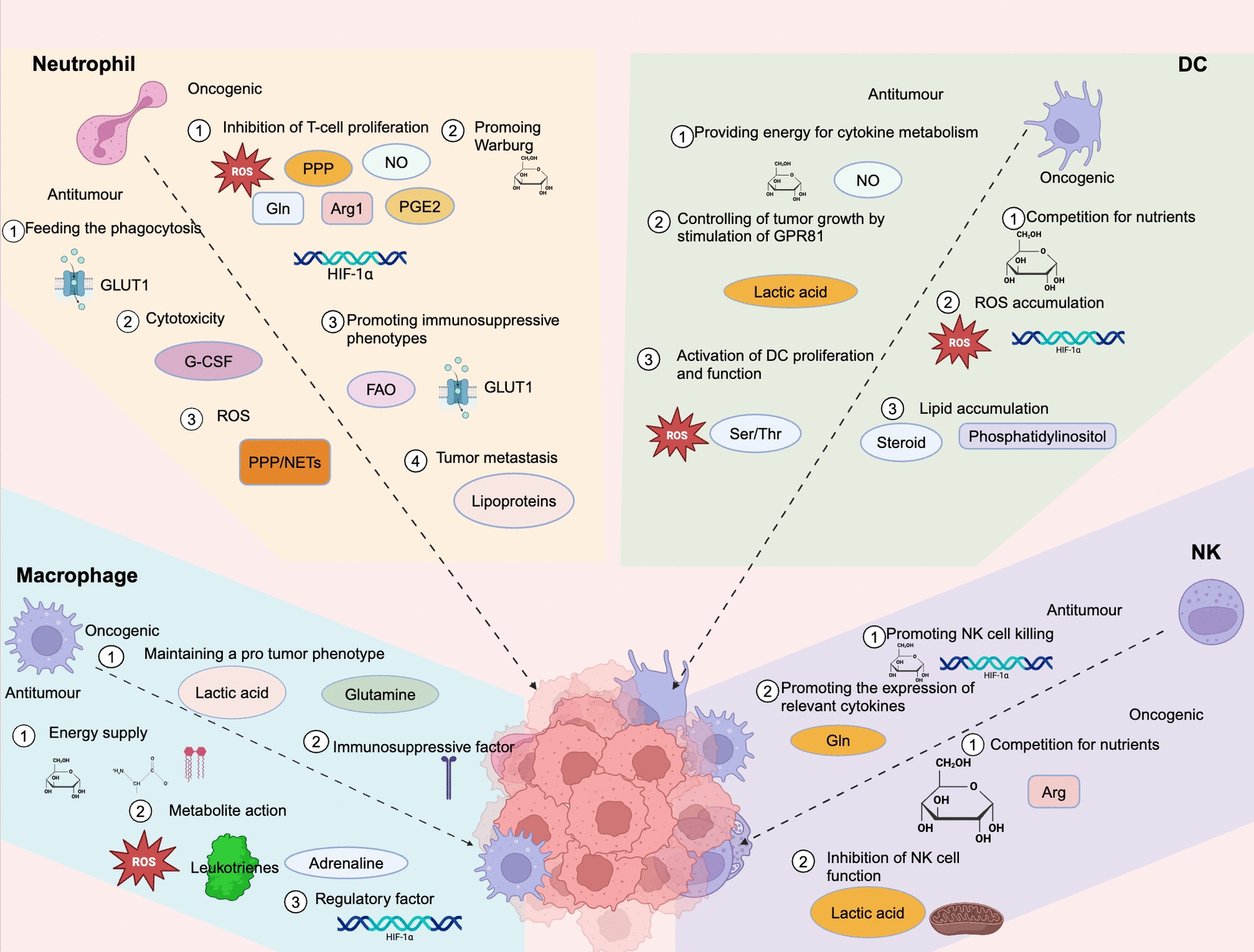
The anti-tumor and pro-tumor metabolic functions of four types of innate immune cells: macrophages, neutrophils, dendritic cells, and NK cells. In macrophages, anti-tumor effects are linked to energy supply, inhibitory metabolic products, and immunoregulatory factors, whereas pro-tumor functions are associated with supporting immunosuppressive phenotypes and the production of various factors. For neutrophils, anti-tumor activity is connected to energy metabolism, metabolic toxicity, and the generation of reactive oxygen species (ROS). Conversely, their pro-tumor roles involve the suppression of T cells, the promotion of the Warburg effect, and support for immunosuppressive neutrophils. Dendritic cells exhibit anti-tumor responses through the metabolic activation of cytokine secretion and GPR81-mediated tumor control; however, their pro-tumor effects arise from nutrient competition, ROS production, and lipid accumulation. Lastly, NK cells demonstrate anti-tumor functions through enhanced cytotoxicity and cytokine production, while their pro-tumor effects stem from nutrient deprivation and metabolic inhibition. Created with BioRender.com. *ROS* Reactive Oxygen Species, *PPP* Pentose Phosphate Pathway, *NO* Nitric Oxide, *Gln* Glutamine, *Arg1* Arginase 1, *DC* Dendritic Cell, *NK* Natural Killer Cell, *FAO* Fatty Acid Oxidation

In summary, NK cells possess powerful anti-tumor functions. Still, due to the acidic as well as hypoxic environment of the TME, their normal metabolism is impeded, thereby causing immune escape from the tumor. (Fig. 4) (Table 5).

**Table 5:** Metabolic targets related to NK cells

Cell	Effect	Metabolism type	Target	Mechanism	Refs.
NK cell	Antitumor	Hypoxia	HIF-1α	Promoting the killing of tumor tissue by NK cells	[204]
			mTOR-Drp1	Preventing NK cells from carrying out the corresponding energy metabolism	[207]
		Glucose metabolism	FBPase	Activating NK cells	[210]
			SREBP	An essential component in regulating glycolysis and OXPHOS in NK cells	[211]
			mTOR	Enhancing the anti-tumor effect of NK cells	[212]
		Lipid metabolism	TLR	Promoting anti-tumor immunity in NK tumors	[213]
		Amino acid metabolism	c-Myc	Promoting NK cells to secrete IL-2 and IL-12 to kill tumors	[204]
	Oncogenic	Glucose metabolism	27-hydroxycholesterol	Inhibiting the SREBP transcription factor	[216, 217]
			mTOR2	Inhibiting mTORC1-regulated NK cell glycolysis	[218]
			TGF-β	Blocking IL-15-induced mTOR activation leads to suppressed tumor immunity	[219]
			NFAT	Reducing the production of IFN-γ and causing tumor immune escape	[221]
			SIX1	Leading to dysfunction of NK cells	[222]
		Lipid metabolism	CD1d	Influencing the relevant antitumor function of NK cells	[223]
			PPAR γ	Upregulating CD1d to promote lipid metabolism in NK cells and promote tumor growth	[223]
			c-Myc	Inhibiting c-Myc acetylation and leading to persistent NK cell dysfunction	[224]
		Amino acid metabolism	NKp46/NKp30	Causing dysfunction of NK cells	[225]
			NKG2D	Leading to impaired activation of NK cells and promoting immune escape	[203]

### Mast cells

Mast cells are widely distributed in the skin and visceral submucosa, and are involved in immune regulation after pathogen infection and allergic reactions through the secretion of various cytokines. From the first mast cell being discovered. In 1878, Paul Ehrlich conducted extensive research that showed that mast cells also have an important link with tumor progression [227], especially the regulation of the TME [228]. In previous studies, mast cells have predominantly been characterized as auxiliary or passive players in tumor development, without directly contributing to tumor inhibition or progression. But recent years, metabolic studies have revealed that mast cells can modulate the TME through multiple pathways.

#### The metabolism of the mast cell

Mast cells originate from hematopoietic stem cells, which tend to be resting to prevent ROS damage caused by mitochondrial respiration and, therefore, rely mainly on glycolysis for energy at this stage. However, due to the accumulation of ROS induced by aberrant mitochondrial respiration, hematopoietic stem cells undergo growth and differentiation, which is metabolized in a variety of ways, including glycolysis, OXPHOS, etc. At the same time, their maturation needs to be activated by relevant receptors, particularly the TME. In addition, its maturation requires the activation of relevant receptors, especially the degranulation of IgE, a process that enhances the activity of glycolysis in mast cells, and the initiation of OXPHOS in a glucose-poor environment, both of which are important for mast cell development and the fulfillment of their primary functions [229]. Besides the dependence on IgE, IL-33 and ATP also activate mast cells to promote glycolysis and OXPHOS, with ATP positively correlating with glycolysis and histamine release from mast cells, indicating its importance. Mast cells influence the TME through degranulation and release of cytokines, and many other connections and functions remain to be investigated. Tumor cells release cytokines to induce the recruitment of nearby mast cells, resulting in tumor infiltration. When stimulated by appropriate signals, mast cells release cytokines to induce the recruitment of nearby mast cells, resulting in tumor infiltration. Among the corresponding particles released by mast cells, suggesting that lipid synthesis may play a crucial metabolic role in the interaction between mast cells and tumors. This process could, in turn, provide a potential therapeutic avenue for targeting mast cells in tumor treatment [230]. There is limited research on the metabolic functions of mast cells and their roles in the TME, possibly due to the intrinsic characteristics of mast cells. However, this highlights a promising direction for future studies to explore the critical relationship between mast cells and inflammation and the link between inflammation and tumor development. We will continue to follow the progress of related research in this area.

Cancer therapy has fully transitioned into the era of genomics and personalized medicine. Substantial progress has been made in elucidating the metabolic reprogramming of immune cells within the TME, leading to critical mechanistic insights and discoveries. These metabolic pathways may represent promising therapeutic targets to improve the efficacy of cancer immunotherapy. Nevertheless, current research predominantly focuses on the crosstalk between individual immune cell types and tumor cells, often neglecting the potential interplay among multiple immune cell populations. Future investigations should aim to delineate the metabolic interactions among diverse immune cells during tumor progression, which will be critical for deepening our understanding and advancing this field.

## Adaptive immune cells

### T cells

T cells can be classified by activation stage into initial T cells, effector T cells (Teff cells), memory T cells (Tmem cells), and CD4+ and CD8+ T cells according to co-receptors [231, 232]. After antigen stimulation, initial CD8+ T cells differentiate into cytotoxic effector cells and long-term memory cells, and initial CD4+ T cells differentiate into Th1, Th2, Th9, Th17, Th22, follicular helper T cells (T_fh_), Tregs, and long-term memory cells [233]. Among them, anti-tumor CD4+ cells such as TH1 are called CD4+ conventional T cells (CD4+ _conv_ T cells) [95]. Tregs are immune-suppressive T cells characterized by high expression of FOXP3, CD25, and CD4, which play an important role in suppressing anti-tumor immunity. They can secrete IL-35 and IL-10 to promote the depletion of T cells in tumor [234].

#### Glucose metabolism in T cells

CD4+ conv T cells and CD8+ Teff cells are key effector cells in the antitumor response. When recognizing their specific antigens and receiving co-stimulatory signals, naïve CD4+ and CD8+ T cells proliferate and initiate metabolic programs that support rapid expansion. In aerobic conditions, most healthy cells generate ATP primarily through glucose-driven OXPHOS. In contrast, cancer cells often exhibit the Warburg effect—a characteristic of cancer cells that rely on aerobic glycolysis to satisfy their energy needs. This metabolic reprogramming is considered a hallmark of malignant transformation [235]. Similar changes in glycolysis also occur in activated T cells. High glycolytic activity in CD8+ T cells facilitates the induction of their differentiation to effector cells. In contrast, changes in glycolysis in CD8+ effector T cells play an essential role in the production of IFN-γ [236]. CD4+ T cells also tend to be metabolized by aerobic glycolysis when differentiating from CD4+ conventional T cells. Upregulation of aerobic glycolysis is a hallmark of T cell activation. Still, it is now shown that upregulation of mitochondrial respiration and OXPHOS is also a key aspect of activating CD4+ conv and CD8+ T cells [237, 238]. Tregs, on the other hand, exhibit a lower rate of glycolysis and a relatively high degree of OXPHOS [239, 240]. Unlike Teff cells, CD8+ Tmem cells preferentially rely on OXPHOS for energy supply [241].

T cell glycolysis is regulated through several mechanisms: Myc, one of the earliest genes activated in T cells, regulates glucose uptake and metabolism by modulating the expression of key glycolytic genes, including GLUT1, LDHA, pyruvate kinase (PKM), and HK2 [242]. HIF-1α is associated with the expression of a wide range of glycolytic enzymes, including HK2, trehalose phosphate isomerase (TPI), enolase 1, and PKM. In contrast, the mTORC1-HIF-1α signaling axis is significantly associated with the metabolic regulation of some subsets of CD4+ T cells and the effector function of CD8+ effector cells [242–244]. mTOR is a key regulator of T cell metabolism and induces the transcription of many glycolytic enzymes [245, 246]. When mTOR signaling is diminished, T cell glycolysis is inhibited [247]. GLUT1 is selectively required in vivo during metabolic reprogramming for CD4+ T cell activation, Teff expansion, and survival [248]. GLUT1 also maintains the anti-apoptotic capacity of CD8+ T cells by activating the PI3K/Akt pathway [249]. GLUT1 overexpression enhances the metabolic capacity and anti-tumor efficacy [250]. GLUT2 regulates the effector response of mouse CD8+ T cells by promoting glucose uptake, glycolysis, and glucose storage [251]. Lactate, the end product of glycolysis, suppresses tumor growth by inhibiting histone deacetylase activity, increasing Tcf7 gene expression, and enhancing CD8+ T cell stemness [252].

In addition to the above regulation, there is also a proximal pathway regulation of T cell glucose metabolism: the PPP occurs in the cytoplasm, starting with the proximal metabolite of the glycolytic pathway, glucose 6-phosphate, which is converted to ribose 5-phosphate, with the concomitant production of NADPH. Glucose 6-phosphate dehydrogenase (G6PD) is the rate-limiting enzyme of the PPP, and the main source of NADPH required to maintain the cellular capacity to scavenge for ROS. G6PD is the rate-limiting enzyme of the PPP and is the main source of NADPH required to maintain the cellular ROS clearance capacity [253]. Glucose entry into the PPP is significantly increased during CD4+ T cell activation [254]. However, blocking G6PD in the oxidative PPP of Tregs leads to a substantial reduction in their immunosuppressive function and a shift towards Th1, Th2, and Th17 phenotypes [255]. Interestingly, inhibition of G6PD in oxidative PPP leads to producing CD8+ T cells with enhanced anti-tumor effects [256]. In addition, the large amount of NADPH produced by PPP ensures high levels of reduced glutathione in Tmem cells, safeguarding their formation and maintenance [257]. Hexosamine biosynthesis pathway (HBP) is a glucose metabolic pathway that synthesizes uridine diphosphate-N-acetylglucosamine (UDP-GlcNAc), which is subsequently used for post-translational modification of intracellular proteins (O-GlcNAcylation) [258]. UDP-GlcNAc is a substrate for cytosolic glycosyltransferases, which promote the proliferation and functioning of CD4+ and CD8+ effector T cells. Immunologically activated T-cells contain higher levels of UDP-GlcNAc than initial T-cells, which supports their proliferation and function [259]. The SGOC metabolic pathway is involved in the biosynthesis of proteins, lipids, and nucleotides and plays a critical role in the maintenance of DNA methylation, histone methylation, and redox homeostasis [260]. T-cells activate to upregulate the enzymes associated with SGOC, and serine provides proliferating T cells with both glycine and one-carbon units for ab initio nucleotide synthesis [261]. Intermediates in the TCA cycle, such as α-KG and succinate, are also important for T cell regulation. α-KG alters the DNA methylation profiles of initial CD4+ T cells activated under conditions of Treg cell polarization and significantly inhibits the differentiation of FoxP3+ Tregs [262]. 2-Hydroxyglutarate (2-HG) is an α-KG mitochondrial derivative. Increasing 2-HG promotes Th17 cell differentiation and inhibits iTreg cell differentiation [263]. CD4+ and CD8+ T cell exposure to tumor-associated succinic acid inhibits degranulation and cytokine secretion, thereby suppressing their antitumor effects [264]. Targeting the regulation of glucose metabolism in T cells during antitumor responses may represent an effective strategy to enhance cancer immunotherapy. Future efforts should focus on identifying and developing additional therapeutic targets based on this approach.

#### Tumor-promoting glucose metabolism in T cells

Insufficient glucose in the TME or impaired glucose uptake by T cells inhibits the effector function of T cells. In case of glucose deprivation in the TME, the glycolytic process of Teff cells is inhibited, which in turn makes the production of ATP and metabolic intermediates insufficient, leading to impaired function or depletion of most CD8+ T cells [265]. Phosphoenolpyruvate (PEP) is a metabolic checkpoint for anti-tumor T cell responses, and low glucose conditions inhibit the production of PEP. This T cell glycolytic intermediate disrupts calcium-dependent NFAT signaling and reduces its anti-tumor effects [266]. In addition to this, reducing glucose in the culture medium can attenuate mTOR signaling and inhibit the function of effector T cells on the one hand, and inhibit the production of key effector molecules such as INF-γ, IL-17, and granzyme B in Teff cells on the other [95]. However, glucose restriction can activate AMPK and downstream SENP1-Sirt3 signaling, which promotes T cell memory formation [267]. Effector T cells can utilize inosine as an alternative substrate to support cell growth and function without glucose in vitro [268].

Tumors usually use glycolysis to promote their growth, while glucose consumption by tumor cells limits T cell metabolism and promotes tumor deterioration by directly inhibiting their effector functions [269]. In addition to this, tumors also cause lactic acid to accumulate within the TME in competing with T cells for glucose, further inhibiting T cell function [270]. Enhanced glycolysis by drug treatment or overexpression of key glycolytic enzymes (e.g., GLUT1, HK2, and Pdk1) in mouse sarcoma cells has been shown to inhibit the effector function of CD8+ T cells [214].

#### OXPHOS in T cells

The regulation of OXPHOS is as important as aerobic glycolysis in activating T cells, and the induction of mitochondrial ROS is necessary for antigen-specific T cell responses in vivo [238]. Loss of ATP production during OXPHOS restricts T cell proliferation and effector functions. In contrast, the maintenance of redox homeostasis promotes T cell self-renewal and enhances anti-tumor immunity [271]. IL-17-producing TH17 cells are mediators of body defense, autoimmunity, and antitumor immunity and depend on OXPHOS to exert their effects in vivo, exhibiting an antiapoptotic phenotype characterized by enhanced mitochondrial function [272]. Immune cells in TME enhance E2F-PPARδ activity in CD8+ T cells through autocrine and paracrine secretion of METRNL, leading to mitochondrial depolarization and reduced OXPHOS, ultimately causing energy depletion in CD8+ T cells and a diminished antitumor response [273]. Mitochondrial remodeling is a signaling mechanism that directs the metabolic programming of T cells, and mitochondrial fusion in memory T cells configures electron transport chain (ETC) complex association in favor of OXPHOS and FAO. In contrast, fission of mitochondria occurs in effector T cells to promote aerobic glycolysis [274]. Upon activation of the TCR, the mitochondrial OXPHOS of T cells and NADPH oxidases (NOXs) are activated, which can lead to increased ROS production [275]. Although uncontrolled accumulation of ROS can be cytotoxic, physiological levels of mitochondrial ROS(mROS) are essential for T cell activation. mROS facilitate the activation of NFAT, which in turn contributes to the production of IL-2 [238]. ROS can also upregulate the aromatoid receptor (AhR) to promote the transition of effector T cells to memory T cells [275]. In addition to this, tumors affect the function of mitochondria in T cells. T cells from cancer patients showed signs of decreased mitochondrial mass and mitochondrial dysfunction compared to healthy controls tumor-infiltrated CD8+ T cells compared to non-infiltrated CD8+ T cells in tumor-loaded mice [276, 277].

#### Anti-tumor lipid metabolism in T cells

Fatty acid-related metabolism is closely related to T cell activation, differentiation, and function. Regarding fatty acid acquisition, Tregs are more dependent on extracellular fatty acid uptake [278], whereas Th17 cells are more inclined to synthesize fatty acids from scratch [279]. Extracellular fatty acids of different carbon chain lengths also differentially regulate T cell subtypes. Short-chain fatty acids (SCFAs), mostly fermented from dietary fiber by gut microbiota, enhance the effector function of CD8+ cells [280] and facilitate the transition of activated CD8+ T cells to long-term survival memory cells [281]. In contrast, long-chain fatty acids (LCFAs) enhance the differentiation and proliferation of Th1 and Th17 cells [282]. T-cell activation upregulates FAS via the mTOR-SREBP pathway, which is significantly associated with effector T-cell differentiation and proliferation [283]. FAS is associated with acetyl-Coenzyme A carboxylase (ACC) and ATP-citrate lyase (ACLY), which control the production of precursors required for lipogenesis. TH17 cell development depends on ACC1-mediated fatty acid de novo synthesis and the glycolytic-lipogenic metabolic pathway [279]. Inhibition of the ACC1 gene promotes the formation of CD4+ T memory cells [284]. ACC1-mediated fatty acid ab initio synthesis is important as a regulator of CD8+ T cell expansion [285]. However, recent studies have shown that ACC directs LDL accumulation in CD8+ T cells, and inhibition of ACC in CD8+ T cells promotes FAO and enhances its persistence and tumor suppression [286]. T cells can also be supplied with an energy source via FAO, and preferential use of FAO has been linked to T-cell differentiation and function.CD8+ memory T cell formation is dependent on the upregulation of FAO, and AMPK can be activated to promote FAO [287, 288]. Lipid droplets, which are sites of intracellular lipid storage, are less abundant in effector CD4+ T cells than in memory CD4+ T cells and can be utilized by cells in nutrient-poor environments [289].

SREBP plays an important role in cell membrane synthesis in T cells, and CD8+ T cells lacking SREBP1 and SREBP2 have significantly diminished proliferative capacity. This proliferative defect has been traced back to cholesterol insufficiency [290]. SREBPs promote lipid synthesis and require activation of SREBP cleavage-activating protein (SCAP). In turn, SCAP-SREBP signaling drives the reprogramming of lipid metabolism and can promote T cell exit from quiescence and cell proliferation [290]. A key regulator of steroid transport and synthesis is the LXR, which comes in two forms: LXRα and LXRβ. Lack of LXRβ increases the proliferation of CD4+ and CD8+ T cells, whereas CD8+ T cells deficient in LXRβ produce more effector cytokines (e.g., IFN-γ) in the secondary immune response [291]. The mevalonate pathway is essential for cholesterol anabolism, with HMG-CoA reductase as the rate-limiting enzyme. Upon T-cell activation, the mevalonate metabolic pathway is up-regulated to meet the demands of cell proliferation and biosynthesis [292].

In glycerophospholipid metabolism, TCR with co-stimulatory signals and IL-2 can activate PI3K, which converts phosphatidylinositol diphosphate (PIP2) to phosphatidylinositol triphosphate (PIP3), which then promotes T-cell proliferation and anti-tumor effects through the PI3K-Akt pathway [293]. TCR activation also promotes phospholipase C (PLC). Hydrolysis of PIP2 generates inositol trisphosphate (IP3) and diacylglycerol (DAG) as second messengers. IP3 can mobilize intracellular calcium ions to be released into the cytoplasm [294], and DAG promotes the activation of protein kinase C (PKC), which activates NFAT and transcription factors such as NF-κB, which are essential for T-cell proliferation activation, etc. Diacylglycerol kinases (DGKs) are lipid kinases that convert DAG to phosphatidic acid, and inhibition of DGKα enhances the antitumor effects of effector T cells [295]. Sphingolipids are major components of cell membranes, and their metabolites, such as ceramide, sphingosine, and sphingosine-1-phosphate (S1P), are biologically active signaling molecules. Ceramide, formed by dehydration of a fatty acid with the amino group of sphingosine, is a lipid overload signal that helps cells process fatty acids from excessive intake [296]. It has been shown that injection of C6-ceramide-containing nanoliposomes into mice with hepatocellular carcinoma enhanced antigen-specific CD8+ T cell function and delayed tumor growth [297].

#### Tumor immunosuppressive lipid metabolism in T cells

SCFAs can promote the differentiation of CD4 + T cells to Tregs and play an immunosuppressive role [298, 299]. In contrast, the accumulation of LCFAs in pancreatic ductal carcinoma will lead to CD8+ T cell dysfunction [300]. CD36 is a transmembrane glycoprotein and scavenger receptor that transports LCFAs and oxidized low-density lipoproteins (OxLDL). CD36 is selectively up-regulated in tumor Tregs, regulates mitochondrial function through PPARβ signaling, and adapts Tregs to the lactate-rich TME, suppressing tumor immunity [301]. CD36 can mediate the uptake of OxLDL by CD8+ T cells to promote lipid peroxidation or induce iron death in CD8+ T cells, thus inhibiting their antitumor effects [302, 303]. Recently, it has been shown that through CD36 signaling, OxLDL and palmitate can synergistically inhibit T cell activity in AML cells [304]. Peroxisome proliferator-activated receptors (PPARs) are transcription factors involved in regulating a variety of genes related to cell metabolism, differentiation, and function, and FA is their ligand [305]. PPARγ can inhibit TGF-β/IL-6-induced Th17 differentiation in CD4+ T cells [306], and it is also an essential driver of Treg cell differentiation, especially in visceral adipose tissue (VAT) [307]. Concerning FAS, inhibition of ACC1 suppresses human and mouse TH17 cell formation and promotes Treg cell development [279]. ACLY is actively degraded during iTreg cell differentiation, leading to a down-regulation of FA and a corresponding up-regulation of FAO to support iTreg proliferation [308]. In addition to this, Tregs are largely dependent on FAO to maintain their inhibitory function and stability [309]. CPT1 is involved in the transport of lipoyl CoA into the mitochondria, and studies have shown that butyrate, a SCFA, can enhance CPT1A activity to promote FAO and iTreg differentiation [310]. Tregs have a higher content of lipid droplets that can be utilized more in the TME than other T cells [311]. PG is synthesized from arachidonic acid in vivo. In the TME, high expression of PGE2 by tumor cells promotes the differentiation of initial T cells into Tregs and enhances tumor immunosuppression [312]. PGE2 also restricts the expansion and antitumor effects of tumor-infiltrating stem cell-like CD8+ T cells [313].

During activation and proliferation of CD8+ T cells, membrane cholesterol content is partially regulated by acyl-coenzyme A cholesterol acyltransferase 1 (ACAT1), which esterifies cholesterol. ACAT1 knockdown CD8+ T cells exhibit increased membrane cholesterol and enhanced TCR aggregation and signaling, which improves T-cell proliferation, function, and tumor-killing capacity [314]. Oxidized sterols (cholesterol oxidation products) produced in the TME lead to alterations in two transcription factors related to cholesterol metabolism (SREBP2 and LXR), which in Treg depletion and dysfunction [315]. High cholesterol levels in the TME may also lead to T-cell depletion and dysfunction by activating the ER stress response [316, 317].

The phosphatase PTEN can inhibit T cell proliferation and differentiation by inhibiting the PI3K-AKT pathway [318]. In lung cancer, the expression of phospholipid phosphatase 1 (PLPP1), which catalyzes the synthesis of phosphatidylcholine (PC) and phosphatidylethanolamine (PE), is reduced in CD8+ T cells within tumors. In contrast, T-cell-specific deletion of Plpp1 impairs antitumor effects and promotes T-cell iron death [319]. Cardiolipin, a substance synthesized in the inner mitochondrial membrane, maintains CD8+ T cell function. PTPMT1 is an enzyme necessary for cardiolipin synthesis. T cells deficient in PTPMT1 have a weaker response to antigenic stimulation and impaired mitochondrial function, which restricts differentiation to memory T cells [320]. Lysophosphatidic acid (LPA) is a biologically active lipid that increases in concentration both locally and systemically in different cancer types, and it has been shown that LPA receptor 5 (Lpar5) signaling in CD8+ T cells promotes a cellular state associated with a CD8+ T cell exhaustion phenotype, thereby suppressing tumor immunity [321]. Sphingosine can regulate c-Fos, a direct target of Tregs, which affects the expression of target genes FoxP3 and PD-1 downstream of the transcription factor c-Fos, directly promoting Treg cell differentiation and suppressing tumor immunity [322]. Tumor cells and Tregs in the TME can promote the expression of phospholipase A2-IVA, which induces alterations in lipid metabolism of T cells and senescence, ultimately leading to tumor immune escape [323].

#### Amino acid metabolism in T cells

Activated T cells depend on amino acid metabolism to support protein and nucleotide synthesis. Solute carriers (SLCs), which are responsible for transporting several substances across the cell membrane, are also closely linked to amino acid transport. Amino acids may influence T cell-related functions through the mTOR pathway and the general regulation of the GCN2 pathway, as well as through amino acid metabolic products. mTOR primarily exists in two cell complexes, mTORC1 and mTORC2, which are involved in cell proliferation, metabolism, and protein synthesis. GCN2 senses amino acid deficiency by binding to uncharged tRNAs and activates the phosphorylation of eukaryotic initiation factor 2 (eIF2α) to regulate T cell metabolism [324].

Both tumor cells and activated T cells usually demand glutamine. Glutamine can be taken up via the amino acid transporter ASCT2 (SLC1A5), critical for CD4+ T cell activation and differentiation [325]. Glutamine transporters SLC38A2 and SLC7A1 regulate the mTORC1 signaling pathway, limiting the generation and persistence of CD8+ memory T cells [326]. Glutamine is also a precursor of UDP-GlcNAc, which is involved in regulating T cell self-renewal through the promotion of protein O-GlcNAc glycosylation (O-GlcNAcylation) [259]. Glutamine can drive the TAC cycle via the anaplerotic reaction, where first, GLS metabolizes glutamine to glutamate, which is converted to αKG by glutamate dehydrogenase (GDH) or transaminase and subsequently enters the TCA cycle [327]. After the metabolic rearrangement, many tumor cells consume additional glutamine to produce other substances to satisfy the TCA cycle and proliferate indefinitely, a condition known as “glutamine addiction” [328]. Therefore, glutamine restriction may have a more direct effect on tumor cell survival than on T cells, and blocking glutamine metabolism relatively enhances the antitumor effects of T cells [329].

Branched-chain amino acids include valine, leucine, and isoleucine. BCAAs are taken up by the cell via L-type amino acid transporter proteins (LATs) and then transported from the cytoplasm to the mitochondria via SLC25A44 [330], where leucine uptake is accomplished by LAT1 (SLC7A5) [331]. Leucine regulates the mTORC1 signaling pathway and affects the anti-tumor immune function of CD8+ T cells through RagD-mediated activation of mTORC1. Leucine deficiency significantly inhibits the activity and proliferation of CD8+ T cells [332]. Activation of the TCR in CD4+ T cells increases the expression of BCAT1 (cytosolic branched-chain aminotransferase) and SLC7A5, enhancing leucine uptake and catabolism, and promotes Th17 cell differentiation and function [333].

Due to the low levels of Trp in the TME, tumor cells compete with Teff cells for Trp to inhibit their function. Colorectal cancer cells compete with CD8+ T cells for Trp, impairing CD8+ T cell function. Metformin restores CD8+ T cell function and enhances cytotoxicity by reducing Trp uptake by colorectal cancer cells [334]. Trp depletion in vitro using IDO+ DCs induced proliferative arrest and dysfunction of T cells, whereas this effect disappeared in GCN2 knockout T cells [335]. Besides, Trp can enhance the toxicity of CD8+ T cells against cancer cells through the Trpylation of TRIP12, while down-regulating PD-1 expression and enhancing anti-cancer immune responses [336]. Trp can be catabolized to kynurenine (Kyn) by indoleamine-2,3-dioxygenase (IDO) or Trp-2,3-dioxygenase (TDO2). Kyn in the TME has effects on both tumor cells and T cells. IDO-1 is overexpressed in multiple tumor types and can be used for immune escape by increasing Trp catabolism and inhibiting T cell activation [337]. Recent studies have shown that stromal fibroblasts with high TDO2 expression in triple-negative breast cancer(TNBC) can suppress tumor cell ferroptosis and the antitumor activity of Teff cells, while promoting Treg differentiation. This occurs through the catabolism of Trp into Kyn, which is subsequently secreted into the extracellular matrix [338]. The ratio of Trp to Kyn can reflect the therapeutic effect of anti-PD-1 monoclonal antibodies, with lower ratios resulting in stronger anti-tumor responses [339]. Kyn also binds to the aromatic hydrocarbon receptor (AHR) on T cells, upregulates PD-1 expression in CD8+ T cells, and promotes Treg cell differentiation [340].

L-Arg regulates T cell metabolism to promote T cell survival and antitumor activity [341]. Arg uptake can be controlled by the CAT family of SLC7A transporters [342]. Increased serum Arg reduces the immunosuppressive capacity of Tregs via the mTOR pathway [343]. In contrast, low Arg-activated Tregs utilize the ATF4-SLC7A11-GSH axis for metabolic and transcriptional reprogramming to maintain suppressive function [344]. Arg is mainly metabolized by ARG-1, arginase-2 (ARG-2) and nitric oxide synthase (NOS). Fetal DCs can strongly promote Treg cell induction and inhibit T cell TNF-α production by ARG-2 activity [345]. Inhibition of NOS attenuates the maintenance effect of Arg for CD4+ T cells, revealing the importance of NO production catalyzed through NOS for T cell proliferation [346]. Protein Arg methyltransferase 5 (PRMT5), an epigenetic modifier that catalyzes the symmetric dimethylation of Arg residues on histones and non-histone proteins, regulates a variety of cellular processes and is involved in cancer formation and progression [347]. High expression of PRMT5 in tumors is associated with poor prognosis in patients with cervical cancer, whereas PRMT5 deficiency leads to an increased number of tumor-infiltrating T cells and enhances their function [348].

Methionine plays a key role as a methyl group donor in T cells. In this process, methionine is converted to S-adenosylmethionine (SAM) via methionine adenosyltransferase (MAT) catalysis. It is involved in various biosynthetic and metabolic pathways, such as methylation reactions, maintenance of redox homeostasis, and polyamine synthesis [349]. Depletion of methionine by tumor cells leads to up-regulation of PD-1 expression in CD4+ T cells, impairing their anti-tumor capacity [350]. SLC7A5 is considered to be the most abundant methionine transporter in activated T cells [351]. Meanwhile, tumor cells disrupt CD8+ T-cell methionine metabolism through high expression of the transporter SLC43A2 to consume large amounts of methionine in the TME, which reduces methionine and SAM levels, leading to loss of histone H3 (H3K79me2) dimethylation, low STAT5 expression, and impaired T-cell immunity [352]. Methylthioadenosine phosphorylase (MTAP) is a key enzyme in the remediation pathway for purine and methionine synthesis. MTAP deficiency leads to intra- and extracellular accumulation of MTA, which, in turn, severely impairs T-cell function by inhibiting PRMT5 and adenosine receptor signaling. Administration of MTAP scavenging enzyme reverses this immunosuppressive effect, increases TILs, and effectively suppresses tumor growth [353].

**Fig. 5:**
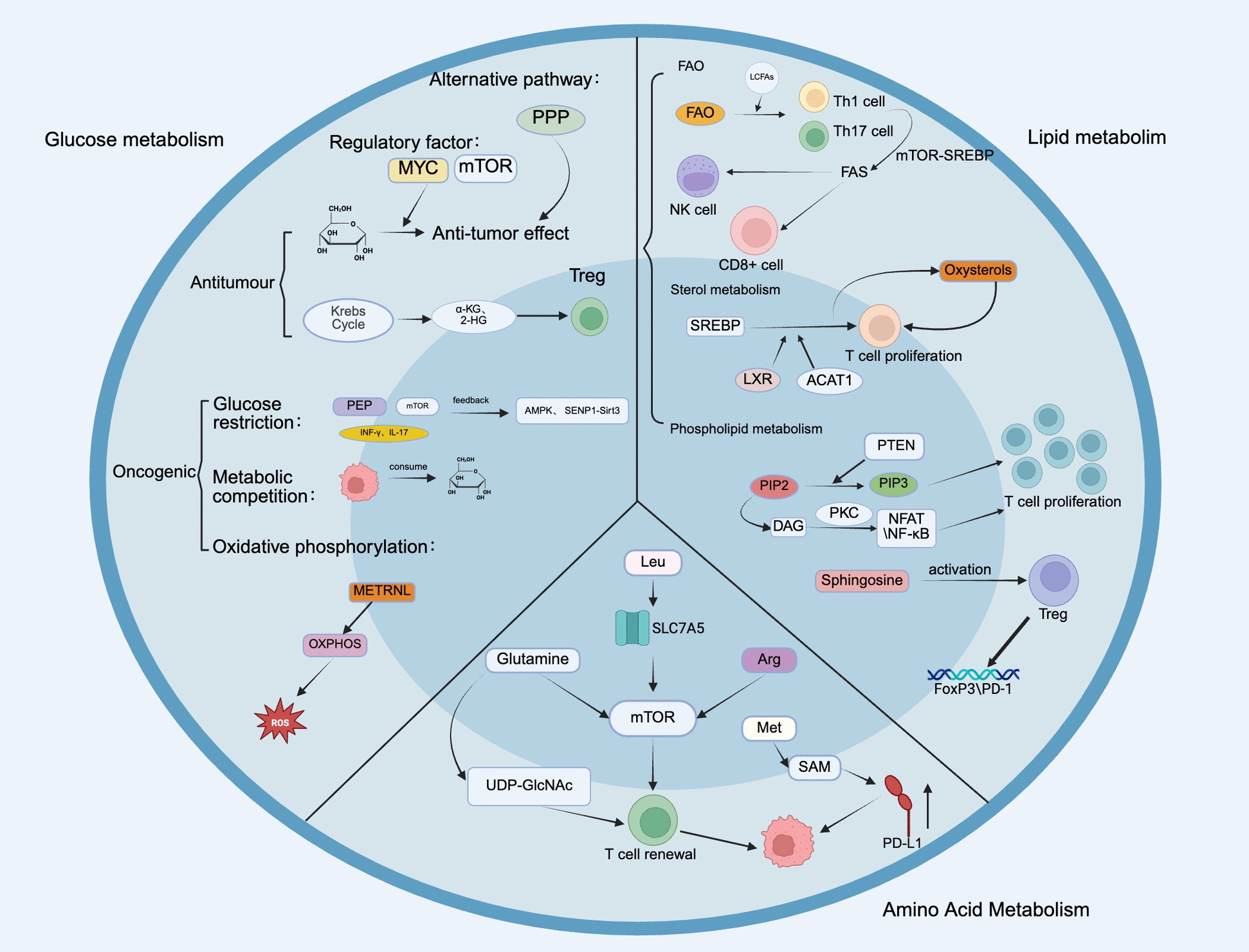
The three primary metabolic pathways of T cells and their contributions to anti-tumor and pro-tumor effects. Normal glucose metabolism is strongly linked to T cell-mediated anti-tumor activity. However, metabolic feedback, particularly concerning pathways related to phosphoenolpyruvate (PEP), can restrict glucose availability. Additionally, tumor cells within the tumor microenvironment (TME) compete with T cells for glucose, while the accumulation of reactive oxygen species (ROS) further facilitates tumor metastasis. In terms of amino acid metabolism, the mTOR pathway plays a crucial role in regulating T cell renewal and maintaining their anti-tumor functions. For lipid metabolism, fatty acid metabolism is essential for the activation of anti-tumor T cells, cholesterol metabolism meets the requirements for T cell proliferation, and phospholipid metabolism enhances T regulatory cell (Treg) function, potentially inhibiting overall T cell proliferation. Created with BioRender.com; *Th1 cell* T—helper 1 cell, *Th17 cell* T—helper 17 cell, *FAS* Fatty Acid Synthase, *CD8 + cell* Cluster of Differentiation 8—positive cell, *PTEN* Phosphatase and Tensin Homolog, *PIP2* Phosphatidylinositol 4,5—bisphosphate, *PIP3* Phosphatidylinositol 3,4,5—trisphosphate, *DAG* Diacylglycerol, *PKC* Protein Kinase C, *NFAT* Nuclear Factor of Activated T—cells; *NF—κB* Nuclear Factor—kappa B, *Leu* Leucine, *Arg* Arginine, *Met* Methionine

Serine, an important one-carbon unit donor to the folate cycle, is involved in nucleotide synthesis, methylation reactions, NADPH production, and supports protein and glutathione (GSH) synthesis [354]. When T cells are activated, they enhance the activity of enzymes associated with serine, glycine, and one-carbon (SGOC) metabolic pathways and accelerate the utilization of serine in one-carbon metabolism [261]. Extracellular serine is essential for the optimal proliferation of effector T cells even when glucose levels are sufficient to support T cell activation, energy production, and function [261]. Serine is converted to GSH by glutamate cysteine ligase (Gclc); deficiency of GSH disrupts the suppressive capacity of Tregs, thereby enhancing anti-tumor immune responses [355]. (Fig. 5).

### B-cells

#### Introduction

Although relatively few studies have been conducted on the relationship between B cell metabolism and antitumor effects in the TME, B cells are important in adaptive antitumor immunity. B cells, which include antigen-presenting B cells, plasma cells, and memory B cells, exert their effects by recognizing and presenting antigens, secreting cytokines, and producing antitumor-specific antibodies [356]. B cells are also associated with tertiary lymphoid structures (TLS). B cell clones are selectively activated and expanded in the germinal centers of the mature TLS to further differentiate into plasma cells that produce antibodies targeting tumor-associated antigens [357]. Antibodies produced by B cells can kill tumor cells through mechanisms such as antibody-dependent cytotoxicity (ADCC), antibody-dependent cell phagocytosis (ADCP), and complement-dependent cytotoxicity (CDC) [358]. Regulatory B cells (Breg), on the other hand, can inhibit immune responses together with Tregs [359].

#### Glucose metabolism in B cells

Similar to T cells, B cells showed increased glucose uptake and lactate production upon stimulation, particularly enhanced glycolytic activity in B cells of the germinal center [360]. Although B cells enhance glucose uptake, isotope tracing revealed that the increase in TAC cycle intermediates had little contribution from glucose. That inhibition of glucose did not affect B cell function [361].

#### OXPHOS in B cells

The OXPHOS pathway plays a key role in the positive screening of B-cell affinity in the germinal center [362]. Inhibition of OXPHOS impairs B cell growth and differentiation [361]. However, it has been shown that activated B cells can deprive T cells of oxygen and glucose by enhancing OXPHO and glycolytic activity, inhibiting the mTOR pathway and cytokine production in T cells [363]. The metabolite of ATP, adenosine (ADO), is an immune-suppressing molecule. Breg cells can inhibit T cells through the production of ADO function [364].

#### Lipid metabolism in B cells

SCFAs produced by gut microbiota activate B-cell metabolic processes, contribute to producing ATP, acetyl-CoA, and lipids, and promote plasma cell differentiation [365]. In addition, SCFAs promote immunoglobulin production and upregulate the expression of genes associated with antibody production, plasma B cell differentiation, and immunoglobulin class switch recombination (CSR) [365]. Stearoyl-CoA desaturase (SCD) converts saturated fatty acids to monounsaturated fatty acids (MUFA) to maintain B cell mitochondrial metabolism and mTOR activity [366].

#### Amino acid metabolism in B cells

Glutamate is the precursor of gamma-aminobutyric acid (GABA), an inhibitory neurotransmitter. Activated B cells and plasma cells can secrete GABA, which promotes the differentiation of monocytes into anti-inflammatory macrophages, which secrete IL-10 and inhibit the antitumor effects of CD8+ T cells. Atypical memory (AtM) B cells are the main source of antibody-secreting cells ASCs produced by the extrafollicular (EF) pathway and develop independently of the GC pathway. The metabolite of glutamine, α-ketoglutarate (α-KG), elevates T-bet and BATF expression in AtM B cells, promotes their differentiation, and activates mTORC1 signaling, which in turn provides AtM B cells with immunomodulatory functions and suppresses anti-tumor T cell responses. In addition, EF B-cell responses have been associated with immunosuppressive TME and poor prognosis, while GC B-cell responses have been associated with tumor immunity and good outcomes [367]. It has been shown that in colorectal cancer, there is a subpopulation of leucine-tRNA synthetase-2 (LARS2)-expressing B cells that elevate TGF-β1 expression in the presence of leucine induction, leading to immune escape [368]. The receptor substrate hydrocarbon receptor (AhR) for kynurenine promotes the differentiation of IL-10-secreting Breg cells for immunosuppression [364]. Adaptive immune cells often play a more pivotal role in antitumor immunity, particularly T cells. The metabolic mechanisms of T cells within the TME have been discussed above; however, the metabolism of B cells and their roles in antitumor immune responses remain insufficiently understood and warrant further investigation. (Table 6).

**Table 6:** Metabolic targets related to adaptive immune cells

Cell	Effect	Metabolism type	Target	Mechanism	Refs.
T cell	Antitumor	Glucose metabolism	MYC	Exerting antitumor functions	[242]
			HIF-1α	Regulating glycolysis and CD4+/CD8+ T cell function via the mTORC1-HIF-1α axis	[242–244]
			GLUT1	Activating the PI3K/Akt pathway maintains the anti-apoptotic ability of CD8+ T cells	[249]
			GLUT2	Regulating the effector response of CD8+ T cells	[251]
			Tcf7	Enhancing the stemness of CD8+ T cells	[252]
			G6PD	Promoting CD8 + T cell production	[256]
		OXPHOS	AhR	Promoting the transition from effector T cells to memory T cells	[275]
		TCA cycle	α-KG	Inhibiting the differentiation of FoxP3 + Tregs	[262]
			2-HG	Promotes the differentiation of TH17 cells and inhibits the differentiation of iTregs	[263]
		lipid metabolism	ACC1	Enhancing the persistence and tumor-suppressing ability of CD8+ T cells	[284]
			SREBP1/SREBP2	Promoting T cells to exit from quiescence and undergo cell proliferation	[290]
			LXRβ	LXRβ-deficient CD8+ T cells produce more effector cytokines (e.g. IFN-γ) in the secondary immune response	[291]
			TCR	Promoting T cell proliferation and antitumor effects	[293]
			DGKs	Inhibiting DGKα can enhance the antitumor effects of effector T cells	[295]
		Amino acid metabolism	SLC1A5	SLC1A5 is essential for the activation and differentiation of CD4+ T cells	[325]
			SLC7A5	Promoting the anti-tumor immune function of CD8+ T cells	[331]
			BCAT1	Promoting the differentiation and function of Th17 cells	[333]
			SLC7A1	Reducing the immunosuppressive capacity of Tregs via the mTOR pathway	[342]
			MTAP	Promoting T cell function	[353]
			Gclc	Enhancing anti-tumor immune response	[355]
	Oncogenic	Glucose metabolism	PEP	Reducing antitumor effects	[266]
			Pdk1	Enhancing the glycolytic capacity of tumor cells and inhibiting the effector function of CD8+ T cells	[214]
		OXPHOS	METRNL	Reducing antitumor effects	[273]
		Lipid metabolism	CD36	Regulation of mitochondrial function through PPARβ signaling adapts Tregs to lactate-rich TME and suppresses tumor immunity	[301]
			ACC1	Inhibition of ACC1 suppresses human and promoting Treg cell development	[286]
			ACLY	Supporting iTreg proliferation	[308]
			CPT1	Enhancing CPT1A activity to promote FAO and iTreg differentiation	[310]
			PGE2	Limiting the expansion and antitumor effects of tumor-infiltrating stem cell-like CD8+ T cells	[313]
			ACAT1	Inhibiting the function of CD8+ T cells	[314]
			PTEN	Inhibition of the PI3K-AKT pathway and thus T cell proliferation and differentiation	[318]
			PLPP1	T cell-specific deletion of Plpp1 impairs antitumor effects and promotes T cell iron death	[319]
			PTPMT1	Limiting differentiation to memory T cells	[320]
			Lpar5	Correlating with CD8+ T cell depletion phenotype and suppresses tumor immunity	[321]
			A2-IVA	Leading to tumor immune escape	[323]
		Amino acid metabolism	SLC38A2/SLC67A1	Limiting the generation and persistence of CD8+ memory T cells	[259]
			PRMT5	An epigenetic modifier that catalyzes symmetric dimethylation of Arg residues involved in cancer formation and progression	[347]
			SLC43A2	Disrupting CD8+ T cell methionine metabolism	[352]
B cell	Antitumor	OXPHOS	ADO	Breg cells can suppress T cell function by producing ADOs	[364]

## Immunosuppressive cells

### Regulatory T cells (Tregs)

#### Introduction

Tregs, as immunomodulatory cells, are a subpopulation of CD4+ T cells, and their high expression of CD25 and forkhead transcription factor 3 (FoxP3) molecules plays a role in suppressing the immune response. Tregs have a significant anti-inflammatory effect, and they can secrete anti-inflammatory cytokines such as IL-4, IL-10, and TGF-β to suppress auto-inflammatory responses [369]. The metabolism of Tregs in the TME is very important for their proliferation, migration, and immunosuppressive function [370]. Many studies have proved that the accumulation of Tregs is negatively correlated with tumor prognosis [371, 372], and its main mechanisms of action are: (1)secretion of inhibitory cytokines, such as IL-4, IL-10, and TGF-β, which suppress the auto-inflammatory response; (2)cytolysis, whereby Tregs kill effector cells through granzyme and perforin; (3)receptor-ligand interactions, such as CTLA-4 binds to B7 and competes to inhibit the CDS; (4)promotion of other immunosuppressive cell types, facilitating their differentiation and function within the TME. Tregs induce monocytes to differentiate into the M2 subtype of TAMs, thereby suppressing antitumor immunity, while promoting the differentiation and function of myeloid-derived suppressor cells (MDSCs) by secreting TGF-β [373–376]. Like other cells in the TME, Tregs are subjected to metabolic reprogramming by various stimuli, and their effects are important for their proliferation, differentiation, and function. In this subsection, we will explore the metabolic characteristics of Tregs in the TME and their effects on tumor cells.

#### Glucose metabolism and OXPHOS

Tregs'proliferation mainly depends on the upregulation of glycolysis. The targets of key enzymes of glycolysis are related to the target of mTOR signaling pathway [318, 377], which usually mediates the upregulation of T-cell glycolysis. In vitro experiments proved that the upregulation of glycolysis, as well as the induction of overexpression of GLUT-1, significantly upregulated their proliferative capacity, but their immune suppressive function was severely blocked. Therefore, to exert its immunosuppressive effect, glycolysis should be tightly regulated in Tregs [378, 379]. The migration of Tregs to the tumor site is also dependent on the glucose fermentation process, and the activation of PICOP by the surface molecule CD28 is stimulated. The migration of Tregs to the tumor site is also dependent on the process of glucose fermentation and is determined by the activation of glucokinase induced by the surface molecule CD28 upon stimulation of the PI3K-mTORC2 signaling pathway, the hexokinase isoform glucokinase (GCK) is a key element of this process, which mTOR also regulates [380–382]. Studies have shown that mTOR activity is reduced in Tregs, mainly dependent on elevated serine-threonine phosphatase (PP2A) activity. Foxp3 directly inhibits the expression of phospholipase synthase 1 (SGMS1), which leads to the accumulation of intracellular ceramides, which in turn inhibits the phosphorylation of inhibitory amino acids on PP2A, resulting in a reduction of mTOR activity [383].

One of the unique metabolic characteristics of Tregs is that they possess stronger mitochondrial metabolism than Tconvs [384]. The energy source of the immunosuppressive function of Tregs relies mainly on OXPHOS in the mitochondria. It has been demonstrated that, when mitochondrial complex III is inhibited, the number and proliferation rate of Tregs are appear normal. Still, the inhibitory function is affected in vitro and in vivo, demonstrating the functional segmentation of glycolysis and OXPHOS in Tregs [385]. It has been shown that the occurrence and alteration of the above metabolic pathways are significantly linked to Foxp3 [239], and that mitochondrial metabolism is mainly dependent on the reprogramming of metabolism by the FoxP3 transcription factor, which inhibits signaling by Myc, thereby suppressing glycolysis and glutamine catabolism while promoting mitochondrial respiration. This adaptation allows Tregs to function in the low-glucose, high-lactate conditions of the TME, enhancing their immunosuppressive activity [239, 374]. These findings have been demonstrated in small cell lung cancer, glioblastoma, and bladder cancer, and are considered promising therapeutic targets [386–388].

In the TME, the nutritionally deficient environment is less likely to inhibit the function of Tregs. Still, it may also enhance their metabolic adaptations, such as glucose or glutamine deprivation, accumulation of lactate, kynurenine, and polyamines [95]. Among them, lactate is more critical for Tregs. It activates the G protein-coupled receptor (GPR81) [177] and enters the cell via the monocarboxylic acid transporter protein (MCT1), which is metabolized by Tregs through a series of metabolisms that feed lactate into the mitochondria and allow it to enter the TCA cycle. It was further shown that Tregs incorporate lactate-derived carbon into the metabolism of PEP, which contributes to upstream glycolytic intermediates necessary for proliferation, suggesting that lactate can serve as a glycoheterotrophic fuel source that reduces glucose demand by Tregs [389]. Moreover, lactate can affect Treg function through non-metabolic pathways. Reports have shown that lactate entry induces lactylation of the membrane-to-cytoskeleton attachment protein at Lys72, thereby promoting Treg cell development and function by modulating TGF-β signaling via TGF-βRI [77]. Low pH in TME is often a marker for a poor prognosis of the patient. Several studies have demonstrated that acidity is a key driver of cancer [390–392], so a team also investigated whether lactate alone as an acid promotes Treg cell differentiation and development. The results showed that the effect of lactate on the transformation of Tregs into FoxP3 cells was and is pH-dependent [393]. This study provides an indication of the role of pH rather than lactate in mediating the effect of lactate on Treg cell differentiation and development.

#### Lipid metabolism

Numerous studies have demonstrated that the immunosuppressive properties displayed by Tregs at the tumor site are very closely linked to lipid metabolism, which is largely dependent. Although Teff cells in the TME can also upregulate lipid metabolism to enhance anti-tumor immune effects, the high lipid content in the TME can promote immunosuppression, as most immune cells are less efficient than Tregs in utilizing lipids [394].

It has been experimentally demonstrated that SCFA inhibits the efficacy of anti-CTLA-4 therapy, leading to an upregulation of Tregs. However, recent studies have shown that maternal supplementation of intestinal SCFA acetate could reverse this trend when abnormal secretion was observed in thymus-damaged Tregs in the offspring. These findings suggest that SCFA-mediated regulation of Treg cell differentiation and immunosuppression is both potent and context-dependent [395, 396], This effect may be primarily mediated by the induction of IL-10, FOXP3 transcription via the JNK1 and p38 pathways, thereby promoting Treg cell expansion [282, 397]. However, there is still a lack of research progress on the effects of LCFAs as a major component of the human diet on Tregs. Currently, some studies have indicated that free fatty acid receptor 4 (FFAR4), a receptor for LCFAs, exacerbates inflammatory bowel disease (IBD) by decreasing the number of Tregs in IBD when the expression of LCFAs is higher [398], which may indirectly demonstrate that LCFAs inhibit the Treg cell effect, as mentioned in other articles [399]. In the gut, LCFAs can induce differentiation and proliferation of Th1 and/or Th17 cells, which may lead to an imbalance of Th17/Tregs [282]. The lipid receptor peroxisome PPARγ has been implicated as a key component of FAO in previous studies, causing up-regulation of CD36 expression and promoting lipid transport, which in turn up-regulates FAO [394], as evidenced by the fact that PPARγ activation enhances the Treg cell response by up-regulating CD36/CPT1-mediated FAO and the subsequent N-glycan branching of TβRII/IL-2Rα [400]. Alongside the Treg cell response, the fatty acid transport protein SLC27A1 is also significantly upregulated [401]. Recently, it has been reported that Tregs in IBD prefer to utilize unsaturated fatty acids (PUFA) to synthesize membrane phospholipids. This habit makes them vulnerable Tregs to ROS-induced phospholipid peroxidation. Compared to Tconv, Tregs expressing higher levels of CD71 (transferrin receptor) generate more ROS, both of which can increase the likelihood of a Fenton reaction that leads to lipid peroxidation and iron death [402]. This mechanism is expected to be further explored in Tregs infiltrating tumor tissue.

Also, cholesterol metabolism seems important for Tregs, with clinical data showing a positive correlation between serum HDL and the number of Tregs [403], and hypercholesterolemia increasing the number of Tregs in the liver [404]. These data suggest that cholesterol homeostasis is essential for Treg cell accumulation [405].

#### Amino acid metabolism

Amino acid metabolism also occupies an important position in Tregs. Amino acids in the TME (especially Trp & Arg) can license Treg cell function by triggering and maintaining TCR-induced mTORC1 activity via the small G proteins Rag and Rheb [406]. Recent studies have shed some light on the regulation of Treg cell function by branched-chain amino acids (BCAAs). Lipid accumulation in Tregs may inhibit BCAA catabolism by interfering with the metabolism of branched-chain ketoacid(BCKA). BCKA, in turn, have been shown to activate the mTORC1 signal pathway, although the underlying mechanisms remain to be fully elucidated [378, 407]. Glutamate enrichment in the TME can also directly increase the immunosuppressive function of Tregs. This effect has been observed during anti-VEGF therapy. Tumors often overexpress the glutamate/cystine reverse transporter protein SLC7A11/xCT, leading to the elevation of glutamate in the TME, which then promotes the overexpression and functional induction of Tregs, resulting in the loss of therapeutic efficacy. These findings indirectly demonstrate the importance of glutamate in the exertion of effects by Tregs [408]. Serine appears to similarly influence the inhibitory function of Tregs, being a source of glutathione (GSH) synthesis, which has been shown to have a novel role in limiting serine metabolism to support the inhibitory capacity of Tregs. However, the exact mechanism remains to be investigated [355]. Similarly, more studies have demonstrated that Tregs with high expression of arginase can deplete Arg from the TME in a nutrient-competitive manner, thereby preventing effector T cell function [378, 409]. Overexpression of indoleamine IDO-1 in the TME often leads to Trp deficiency and kynurenine production, thereby promoting the differentiation of Tregs. Interestingly, in a glioblastoma model, IDO-1 increases Treg cell recruitment and does not appear to be affected by Trp metabolism, suggesting that IDO1 can influence Treg cell development through enzymatic activity [410].

### MDSCs

#### Introduction

Myeloid cells have been discovered for over fifty years and were finally named MDSCs by scientists in 2007 [411]. According to previous studies, accumulation of MDSCs often occurs under pathological conditions of chronic inflammation, such as infections, tumors, etc. In tumors, it shows significant T-cell immunosuppressive effects, and its infiltration is closely associated with treatment resistance and poor prognosis [412]. Consequently, it is widely recognized as a critical contributor to tumor survival and progression [413–415]. MDSCs are essentially heterogeneous populations of myeloid cells, which are subdivided into two major groups of cells: polymorphonuclear MDSCs (PMN-MDSCs), with similar characteristics to neutrophils, and account for 80% of all MDSCs; monocytic MDSCs (M-MDSCs), which share features with monocytes. In addition, the immature myeloid-derived suppressor cell was recently proposed to be the early-stage MDSCs (e-MDSCs) [415]. The metabolism of MDSCs is significantly regulated in the TME, which is altered by metabolic reprogramming through glucose metabolism, lipid metabolism, and amino acid metabolism pathways [416]. The metabolic regulation of MDSCs and the relationship with immunosuppression will be explored below.

#### Glucose metabolism

The Warburg effect is also present in MDSCs, whose metabolism is reprogrammed by a combination of various cytokines and signaling pathways, including the PI3K-Ser-AKT-mTOR pathway, which is anabolic and imparts a high rate of glycolysis, even under aerobic conditions. The mTOR downstream factors, HIF-1α,c-Myc, and p53, which induce the expression of GLUTs, provide sufficient feedstock for high rates of glycolysis and activate the enzyme LDH, which converts pyruvate produced by glycolysis to lactate [414, 416]. Rapamycin removes this mTOR-mediated immunosuppression (reducing the number of MDSCs) [417, 418]. In contrast, AMPK, a vital nutrient energy sensor capable of regulating cellular energy homeostasis, can inhibit glycolysis by relying on the PI3K—AKT—mTOR pathway upstream of the same pathway as HIF-1α. During gluconeogenesis, AMPK drives glycolysis toward OXPHOS [414, 419].

During metabolism, the intermediate product phosphoenolpyruvic acid is generated, which has antioxidant properties and can prevent MDSCs from generating excessive ROS, the mechanism of which may be through the up-regulation of Nrf2. This transcription factor protects the cells from free radical damage, which in turn inhibits the production of ROS and thus prevents their apoptosis [413, 415, 420]. Meanwhile, lactate, a metabolite of glycolysis, as a key molecule in the TME, can directly contribute to immunosuppression. It inhibits immune cytotoxicity and proliferation and facilitates the differentiation and maturation of immunosuppressed cells. In the case of MDSCs, bone marrow-specific activation of the Notch/RBP-J signaling pathway has been reported to downregulate the transcription of the lactate transporter protein MCT2 through its downstream effector Hes1, thereby mediating the differentiation progression of MDSCs [421, 422]. The lactate transporter protein, monocarboxylate transporter (MCT), also plays an essential role in tumorigenesis. It has been reported that excess lactate produced or accumulated in MDSCs can be exported and subsequently taken up by oxidative cancer cells to fuel their energy metabolism, thereby promoting cancer cell activity [416, 423].

#### Amino acid metabolism

The metabolism of amino acids in MDSCs is also of great interest. They are protein-building molecules critical in tumor growth and developmental remodeling for stromal composition and vascular architecture in the TME [424]. Among the amino acids associated with promoting immunosuppression are mainly Arg, Trp, cysteine, and glutamine [414, 416], Their respective metabolic mechanisms and immunosuppressive effects will be discussed below.

Arg is the star amino acid, essential for T cells to generate activity and exert immune effects. Increasing the Arg catabolism in MDSCs can reduce the Arg content in TME and thus inhibit the activity of T effector cells. In MDSCs, it is metabolized mainly through two pathways: one pathway is catalyzed by Th2-induced overexpression of Arg1 to generate Orn and urea; the other pathway is catalyzed by Th1-induced overexpression of iNOS to generate NO and citrulline. Meanwhile, MDSCs can upregulate the expression of cationic amino acid transporter protein (CAT2), which increases the uptake of amino acids by cells in the TME, rendering the environment Arg-deficient. In the treatment of organ transplantation, rapamycin is often used as an immunosuppressive drug, targeting MDSCs cells, which enhances the expression of ARG-1 without enhancing the expression of iNOS. The therapeutic effect of rapamycin is eliminated when an ARG-1 inhibitor is used, implying the depletion of MDSCs. Also, experiments have been performed to verify that blocking CAT2 also has this effect, indirectly proving that Arg translocation and metabolism are important for MDSCs cells [425, 426].

The metabolism of Trp is also important in TME. Free Trp is metabolized mainly through the kynurenine pathway, where key enzymes involve IDO-1, IDO-2, and Trp-2,3-dioxygenase (TDO) [427]. Competitive depletion of Trp and binding of the downstream product kynurenine to the aryl hydrocarbon receptor can play a role in inducing immunomodulatory cell production and suppressing immune effects [428, 429]. Recent data support the key role of IDO-1 in immunomodulation. TDO and IDO-1 have similar roles, but IDO-2, as an isoform of IDO-1, also inhibits the differentiation and proliferation of effector T cells. However, fewer in-depth studies have been conducted, and there is no further data to elucidate this in detail [427, 430]. Notably, IDO-1 is anti-PD1 resistant, which suggests that its inhibition as a target is expected to improve drug resistance [431]. Recent studies have shown that IDO-1 also has a non-enzymatic function in tumors that can increase immunosuppression in a non-metabolic form, which is expected to introduce more in-depth studies [432].

Cysteine similarly inhibits the uptake of this conditionally essential amino acid by T cells from the environment using trophic competition, thereby acting to suppress the immune effect, and such a characterization of isolated cysteines has been mentioned in previous reviews [414, 416, 433].

#### Lipid metabolism

**Fig. 6:**
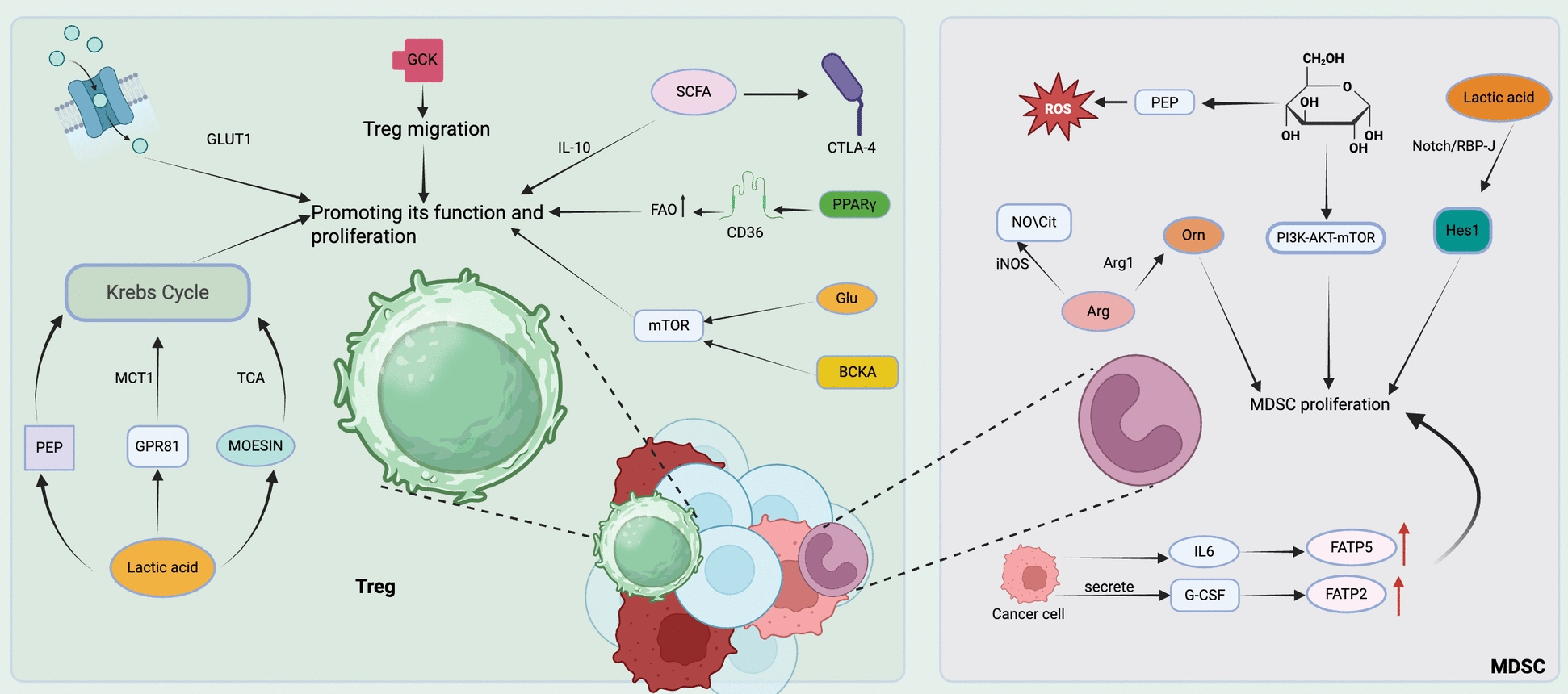
The metabolic processes of Tregs and MDSCs and their mechanisms in promoting immune escape. Lactate metabolism primarily promotes Treg function through the Krebs cycle, whereas amino acids exert their effects via the mTOR pathway. In MDSCs, it is noteworthy that tumor-derived factors such as IL-6 and G-CSF can also enhance MDSC proliferation. Created with BioRender.com

It has been shown that lipid metabolism in MDSCs indirectly blocks the effects of anti-tumor T cells and thus mediates immunosuppressive effects, mainly by increasing fatty acid uptake and up-regulating FAO. It has been demonstrated that tumor-infiltrated MDSCs (compared to normal splenic myeloid cells) express higher levels of lipid transport proteins, induced by tumor-derived cytokines (G-CSF and GM-CSF) and through STAT3 and STAT5 signaling. Interestingly, PMN-MDSCs (but not M-MDSCs) upregulated only FATP2, resulting in an abundant uptake of lipids in the TME, mainly free fatty acids, the triacylglycerol-carrying lipoproteins VLDL and LDL [146, 434]. Notably, the involvement of arachidonic acid and prostaglandins is essential during the maturation and development of PMN-MDSCs. IL6 upregulates fatty acid-binding protein 5 (FABP5) through signaling and phosphorylation of activator of transcription 3 (STAT3), and the increase in intracellular lipid accumulation mediates the metabolism of arachidonic acid, which further leads to increased PGE2 synthesis, and ultimately induces an increase in PMN-MDSCs'lipid uptake. The increase in intracellular lipid accumulation mediates arachidonic acid metabolism, which further leads to an increase in PGE2 synthesis, thereby inducing the differentiation of PMN-MDSCs [146, 435]. Some recent studies have shown that certain natural plant extracts, such as ginger polysaccharides, can promote apoptosis in MDSCs by modulating key lipid metabolizing enzymes, inhibiting FAS and lipid droplet accumulation, and reducing cellular energy supply [436]. Interestingly, an experimental group has recently found that triglyceride synthesis (as an alternative way to allow lipid accumulation in the cell as compared to ingestion) similarly favors the inhibitory function of MDSCs in cancer. The upregulation of diglyceride acyltransferase 1(DGAT1), a key enzyme involved in exogenous fatty acid uptake and triglyceride synthesis, has been linked to tumor progression. Notably, DGAT1 inhibition significantly impairs tumor growth in melanoma-B16F10 and Lewis lung carcinam-LLC models in C57/BL6 mice, highlighting its potential as a therapeutic target warranting further investigation (Fig. 6 and Table 7).

**Table 7:** Metabolic targets related to immunosuppressive cells

Cell	Effect	Metabolism type	Target	Mechanism	Refs.
Treg cell	Antitumor	Glucose metabolism	CD28	Promoting Treg cell function	[380–382]
			GLUT-1	Upregulating the proliferative capacity	[378, 379]
			Foxp3	Inhibiting inhibitory amino acid phosphorylation and reduces mTOR activity	[239, 374]
			MCT1	Promoting cellular function	[389]
			MOESIN	Promoting Treg cell function by modulating TGF-β signaling	[77]
		Lipid metabolism	IL-10	Inducing IL-10 expression and promoting proliferation and differentiation of Tregs	[282, 397]
			SCFA	Inhibiting the efficacy of anti-CTLA-4 therapy and promoting cell proliferation	[395, 396]
			FFAR4	Inhibiting Treg cell function	[398]
			PPARγ	Promoting lipid transport, upregulating FAO, and promoting treg cell function	[400]
			PUFA	Synthesizing membrane phospholipids and producing more ROS, resulting in immunosuppression	[402]
		Amino acid metabolism	SLC7A11	Leading to increased glutamate levels,promoting Treg cell function	[408]
			IDO1	Leading to enhanced differentiation of Tregs	[410]
MDSCs		Glucose metabolism	GLUT	Providing energy for glycolysis in MDSCs and promotes their immunosuppressive effects	[417, 418]
			PI3K-AKT-mTOR	promoting glycolysis and supporting cell proliferation	[414, 419]
			Nrf2	Inhibiting ROS production	[413, 415, 420]
			Lactate	Promoting immunosuppression	[421, 422]
			Notch/RBP-J	Regulating the differentiation and progression of MDSCs by downregulating MCT2 through its downstream effector Hes1	[421, 422]
		Amino acid metabolism	ARG1	Catalyzing the metabolism of Arg to generate energy	[425, 426]
			CAT2	Rendering the environment Arg-deficient	[425, 426]
			IDO1	Suppressing immune effects through competitive consumption of Trp	[428, 429]
			Cys	Suppressing T cells through nutrient competition	[414, 416, 433]
		Lipid metabolism	G-CSF	Higher expression of lipid transporter proteins through STAT3 and STAT5 signaling	[146, 434]
			IL-6	Increasing PGE2 synthesis and inducing cell differentiation	[146, 435]
			Natural plant extracts	Inhibiting FAS and lipid droplet accumulation, reduces cellular energy supply, and induces apoptosis	[436]

## The role of immunometabolism in today's tumor therapy

**Fig. 7:**
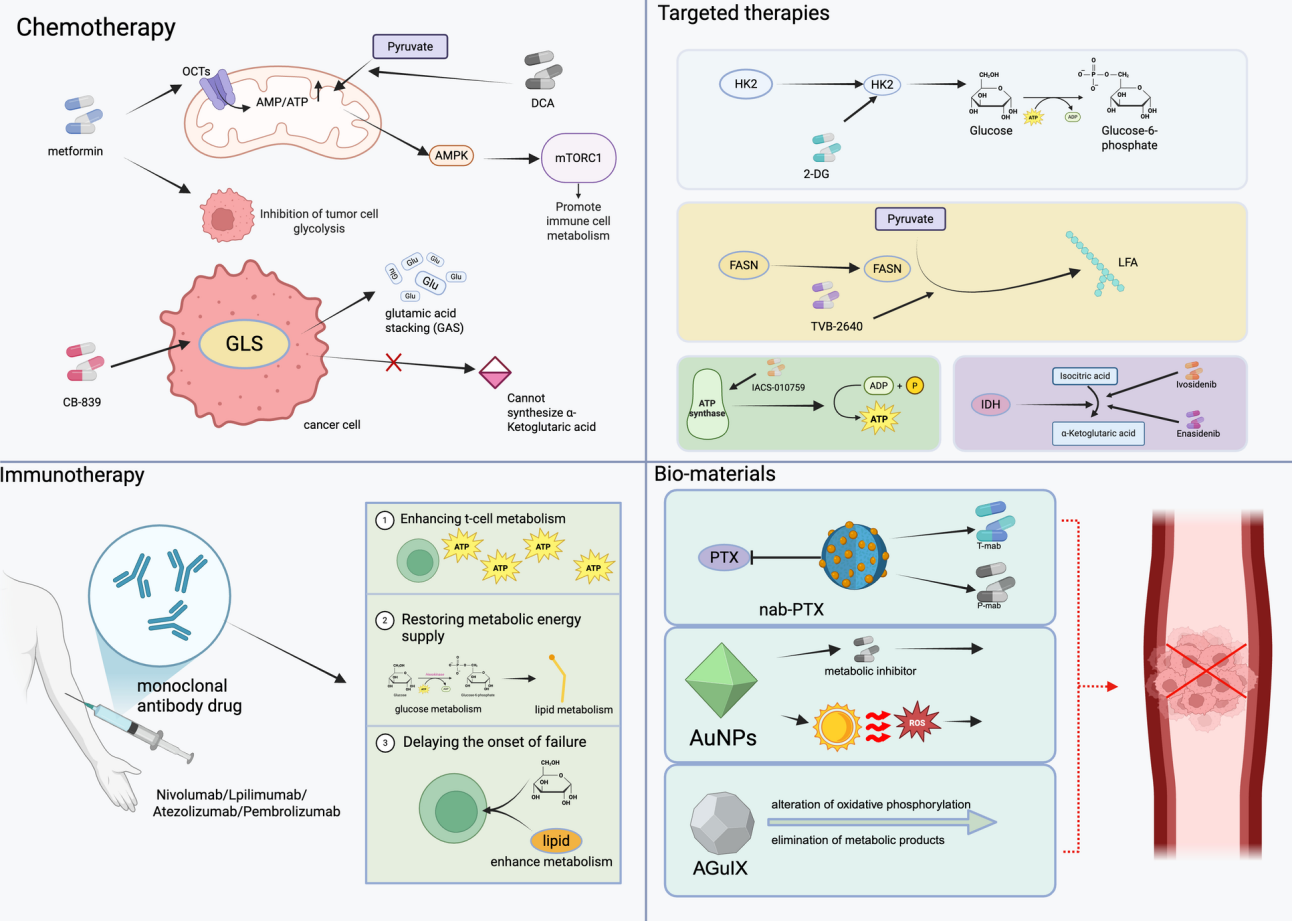
Mechanisms of Immunometabolic Therapy. Metabolism-focused cancer therapies aim to inhibit tumor growth by targeting specific metabolic pathways. Key metabolic agents in chemotherapy include metformin, DCA, and CB-839. Metformin activates the AMPK pathway, while DCA enhances the activity of the pyruvate dehydrogenase complex, thereby suppressing tumor glycolysis. CB-839 inhibits glutaminase (GLS), leading to an accumulation of glutamate and metabolic disruption within tumor cells. These metabolism-targeted therapies act on crucial metabolic enzymes such as hexokinase 2 (HK2), fatty acid synthase (FASN), ATP synthase, and isocitrate dehydrogenase (IDH), thus impeding tumor progression. Furthermore, metabolism-based immunotherapy enhances T cell function by improving T cell metabolism, remodeling energy supply, and delaying T cell exhaustion. Additionally, metabolism-related biomaterials—such as nab-PTX, AGuIX, and photothermal therapy (PTT)—deliver anti-tumor effects through mechanisms such as drug delivery, photothermal therapy, and the generation of reactive oxygen species (ROS). Created with BioRender.com. *OCTs* Organic Cation Transporters, *DCA* Dichloroacetate; *AMPK* AMP—Activated Protein Kinase, *mTORC1* Mammalian Target of Rapamycin Complex 1, *GAS* Glutamic Acid Stacking, *HK2* Hexokinase 2, *2—DG* 2—Deoxyglucose, *LFA* Long—Chain Fatty Acid, *IDH* Isocitrate Dehydrogenase, *PTX* Paclitaxel, *nab—PTX* Nanoparticle—Albumin—Bound Paclitaxel, *AuNPs* Gold Nanoparticles

In recent years, the discovery of the TME metabolism has promoted the progress of tumor treatment. New methods and theories have emerged in the direction of chemotherapy, targeted therapy, and immunotherapy, which provide new ideas for the future cancer attack [437]. The metabolism within the TME is highly intricate, with significant interplay between different metabolic pathways. These interactions may influence the efficacy of diverse therapeutic approaches. However, our understanding remains limited due to insufficient research and experimental evidence. Therefore, we provide a summary of existing metabolic therapies targeting tumors. (Fig. 7).

### Chemotherapy

Chemotherapy is one of the most common treatments for tumors, and the use of metabolic therapy in combination with chemotherapy has given rise to many novel therapeutic approaches:

#### Metformin

In addition to inhibiting glucose metabolism, modulating the competition for glucose with tumor cells represents a reasonable therapeutic strategy. Metformin, a classic drug for the treatment of diabetes, has also demonstrated antitumor effects, primarily through two key mechanisms: the first mechanism is the activation of the AMPK pathway, where metformin enters the mitochondria through the organic cation transporter proteins (OCTs), stimulates mitochondrial complex 1 coupling and leads to redox changes and an increase in the AMP/ATP ratio, ATP further stimulates LKB1, the kinase of AMPK, which in turn continues to stimulate downstream pathways. mTOR, as mentioned earlier, promotes the glucose metabolism activity of various immune cells, and the activation of AMPK inhibits mTOR1 and activates mTOR2 to increase glucose entry into the muscle for metabolism. In addition to mTOR, AMPK inhibits ACC activity, thereby suppressing FAS by reducing the conversion of acetyl coenzyme A, which leads to decreased lipid accumulation and ultimately inhibits tumor growth [438]. The second mechanism is to improve the metabolic environment by altering the TME to make it less favorable for tumor cell growth. Metformin achieves this by inhibiting glycolysis–-particularly aerobic glycolysis, which tumor cells rely on for rapid proliferation, thereby disrupting their energy supply [439].

Metformin has been shown in animal studies to re-sensitize drug-resistant tumors to chemotherapy [440] and works by altering the metabolism of T-cells to use other energy sources, thus avoiding competition for glucose.

Now we look up recent clinical trials using metformin to treat tumors: In a recent trial (NCT04374192) designed by an experimental team to observe the use of metformin in Li-Fraumeni syndrome (LFS), the safety, toxicity, and acceptability of metformin were investigated by randomizing in a 1:1 ratio a sample of mid-224 adults with LFS aged ≥ 16 years to receive either oral metformin (up to 2 mg per day) plus annual MRI monitoring or annual MRI alone for 5 years. The effect of metformin on quality of life and the impact of lifestyle risk factors on cancer incidence will be assessed [441].

In recent years there has been a phase I trial of metformin (NCT01529593) in combination with vincristine, irinotecan, and temozolomide for the treatment of relapsed or refractory solid tumors and CNS tumors in children to study the maximum tolerated dose (MTD) and recommended phase II dose (RP2D), which tested metformin at five doses, 666, 999, 1333, 1666, and 2000 mg/m2/day, with the final trial identified 1666 mg/m2/day as the optimal dose and observed radiological responses in multiple tumor types, which will also be evaluated in further phase II trials [442].

Meanwhile, it has recently been reported that tesirolimus in combination with metformin for the treatment of patients with advanced cancer will be tested at up to 6 dose levels of tesirolimus and metformin, up to 6 participants will be recruited at each dose level, the first group of participants will receive the lowest dose level to find the most appropriate dose, the trial has not yet been completed and we are looking forward to the results of the trial (NCT01529593). Metformin has been used clinically among pancreatic cancer patients with good results [443].

#### CB-839

In addition to glucose metabolism, glutamine is an important energy source for tumor cells. Inhibition of glutamine metabolism can enhance the effect of chemotherapeutic agents and has been applied to chemotherapy regimens [444]. For example, CB-839 has been shown to improve the apoptotic effect of cisplatin in a model of triple-negative breast cancer [445], and also achieved good results in clinical trials [446, 447].

CB-839 is an experimental small-molecule drug primarily acting as a selective GLS inhibitor. It is designed to intervene in the energy metabolism of tumor cells, particularly those cancers dependent on glutamate metabolism, by inhibiting GLS. The structure and function of CB-839 are primarily related to its effects on cellular metabolic pathways and its potential in oncology therapy. CB-839 contains aromatic thiobenzenes structures along with amino and sulfonyl functional groups in its molecular framework, which can selectively inhibit GLS. This inhibition reduces the production of α-ketoglutarate in tumor cells, disrupting cellular energy metabolism. Specifically, the anti-tumor effect of CB-839 is mainly through the following two mechanisms: firstly, GLS inhibition disrupts glutamate metabolism, leading to the accumulation of glutamate in tumor cells; secondly, it causes metabolic disruption, causing tumor cells to lose their ability to synthesize α-ketoglutarate, which is essential for their rapid proliferation and survival. Thus, inhibition of GLS may lead to the growth restriction of cancer cells [447].

In recent preclinical studies on chronic lymphocytic leukemia, researchers studied various cell lines and tested the effects of CB-839 alone and combined with ibrutinib and venetoclax. The results showed that ROS metabolism was increased, and other energy metabolism was impaired in CB-839-treated cells, and cell proliferation was significantly inhibited, but the effects of the combination therapy were not very promising. Therefore, better results may be achieved with CB-839 alone [448].

In a phase I clinical trial (NCT01528046) investigating the synergistic effect of CB-839 and azacytidine (AZA) in advanced myelodysplastic syndromes (MDS), treatment was well tolerated in 24 participants, and complete remission was achieved in 53% of participants (bone marrow). These findings demonstrated the effectiveness of CB-839 and AZA as a combination metabolic therapy [449].

To determine the maximum tolerated doses of CB-839 and capecitabine that can be safely given to patients without too many side effects, the research team tested how many patients had their tumors shrink with this drug combination, as well as the changes that occurred within the cancer cells and blood cells after treatment, culminating in a two-phase study with single incremental injections of the drug in 50 patients (NCT02861300).

A team of researchers has also studied the therapeutic effects of CB-839 alone in solid tumors (NCT02071862), which is divided into two parts. Part 1 is a dose-escalation study enrolling patients with locally advanced, metastatic, and refractory solid tumors to take CB-839 capsules orally two or three times per day. In Part 2, patients with solid tumors will be recruited, and all patients will ultimately be evaluated for safety, pharmacokinetics (plasma concentrations of the drug), pharmacodynamics (GLS inhibition), biomarkers (biochemical markers that may be predictive of responsiveness in later studies), and tumor response.

#### DCA

DCA is a small-molecule compound widely used in clinical and experimental research, especially in metabolic therapy and cancer treatment [450]. DCA facilitates the entry of pyruvate into the mitochondria and aerobic metabolism by activating the pyruvate dehydrogenase complex (PDC) in the mitochondria. Normally, tumor cells rely on glycolysis (Warburg effect) for energy production rather than mitochondrial OXPHOS. DCA reduces lactate production by promoting pyruvate entry into the mitochondria, reversing the bias of glycolysis, and restoring aerobic metabolism to normal cells [451].

DCA, as a metabolic intervention drug, has been investigated in several fields, especially in cancer therapy, where DCA improves the sensitivity of cancer cells to conventional chemotherapy and radiotherapy by adjusting the metabolic pattern of tumor cells. It has been tested in preclinical and clinical trials in several types of cancer (e.g., breast cancer, lung cancer, etc.).

The most recent clinical trial of DCA (NCT05120284) is a recently published study of glioblastoma multiforme (GBM) in which the team recruited 40 patients with GBM who were scheduled to undergo surgery. Participants received oral DCA treatment for one week before their surgery, and surgical samples were collected for analysis.

### Targeted therapy

Targeted therapy treats specific molecules or gene fragments in the tumor cells or the TME, which is less harmful to the surrounding tissues than chemotherapy and radiotherapy. Therefore, with the help of some research results of metabolic therapy, we can aim at the key enzymes in the process of glucose metabolism, lipid metabolism, and amino acid metabolism, and inhibit or enhance them to achieve good therapeutic effects. The feasibility of this idea was recently demonstrated by restoring antigen presentation in a mouse model given the fatty acid metabolism inhibitor etomoxir [452], and not only the anti-tumor presentation of immune cells but also in another study, the research team also used the targeting of FATP2 to inhibit fatty acid accumulation in a mouse model to enhance the function of PD-L1, which achieved the therapeutic effect of tumor treatment [453]. Although these experiments confirm the feasibility of targeted metabolic therapies in tumor treatment, we will describe the targeted therapies represented by the metabolic panel below.

#### Representative targets in glucose metabolism: HK2

HK2 is an enzyme responsible for catalyzing the conversion of glucose to glucose-6-phosphate (G-6-P) in cells. It is a type of hexokinase that is usually localized near the outer membrane of mitochondria, where it helps to interact with other proteins in the mitochondrion. HK2 is important in many tissues, especially in highly metabolically active cells such as muscle, liver, and brain. It is a key enzyme in glucose metabolism, converting glucose or other similar six-carbon sugars into the corresponding phosphorylated forms, which is the first step in the cell's utilization of these sugars [454]. In some tumor cells, HK2 expression activity is usually elevated, which is associated with rapid tumor growth with high metabolic demands [455], so some recent studies have focused on targeting HK2 to inhibit tumor growth by disrupting glucose metabolism and reducing the energy supply to the tumor.

2-Deoxyglucose (2-DG) is a glycolysis-inhibiting drug that targets HK2, a synthetic glucose analog that contains a 2-deoxy, β-d-pyran structure. In terms of action, glucose transporter proteins and glucose transport it to the effector region. After reaching the effector region, 2-DG is phosphorylated by HK2 to 2-deoxy-d-glucose-6-phosphate (2-DG-6-P), which lacks the 2-OH group and cannot be isomerized to fructose-6-P [456], so that 2-DG-6-P is capable of accumulating intracellularly and inhibiting glucose metabolism in tumor cells [457]. Also, 2-DG is important in treating brain tumors because it can cross the blood–brain barrier via glucose transporter proteins [458]. Besides inhibiting glycolysis, 2-DG also exerts anti-tumor effects by down-regulating anti-apoptotic related proteins and receptors, and by generating ROS to promote apoptosis in tumor cells [459].

Existing preclinical studies plan to use 2-DG as an adjuvant to chemotherapy for prostate, liver, and pancreatic cancer to promote the therapeutic effects of related chemotherapeutic agents [460–462]. Good results have been achieved in the past and concomitant combination with cisplatin [463], doxorubicin [464], and gefitinib [465].

During a phase I clinical trial investigating 2-DG in combination with doxorubicin for the treatment of patients (NCT00096707) with advanced solid tumors, researchers administered dose-escalating doses to 34 patients, and at a combined weekly gradient of doxorubicin 63 mg/kg/day. The majority of patients experienced significant remission of their disease. Although side effects such as fatigue, sweating, dizziness, and nausea were reported, the adverse effects of co-administration with doxorubicin (63 mg/kg/day) were considered tolerable in this trial [466].

In a separate phase I/II clinical trial (NCT00633087), the team is investigating the optimal dose of 2-DG in prostate cancer, with patients being given a daily oral solution of 2-deoxyglucose. Before starting the study, they will be asked to have a CT scan, a bone scan, and an optional PET (positron emission tomography) scan. The PET scan will also be performed shortly after the start of the study and after two cycles (6 weeks) of treatment. Subjects will be asked to undergo a complete physical examination and blood tests before the start of the study. During the first two cycles of the study, subjects will be asked to have blood drawn at different intervals on the first 2 days of each cycle. They will be asked to return to the clinic for a physical examination and blood tests every 1 week for the study duration. Subjects will also be asked to have CT scans and bone scans every 9 weeks during the study. However, this clinical trial was ultimately unsuccessful.

#### Representative targets in lipid metabolism: FASN

Fatty acid synthase (FASN) is responsible for the intracellular synthesis of long-chain saturated fatty acids. It is one of the key enzymes in lipid metabolism, converting pyruvate and elongation units to LCFAs through a series of reactions, which is accomplished through a multifunctional enzyme complex in which multiple enzyme active domains work synergistically. The expression of FASN is significantly elevated in a wide range of tumor cells, supporting tumor cells'lipid requirements and promoting lipid accumulation, which results in immune escape. FASN contributes to constructing new cell membranes and providing energy to tumor cells by promoting FAS, making it a potential target in cancer therapy [467].

In a phase II trial(NCT02595372) to investigate the association between FASN inhibition and triple-negative breast cancer (TNBC), the research team treated 42 patients with oral omeprazole and observed the complete remission rate (pCR) of the surgical patients, the changes of FASN expression, enzyme activity, and downstream protein expression after omeprazole monotherapy, the safety and pharmacokinetics of omeprazole, etc., of which the pCR rate of all surgical patients was 74.4% (95% CI 57.9–87.0). We can conclude from this experiment that FASN expression in early TNBC is an essential element of its progression, and at the same time, omeprazole can be used to treat tumors by inhibiting FASN as a target, and further validation will be carried out in the follow-up of this experiment [468].

In the phase II clinical trial (NCT03032484), the research team investigated TVB-2640 combined with bevacizumab for treating glioblastoma.TV-2640 is a FASN inhibitor, and the team randomly assigned 25 patients to be treated with either TVB-2640 (100 mg/m2 orally daily) plus bevacizumab (10 mg/kg i.v., D1 and D15) or bevacizumab monotherapy for cycle 1 (28 days) only. Bevacizumab monotherapy for Cycle 1 (28 days) was used for treatment, and it was ultimately found that the overall effectiveness rate (ORR) of TVB-2640 plus bevacizumab was 56%. Some patients developed abnormalities such as palmar-plantar erythema, thus demonstrating that the combination of TV-2640 and bevacizumab is a safe and reliable targeted therapy [469].

In a separate trial (NCT03179904), the team evaluated the effectiveness of TVB-2640, paclitaxel, and trastuzumab in treating patients with metastatic HER2-positive breast cancer. The study enrolled 19 patients and was planned to evaluate the ORR of TVB-2640 in combination with paclitaxel and trastuzumab in patients with paclitaxel- and trastuzumab-resistant advanced HER2-positive breast cancer, and changes in FASN, phosphorylated (p)AKT, and pS6 expression in the tumor tissues after the first cycle of the treatment. The study is currently ongoing.

Another drug that works by targeting FASN is TAS-118. In a recent clinical trial (NCT02322593), the team compared the overall survival of TAS-118 plus oxaliplatin versus S-1 plus cisplatin in advanced gastric cancer, which enrolled 711 patients. During the trial, the 711 patients were randomized to TAS-118 plus oxaliplatin (n = 356) or S-1 plus cisplatin (n = 355) and followed up at 12-week intervals, with the longest follow-up lasting 38 months. The final results showed that TAS-118, in combination with oxaliplatin, demonstrated a clinically meaningful improvement in efficacy compared to S-1 in combination with cisplatin [470].

#### Representative targets in OXPHOS metabolism: ATP synthase

ATP synthase is responsible for the intracellular synthesis of ATP, the main energy currency in the cell, which is required for almost all cellular activities. ATP synthase plays a crucial role in cellular energy metabolism, especially in mitochondria, chloroplasts, and bacteria [471]. The most crucial function of ATP synthase is to use the proton pump to drive ATP synthesis. The proton pump consists of two parts: the F₀ part and the F₁ part. The F₀ part is mainly responsible for the flow of protons. Protons enter the ATP synthase complex through the proton channel in the F₀ part. The F₁ part is responsible for ATP synthesis. While protons flow through the F₀ part, rotation of the F₁ part drives ATP synthesis. As protons flow through the F₀ channel, their flow drives the rotational motion of the F₁ portion, which allows the combination of ADP and inorganic phosphate to synthesize ATP [472]. Although tumor cells often provide energy through glycolysis, some tumors still rely on mitochondrial OXPHOS to produce ATP. Thus, OXPHOS has also emerged as a potential target for tumor therapy.

In a recent phase I clinical trial (NCT02882321), researchers treated 17 patients with relapsed/refractory acute myeloid leukemia and 23 patients with advanced solid tumors using IACS-010759. Still, most of the subjects experienced side effects, including elevated blood lactate and neurotoxicity, which hampered the trial. These findings suggests that IACS-010759 may have safety concerns, particularly regarding neurotoxicity, and further studies are needed to clarify the underlying relationship [473]. In a phase I clinical trial (NCT03291938), researchers tested the side effects and optimal dose of IACS-010759 in 29 patients with advanced, metastatic, recurrent, or refractory solid tumors that were unresponsive to treatment or unresectable. Patients received dose-escalating treatment during the cycle and were followed up after treatment, and the trial was completed.

#### Representative targets of amino acid metabolism: IDH

IDH is an important class of enzymes that plays a key role in cellular metabolism. It is found mainly in the mitochondria and cytoplasm of cells, and its function is to convert isocitric acid to α-KG, with the concomitant production of NADH or NADPH. This process is of great importance in energy metabolism and cellular biology. The IDH-catalyzed conversion of isocitrate to α-ketoglutarate(α-KG), and α-KG plays several vital roles in cellular metabolism, including serving as an intermediate in the TCA, helping to generate ATP and NADH. α-KG serves as a precursor for several metabolic pathways, including the synthesis of amino acids and certain signaling molecules, and also participates in regulating intracellular redox homeostasis [474].

Mutations in IDH1/2 are strongly associated with certain types of cancer (e.g., glioma, leukemia, etc.). In particular, its mutation leads to an altered catalyzed reaction that produces an abnormal metabolite, 2-HG, instead of the normal alpha-ketoglutarate. 2- HG is carcinogenic because it affects a variety of metabolic pathways, in particular through inhibition of certain alpha-ketoglutarate-dependent enzymes (e.g., demethylases and deacetylases) to alter the gene expression and epigenetic status of cells. Therefore, treating tumors by targeting IDH is currently a feasible direction [475].

Ivosidenib is an inhibitor that targets IDH1 and is generally used to treat acute myeloid leukemia (AML) and other types of cancer that carry IDH1 mutations. In a recent clinical trial (NCT03564821), a team of researchers conducted a phase I clinical trial of the potential of Ivosidenib as a treatment for IDH1-mutated myeloid tumors after allogeneic stem cell transplantation. The trial included 18 patients, who are divided into two groups receiving 250 mg and 500 mg of the drug per day. This trial aimed to determine the maximum tolerated dose of the drug and to record the occurrence of adverse events [476]. In a separate clinical trial (NCT06291987), researchers investigated safety and efficacy information for ruxolitinib in combination with ivosidenib for patients with advanced Ph-negative MPN with IDH1 mutations, while evaluating the efficacy of ruxolitinib in combination with ivosidenib.

Enasidenib is an inhibitor that targets IDH2, distinct from ivosidenib above, and is intended primarily as a therapeutic agent for acute myeloid leukemia with IDH2 mutations. In a recently completed clinical trial (NCT01915498), the research team conducted a phase I/II clinical trial of enasidenib in advanced hematologic malignancies with IDH2 mutations. 345 trial patients were tested for safety and tolerability with continuous oral administration of enasidenib as a single agent on days 1 through 28 of a 28-day cycle. The efficacy of enasidenib in the treatment of relapsed or refractory acute myeloid leukemia AML with IDH2 mutations was assessed by the ORR, and it was found that enasidenib significantly prolonged patient survival.

A recent clinical trial (NCT06176989) used enasidenib for malignant tumors of the sinuses as well as the base of the skull. It was based on the overall progression-free survival (PFS) of all study participants with IDH2m malignant sinus and base of the skull tumors treated with enasidenib. The trial is currently undergoing patient enrollment.

### Immunotherapy

Immunotherapy for tumors is the process by which we artificially activate the patient's immune system so that it can rely on its immune function to kill tumor cells and tissues. Unlike chemotherapy and targeted therapy, immunotherapy targets the body's immune system [477]. The metabolic state of tumor cells and immune cells has a profound impact on tumor immune escape, immunosuppression, and the effectiveness of immunotherapy. Many studies have shown that alterations in metabolic pathways in the TME [478], including changes in glucose metabolism, fatty acid metabolism, and amino acid metabolism, significantly influence the function of immune cells. These metabolic changes can lead to extracellular acidification, facilitating tumor cells'immune evasion. Enhancing immune cell metabolism and restoring functional capacity may help reduce tumor immune escape. This represents a promising emerging strategy for improving the efficacy of immunotherapy [479]. In the following, we will describe several existing mechanisms of metabolic immunotherapy and classical drugs.

#### Mechanisms of immunometabolic reprogramming for tumor treatment

Tumor cells and immune cells are often in a state of metabolic reprogramming. By adjusting the metabolic pathways of these cells, immune cell function can be enhanced and tumor growth inhibited [480].

By gifting enhanced T cell metabolic function, such as immune checkpoint inhibitors, it is possible to deregulate immune cell suppression and enhance the immune response of T cells to tumors [481]. However, these immune checkpoint inhibitors are not always effective in many patients, partly because T cell function may be limited due to metabolic disorders. In such cases, metabolic reprogramming—particularly targeting glucose and fatty acid metabolism—may help restore T cell activation and function. In the TME, tumor cells inhibit T cell metabolism and function by competing for glucose and other nutrients. By restoring glucose uptake in T cells through metabolic reprogramming or targeting fatty acid metabolism, we can improve the effector functions of T cells and thereby enhance immune responses [482]. In addition to the above immune checkpoint inhibitors and metabolic reprogramming, in the TME, T cells often experience “immune exhaustion”, i.e., they lose their ability to attack tumors. By targeting metabolic pathways, such as increasing the uptake of fatty acids or glucose by T-cells, T-cell exhaustion can be slowed down, thus improving the efficacy of immunotherapy [483].

#### Preclinical and clinical studies of immunometabolic reprogramming therapeutics

Navulizumab is an immune checkpoint inhibitor that belongs to the monoclonal antibody class of drugs. It enhances the ability of the patient's immune system, especially T-cells, to recognize and clear tumors by inhibiting the immune escape mechanism of tumor cells. Navulizumab mainly acts on PD-1, an immune checkpoint receptor expressed on the surface of immune cells such as T cells, B cells, and DCs. Tumor cells bind to PD-1 by expressing PD-L1, thereby inhibiting the immunometabolic activity of T cells and avoiding recognition and attack by the immune system. Navulizumab, an anti-PD-1 antibody, can bind to PD-1, block the interaction between PD-1 and PD-L1, thereby relieving the metabolic inhibition of T cells, restoring the function of T cells, and enhancing the immune system’s ability to recognize and eliminate tumor cells [484].

Recently, a team of researchers investigated the safety of natalizumab alone or in combination after completion of radiotherapy in gliomas (NCT02311920), and showed favorable results in 32 patients, with a grade 4 event rate of 16%, and no side effects with the combination, providing a favorable basis for subsequent combination trials in a phase I clinical trial [485].

In another recent phase I trial (NCT02614456) investigating the safety of combining IFN-γ and natalizumab in the treatment of metastatic solid tumors, 26 patients in four cohorts were treated with natalizumab in combination with increasing doses of IFN-γ. A safe dose of IFN-γ was obtained at a dose of 75 mcg/m2, and it was also observed that IFN-γ was shown to induce circulating chemokine production, a phenomenon that may await further study by the research team [486].

In a preclinical trial utilizing nivolumab (NCT05476796), researchers plan to combine it with fluorouracil/tipiracil (FTD/TPI) and oxaliplatin for the treatment of metastatic gastric and gastroesophageal junction adenocarcinoma, which includes 118 patients. The main objective is to evaluate the effectiveness of FTD/TPI and oxaliplatin in combination with nabulizumab for treating gastroesophageal cancer, and we are also closely following the progress of this trial [487].

Ipilimumab is an immune checkpoint inhibitor mainly used in the treatment of cancer, especially advanced melanoma [488]. Its mechanism of action is to enhance the metabolic response of T cells by inhibiting CTLA-4, thus helping the immune system to recognize and attack tumor cells [489]. CTLA-4 is an immune checkpoint molecule expressed on the surface of T cells, which normally inhibits the activation and proliferation of T cells by binding to ligands of the B7 family, thus suppressing the immune system's response to tumors [490]. 

Another research team has conducted a study (NCT05753839) to investigate the role of cytoreductive nephrectomy (CN) in combination with ibritumomab in the treatment of metastatic renal cell carcinoma (mRCC). The trial began in 2022 andplans to enroll 55 patients. The primary endpoint is overall survival, while secondary outcomes include objective response rate, incidence of treatment-related adverse events, and complications. Additionally, the study will analyze the gene profiles of tumor tissue, circulating tumor DNA, urinary tumor DNA, and tumor-infiltrating lymphocytes. We will continue to monitor the results of this trial [491].

Atezolizumab, as an immune checkpoint inhibitor, enhances the immune system's attack on tumors mainly by targeting PD-L1, blocking the binding of PD-L1 to PD-1, disarming the immune escape mechanism of tumor cells, as well as enhancing the activity of T cells and promoting the clearance of metabolites (e.g., lactic acid), thus improving the acidic conditions of the TME and indirectly affecting metabolic pathways [492].

In a recent clinical trial (NCT02323191), researchers studied the combination of emactuzumab and atezolizumab in participants with advanced solid tumors and evaluated its safety, pharmacokinetics, and activity. The 221 patients enrolled in the study were treated, and results showed that the combination demonstrated an easily controlled safety profile compared to usual atezolizumab treatment, but was associated with more fatigue and rash.

A separate investigator evaluated the safety, tolerability, pharmacokinetics, immunogenicity, and preliminary efficacy of atezolizumab in pediatric and young adult participants with solid tumors (NCT02541604). The trial recruited 87 patients and ultimately concluded that while the response to atezolizumab was limited, it was well tolerated, with exposure levels generally comparable across populations.

Pembrolizumab is an anti-PD-1 monoclonal antibody used in tumor immunotherapy, especially for treating many cancers such as non-small cell lung cancer, melanoma, head and neck cancer, bladder cancer, etc [493–495]. It enhances the immune system's attack on tumors by targeting PD-1 and lifting the immune system's immunosuppression. In this progression, Pembrolizumab enhances the function of T-cells, enabling them to regain activity and effectively attack tumor cells. Recovered T cells may consume more metabolic resources (e.g., glucose, amino acids, etc.) to support their activity, thereby competing with tumor cells for metabolic substrates, which in turn affects the metabolic demands of the tumor. In addition to enhancing T-cell function, it may alter the TME to reduce the accumulation of lactate and other metabolites, which helps restore the effector function of immune cells, enabling them to fight tumors more effectively.

In a phase I trial (NCT02587455) of 24 patients with advanced lung cancer, researchers combined pembrolizumab with palliative radiotherapy, dividing patients into two groups to give high and low doses of radiation and assessing adverse effects, as well as evaluating survival. Another recently completed clinical trial investigated whether pembrolizumab has antitumor activity in microsatellite unstable tumors (NCT04098068). The trial treated 12 patients with the drug every 21 days. It looked at the objective remission rate (ORR) of patients with malignant tumors, including solid tumors, and overall survival and disease control.

### Biomaterials-related applications

Applying metabolism-related biomaterials in tumor therapy is an emerging and promising research area. Metabolism plays a key role in tumor cell growth, migration, and drug resistance, so targeting metabolic pathways can provide new strategies for tumor therapy. Regulating the metabolism of immune cells in the TME by combining nanomaterials and biomaterials is also known as a hot topic in recent years [496]. The following are several metabolism-related biomaterials and their mechanisms for tumor treatment.

#### Delivery systems for metabolism-modulated drugs

Tumor cells have metabolic features that differ from normal cells, such as increased glycolytic activity and abnormal fatty acid metabolism. Metabolism-modulating drugs can inhibit tumor growth by targeting these metabolic pathways. The targeting and bioavailability of drugs can be improved by utilizing biomaterials as drug delivery systems [497]. Drug delivery systems deliver metabolism-regulating drugs (e.g., glycolysis inhibitors, FAS inhibitors, etc.) to tumor cells through biomaterial carriers such as nanoparticles, liposomes, polymers, etc. to inhibit their metabolic aberrations, reducing the growth and spread of the tumor cells, which is the most dominant approach at present [498]. In the past, using nanomaterials to deliver nucleic acid drugs in combination with chemotherapy to treat tumors has achieved good results in mouse models [499]. There is also a research team that has developed a nanocarrier that can carry a variety of drugs, improving the cost-effectiveness of drug therapy [500]. However, the use of these biomaterials may show some human damage in clinical trials, for example, the use of 2-deoxy-glucose as a carrier to transport drugs has cardiotoxicity [501], so how to use biomaterials for treatment more safely is an essential direction of the current research.

In a recent clinical trial (NCT01730833), the research team conducted a phase I trial of nanoparticle albumin-conjugated paclitaxel (nab-PTX) in combination with trastuzumab (T-mab) and pertuzumab (P-mab) as a neoadjuvant chemotherapy for HER2-positive breast cancer. 22 of 43 patients had HER2, and 21 had luminal/HER2 subtypes. With the luminal/HER2 subtype, the overall remission rate was 53.5%, while the level of side effects was within the acceptable range. This clinical study provides a new option for treating HER2-positive breast cancer and shows the emerging role of nanomaterials in tumor therapy [502].

In another recent phase I trial of albumin-bound paclitaxel plus S-1 versus gemcitabine plus cisplatin as first-line chemotherapy in patients with locally advanced and/or metastatic urothelial migratory cell carcinoma (NCT03051373), researchers divided 108 patients into two groups to conduct a comparative clinical trial of albumin-bound paclitaxel plus S-1 versus gemcitabine plus cisplatin and to assess patients'survival as well as overall response and disease control rates. The trial has now been completed and validated.

AGuIX is a new type of gadolinium-based nanoparticle composed of a silver metal core and a metal shell, which has attracted attention in recent years for its application in tumor therapy, especially tumor radiotherapy. It can inhibit tumor growth by altering OXPHOS and scavenging metabolites. In a recent phase II clinical trial of AGuIX (NCT04899908), researchers analyzed the uptake of AGuIX in 23 brain tumor patients undergoing radiotherapy, with mean tumor uptake ranging between 0.012 and 0.17 mg/ml, while no uptake was observed in patients receiving placebo [503].

In another clinical trial (NCT03818386) using AGuIX to treat metastatic tumors in the brain, researchers plan to enroll 100 patients and compare whole-brain radiation combined with AGuIX to whole-brain radiation alone, and the trial is still enrolling patient.

#### Photothermal therapy (PTT) in combination with metabolic correlation

Photothermal therapy (PTT) uses nanomaterials (e.g., gold nanoparticles) to kill tumor cells and detect tumor cells by generating heat in the presence of light [504]. Metabolism-related biomaterials can enhance the effectiveness of this therapy by using metabolic modulators (e.g., glycolysis inhibitors) in conjunction with photothermal therapy, which is delivered via biomaterials, to alter the metabolic state of the tumor cells, making them more sensitive to thermal stimuli and improving the effectiveness of the therapy. Currently, there are relatively few clinical studies on the combined application of photothermal therapy and metabolic therapy. Still, there have been several relevant preclinical experiments and preliminary clinical trials that provide support for the feasibility of this combined therapy [505].

Several preclinical studies are evaluating the effects of glycolysis inhibitors, such as 2-DG, in combination with photothermal therapy. For example, 2-DG as a glycolysis inhibitor can attenuate the resistance of tumor cells to radiotherapy and photothermal therapy, thereby enhancing the therapeutic effect. Preclinical studies using gold nanoparticles (AuNPs) or other photothermal-sensitive nanomaterials in combination with glycolysis inhibitors have shown significant anti-tumor effects. These promising findings are now gradually being translated into clinical applications [506]. Meanwhile, in another observational trial (NCT04907422), gold nanoparticles were synthesized and conjugated to CD24 primers to form CD24-Gold nanocomplexes. The CD24-Gold nanocomplex was validated as a promising and highly sensitive biomarker for salivary gland tumor diagnosis and prognosis prediction in sixty patients with solid tumors [507].

In addition to glycolysis, ROS has also been linked to photothermal materials. A research team has found that when nanomaterials are exposed to near-infrared (NIR) light, they absorb light energy and convert it into heat, leading to an increase in local temperature and the generation of ROS. ROS induces apoptosis or necrosis by damaging cellular components such as cell membranes, DNA, and proteins. Researchers are exploring how to optimize the generation of ROS by modulating the properties of the nanomaterials, light intensity, and time to achieve efficient and selective killing of tumor cells, and we look forward to subsequent clinical trials of this approach [508].

In a NIR/US Clinical Trial to Assess the Accuracy of Tumor Vasculature and Oxygen Changes in Predicting and Monitoring Early Neoadjuvant Response (NCT02891681), postgraduate researchers enrolled 41 patients, divided into two groups, one with neoadjuvant chemotherapy and the other with neoadjuvant endocrine therapy, to be evaluated after the first cycle of treatment based on the Miller- Payne classification of pathologic response. It was ultimately concluded that the combination provided an accurate prediction of NAT response after the first treatment cycle [509]. The means of photothermal therapy combined with metabolic therapy interact with each other through multiple mechanisms to enhance tumor cells'sensitivity to photothermal therapy and reduce their adaptability through metabolic intervention. The combined effect not only improves the effect of tumor treatment but also helps to improve the TME and reduce the side effects during treatment (Table 8).

**Table 8:** Clinical trials on tumor immunometabolic therapy

Treatment methods	Target metabolism	Drug	Mechanism	Combination agent	Phase	Clinical trial
Chemotherapy	Glucose metabolism	Metformin	Activate AMPK pathway, improve metabolic environment	Vincristine/Irinotecan/Temozolomide	Phase I Phase II	NCT04374192
				Temsirolimus	Phase I	NCT01529593
	Amino acid metabolism	CB-839	Leads to glutamate accumulation in tumor cells as well as metabolic disorders	AZA	Phase I	NCT01528046
				Capecitabine	Phase I Phase II	NCT02861300
				–	Phase I	NCT02071862
	OXPHOS	DCA	Restore normal aerobic metabolism in cells, thereby reducing lactate production	–	Phase I	NCT05120284
Targeted therapies	Glucose metabolism (HK2)	2-DG	Targeting HK2, inhibiting glycolysis and glucose metabolism in tumor cells	Doxorubicin	Phase I Phase II	NCT00096707
				–	Phase I Phase II	NCT00633087
	Lipid metabolism (FASN)	TVB-2640	Tissue tumor cells construct new cell membranes and energy acquisition	Bevacizumab	Phase I	NCT03032484
				Omeprazole	Phase I	NCT02595372
				Paclitaxel	Phase I	NCT03179904
		TAS-118	Inhibiting FASN	Leucovorin/Oxaliplatin/Cisplatin	Phase III	NCT02322593
		Omeprazole	Inhibiting fatty acid synthase	–	Phase I	NCT02595372
	OXPHOS	IACS-010759	Blocking ATP synthesis in tumor cells	–	Phase I	NCT02882321
				–	Phase I	NCT03291938
	Amino acid metabolism (IDH)	Lvosidenib	Inhibiting IDH1	–	Phase I	NCT03564821
				Ruxolitinib	Phase I	NCT06291987
		Enasidenib	Inhibiting IDH2	–	Phase I Phase II	NCT01915498
				–	Phase II	NCT06176989
Immunotherapy	–	Nivolumab		Ipilimumab	Phase I	NCT02311920
				IFN-γ	Phase I	NCT02614456
			Inhibition of CTLA-4 enhances the metabolic response of T cells	Trifluridine/Tipiracil/Oxaliplatin	Phase I	NCT05476796
	-	Ipilimumab	Blocking the binding of PD-L1 to PD-1 and promoting metabolite clearance	SEVURO-CN	Phase I	NCT05753839
				Emactuzumab	Phase I	NCT02323191
		Atezolizumab	Enhancing T cell function and modifying the tumor microenvironment	–	Phase I Phase II	NCT02541604
				–	Phase I	NCT02587455
		Pembrolizumab	Delivering metabolic modulators and inhibiting metabolic abnormalities	–	Phase II	NCT04098068
				Pertuzumab/Trastuzumab/Paclitaxel	Phase II	NCT01730833
Bio-materials	–	Nannoparticle Formulatiion	Modifying OXPHOS and clearing metabolic byproducts	Gemcitabine Plus Cisplatin	Phase II	NCT03051373
				–	Phase II	NCT04899908
		AGuIX	Generating heat under light irradiation to kill tumor cells	–	Phase II	NCT03818386
			Absorb light energy and convert it into heat energy, generating ROS	CD24	Phase I	NCT04907422
		AuNPs		US	Phase I	NCT02891681
		NIR				

## Conclusions and perspectives

TME represents a dynamic ecosystem where immune cell metabolism emerges as a pivotal regulator of tumor progression, therapeutic resistance, and immune evasion. This intricate interplay between metabolic reprogramming and immune function underscores the dual role of immune cells as both guardians and accomplices in cancer biology. Hypoxia, nutrient competition, and acidosis within the TME drive metabolic adaptations that polarize immune cell phenotypes—shaping antitumor responses or fostering immunosuppression. For instance, glycolytic M1 macrophages and OXPHOS-dependent M2 macrophages exemplify how metabolic pathways dictate functional dichotomy, while T-cell exhaustion and Treg expansion highlight the metabolic constraints imposed by the TME.

## Key mechanistic insights and unresolved questions

*Metabolic Crosstalk and Heterogeneity* While glycolysis, FAO, and amino acid metabolism are recognized as central to immune cell function, their spatiotemporal regulation and crosstalk within the TME remain poorly understood. Most existing studies rely on bulk omics or oversimplified in vitro models, which fail to recapitulate the spatial and temporal complexity of metabolic crosstalk in vivo. Consequently, conclusions drawn may not accurately reflect the functional heterogeneity of immune cells within solid tumors. Single-cell and spatial metabolomics could unravel metabolic heterogeneity among immune subsets and tumor niches, addressing how localized nutrient gradients or mitochondrial dynamics influence immune cell fate.

*Immune-Escape Mechanisms* Lactate accumulation, kynurenine production, and cholesterol dysregulation are established drivers of immunosuppression. However, the role of post-translational modifications (e.g., lactylation, acetylation) in metabolic rewiring and epigenetic regulation warrants deeper exploration. Despite emerging interest, most findings on these modifications remain correlative, lacking causal mechanistic validation or functional specificity across immune cell subsets. Without this, therapeutic targeting remains speculative.

*Therapeutic Resistance* Metabolic plasticity enables tumors to evade targeted therapies and immunotherapies. For example, PD-1 blockade failure in “cold” tumors may stem from T-cell metabolic insufficiency due to glucose deprivation or mitochondrial dysfunction. Identifying compensatory pathways (e.g., glutamine dependency) could inform resistance-mitigation strategies.

Nevertheless, metabolic compensation is highly context-dependent, and broad-spectrum inhibitors often fail due to systemic toxicity or metabolic redundancy. This calls into question the clinical feasibility of monotherapies targeting single metabolic nodes.

## Future directions: bridging discovery and translation

*Multi-Omics Integration* Combining single-cell RNA sequencing, spatial metabolomics, and flux analysis will map metabolic networks across immune and tumor cells, revealing context-dependent vulnerabilities. Machine learning algorithms could predict metabolic dependencies based on tumor subtype or TME composition.

### Innovative therapeutic modalities

*Metabolic Checkpoints* Targeting enzymes like IDO1, ACLY, or CPT1A may reprogram immunosuppressive niches. Dual inhibitors (e.g., HK2/PD-L1) or metabolite scavengers (e.g., lactate-neutralizing nanoparticles) could restore antitumor immunity.

*Combination Strategies* Pairing immune checkpoint inhibitors with metabolic modulators (e.g., AMPK activators and GLS inhibitors) may overcome microenvironmental constraints. For example, metformin-enhanced T-cell fitness could synergize with CAR-T therapies.

*Personalized Metabolic Profiling* Clinically, liquid biopsies or imaging-based metabolomics (e.g., hyperpolarized MRI) could stratify patients for tailored therapies. Biomarkers such as circulating succinate or Arg levels might predict immunotherapy responsiveness.

*Clinical Translation Challenges* While preclinical models highlight metabolic targets, their clinical efficacy requires validation in phase III trials. Challenges include optimizing drug delivery to immune-tumor interfaces, minimizing off-target effects, and addressing interpatient metabolic variability. Furthermore, a disconnect between preclinical research priorities and clinical trial design often results in missed translational opportunities. Overemphasis on “target novelty” without addressing pharmacodynamic limitations undermines therapeutic success.

## Toward a new era of immunometabolic oncology

The convergence of immunology and metabolism heralds a paradigm shift in cancer therapy. By decoding the metabolic “language” of the TME, we can design interventions that reprogram immune cells from bystanders to warriors. Emerging tools—CRISPR screens, organoid-TME models, and AI-driven drug discovery—will accelerate this transition. Ultimately, the goal is not merely to inhibit cancer metabolism but to harness it, transforming the TME into a battlefield where immune cells regain dominance.

This vision demands interdisciplinary collaboration, robust biomarker-driven trials, and a commitment to translating mechanistic insights into therapies that extend survival and improve the quality of life for cancer patients. While these tools offer unprecedented resolution and scalability, overreliance on high-throughput platforms risks producing data without actionable insight. Integrating mechanistic depth with clinical pragmatism remains a major unmet need.

## Data Availability

No datasets were generated or analysed during the current study.

## Abbreviations

2-DG: 2-Deoxyglucose
ABCA1/G1: ATP-binding cassette A1/G1
ADCC: Anti-tumor roles by killing tumor cells through cytotoxicity
AMPK: AMP-activated protein kinase
Arg: Arginine
AZA: Azacytidine
CCR7: Chemokine Receptor-7
CD36: Cluster of differentiation 36
COX: Cyclooxygenase
CPT1A: Carnitine palmitoyltransferase-1A
CYP27A1: Cytochrome P450 family 27 subfamily a member 1
DC: Dendritic cells
DDR: DNA damage response
E2F: E2F transcription factors
FAO: Fatty acid oxidation
FASN: Fatty acid synthase
FATP2: Fatty acid transporter protein 2
FBP: Fructose-1,6-bisphosphatase
FoxP3: Forkhead transcription factor 3
G6PD: Glucose 6-phosphate dehydrogenase
GLS: Glutaminase
GLUT1: Glucose transporter protein 1
GOT2: Glutamic-oxaloacetic transaminase 2
GPR81: G protein-coupled receptor
GS: Glutamine synthetase
HDAC: Histone deacetylase
HIF-1α: Hypoxia-inducible factor 1 alpha
HK2: Hexokinase 2
HMGB1: High mobility group protein B1
ICI: Immune checkpoint inhibitors
IDO: Indoleamine 2,3-dioxygenase
IHC: Immunohistochemistry
Kyn: Kynurenine
LAG3: Lymphocyte-activation gene 3
LCAT: Lecithin-cholesterol acyltransferase
LOX: Lipoxygenase
LXR: Liver X Receptor
MDSC: Myeloid-derived suppressor cells
MHC: Major Histocompatibility Complex
METTL5: Methyltransferase-like 5
MGLL: Monoglyceride lipase
mitoDAMP: Mitochondrial damage-associated molecular pattern
NETs: Neutrophil extracellular traps
NK cells: Natural killer cells
NSCLC: Non-small cell lung cancer
NOS: Nitric oxide synthase
OxLDL: Oxidized low-density lipoproteins
OXPHOS: Oxidative phosphorylation
PD-L1: Programmed death-ligand 1
PGE2: Prostaglandin E2
PI3K/AKT/mTOR: Phosphatidylinositol 3-kinase/protein kinase B/mammalian target of rapamycin
PPAR: Peroxisome proliferator-activated receptor
PPP: Pentose phosphate pathway
RIPK3: Receptor-interacting protein kinase 3
RNI: Reactive nitrogen intermediates
ROS: Reactive oxygen species
SCFA: Short-chain fatty acids
SIX1: Sinus homology cassette homolog 1
SREBP: Sterol regulatory element-binding protein
SUCLA2: Succinate-CoA ligase ADP-forming beta subunit
TAMs: Tumor-associated macrophages
TANs: Tumor-associated neutrophils
TCA: Tricarboxylic acid cycle
TGF-β: Transforming growth factor beta
TLRs: TOLL-like receptors
TME: The tumor microenvironment
TNBC: Triple-negative breast cancer
Treg: Regulatory T cell
Trp: Tryptophan
UDP-GlcNAc: Uridine diphosphate-N-acetylglucosamine
VEGF: Vascular endothelial growth factor
αKG: α-Ketoglutaric acid

## References

[1] Hinshaw DC, Shevde LA. The tumor microenvironment innately modulates cancer progression. Cancer Res. 2019;79:4557–66. 31350295 10.1158/0008-5472.CAN-18-3962 PMC6744958

[2] Ho WJ, Jaffee EM, Zheng L. The tumour microenvironment in pancreatic cancer—clinical challenges and opportunities. Nat Rev Clin Oncol. 2020;17:527–40. 32398706 10.1038/s41571-020-0363-5 PMC7442729

[3] Martínez-Reyes I, Chandel NS. Cancer metabolism: looking forward. Nat Rev Cancer. 2021;21:669–80. 34272515 10.1038/s41568-021-00378-6

[4] Yang J, Shay C, Saba NF, Teng Y. Cancer metabolism and carcinogenesis. Exp Hematol Oncol. 2024;13:10. 38287402 10.1186/s40164-024-00482-x PMC10826200

[5] Zheng S, Wang W, Shen L, Yao Y, Xia W, Ni C. Tumor battlefield within inflamed, excluded or desert immune phenotypes: the mechanisms and strategies. Exp Hematol Oncol. 2024;13:80. 39107856 10.1186/s40164-024-00543-1 PMC11301948

[6] Yoon H, Shaw JL, Haigis MC, Greka A. Lipid metabolism in sickness and in health: emerging regulators of lipotoxicity. Mol Cell. 2021;81:3708–30. 34547235 10.1016/j.molcel.2021.08.027 PMC8620413

[7] Stefan N, Schulze MB. Metabolic health and cardiometabolic risk clusters: implications for prediction, prevention, and treatment. Lancet Diabetes Endocrinol. 2023;11:426–40. 37156256 10.1016/S2213-8587(23)00086-4

[8] Fan Y, Pedersen O. Gut microbiota in human metabolic health and disease. Nat Rev Microbiol. 2021;19:55–71. 32887946 10.1038/s41579-020-0433-9

[9] Luo Y, Li Y, Fang M, Wang S, Shao L, Zou R, et al. Multi-omics synergy in oncology: unraveling the complex interplay of radiomic, genoproteomic, and pathological data. Intell Oncol. 2025;1:17–30.

[10] Gu J, Zhang J, Zeng S, Zhang W, Xia R, Wang X, et al. Artificial intelligence in tumor drug resistance: mechanisms and treatment prospects. Intell Oncol. 2025;1:73–88.

[11] Lin J, Rao D, Zhang M, Gao Q. Metabolic reprogramming in the tumor microenvironment of liver cancer. J Hematol OncolJ Hematol Oncol. 2024;17:6. 38297372 10.1186/s13045-024-01527-8 PMC10832230

[12] Saha T, Dash C, Jayabalan R, Khiste S, Kulkarni A, Kurmi K, et al. Intercellular nanotubes mediate mitochondrial trafficking between cancer and immune cells. Nat Nanotechnol. 2022;17:98–106. 34795441 10.1038/s41565-021-01000-4 PMC10071558

[13] Vallet N, Le Grand S, Bondeelle L, Hoareau B, Corneau A, Bouteiller D, et al. Azithromycin promotes relapse by disrupting immune and metabolic networks after allogeneic stem cell transplantation. Blood. 2022;140:2500–13. 35984904 10.1182/blood.2022016926

[14] Liu S, Zhang H, Li Y, Zhang Y, Bian Y, Zeng Y, et al. S100A4 enhances protumor macrophage polarization by control of PPAR-γ-dependent induction of fatty acid oxidation. J Immunother Cancer. 2021;9: e002548. 34145030 10.1136/jitc-2021-002548 PMC8215236

[15] Bray F, Ferlay J, Soerjomataram I, Siegel RL, Torre LA, Jemal A. Global cancer statistics 2018: GLOBOCAN estimates of incidence and mortality worldwide for 36 cancers in 185 countries. CA Cancer J Clin. 2018;68:394–424. 30207593 10.3322/caac.21492

[16] Dobosz P, Dzieciątkowski T. The intriguing history of cancer immunotherapy. Front Immunol. 2019;10:2965. 31921205 10.3389/fimmu.2019.02965 PMC6928196

[17] Jia Q, Wang A, Yuan Y, Zhu B, Long H. Heterogeneity of the tumor immune microenvironment and its clinical relevance. Exp Hematol Oncol. 2022;11:24. 35461288 10.1186/s40164-022-00277-y PMC9034473

[18] Dinarello CA. Historical insights into cytokines. Eur J Immunol. 2007;37(Suppl 1):S34-45. 17972343 10.1002/eji.200737772 PMC3140102

[19] Fendt S-M. 100 years of the warburg effect: a cancer metabolism endeavor. Cell. 2024;187:3824–8. 39059359 10.1016/j.cell.2024.06.026

[20] Zhang Y, Zhang Z. The history and advances in cancer immunotherapy: understanding the characteristics of tumor-infiltrating immune cells and their therapeutic implications. Cell Mol Immunol. 2020;17:807–21. 32612154 10.1038/s41423-020-0488-6 PMC7395159

[21] Peng Y, Wang Y, Zhou C, Mei W, Zeng C. PI3K/akt/mTOR pathway and its role in cancer therapeutics: are we making headway? Front Oncol. 2022;12:819128. 35402264 10.3389/fonc.2022.819128 PMC8987494

[22] Bhattarai D, Xu X, Lee K. Hypoxia-inducible factor-1 (HIF-1) inhibitors from the last decade (2007 to 2016): a “structure-activity relationship” perspective. Med Res Rev. 2018;38:1404–42. 29278273 10.1002/med.21477

[23] Angelin A, Gil-de-Gómez L, Dahiya S, Jiao J, Guo L, Levine MH, et al. Foxp3 reprograms T cell metabolism to function in low-glucose, high-lactate environments. Cell Metab. 2017;25:1282-1293.e7. 28416194 10.1016/j.cmet.2016.12.018 PMC5462872

[24] Zhang D, Li AM, Hu G, Huang M, Yang F, Zhang L, et al. PHGDH-mediated endothelial metabolism drives glioblastoma resistance to chimeric antigen receptor T cell immunotherapy. Cell Metab. 2023;35:517-534.e8. 36804058 10.1016/j.cmet.2023.01.010 PMC10088869

[25] Huang RH, Wang LX, He J, Gao W. Application and prospects of single cell sequencing in tumors. Biomark Res. 2021;9:88. 34895349 10.1186/s40364-021-00336-2 PMC8665603

[26] Li Q, Geng S, Luo H, Wang W, Mo Y-Q, Luo Q, et al. Signaling pathways involved in colorectal cancer: pathogenesis and targeted therapy. Signal Transduct Target Ther. 2024;9:266. 39370455 10.1038/s41392-024-01953-7 PMC11456611

[27] Gonzalez H, Hagerling C, Werb Z. Roles of the immune system in cancer: from tumor initiation to metastatic progression. Genes Dev. 2018;32:1267–84. 30275043 10.1101/gad.314617.118 PMC6169832

[28] Luo H, Li Q, Wang R-T, Zhang L, Zhang W, Deng M-S, et al. Downregulation of pro-surfactant protein B contributes to the recurrence of early-stage non-small cell lung cancer by activating PGK1-mediated akt signaling. Exp Hematol Oncol. 2023;12:94. 37946295 10.1186/s40164-023-00455-6 PMC10633994

[29] Luo H, Hu B, Gu X-R, Chen J, Fan X-Q, Zhang W, et al. The miR-23a/27a/24—2 cluster drives immune evasion and resistance to PD-1/PD-L1 blockade in non-small cell lung cancer. Mol Cancer. 2024;23:285. 39736629 10.1186/s12943-024-02201-w PMC11686834

[30] Li Q, Luo H, Dai F-Q, Wang R-T, Fan X-Q, Luo Y-Y, et al. SAMD9 promotes postoperative recurrence of esophageal squamous cell carcinoma by stimulating MYH9-mediated GSK3β/β-catenin signaling. Adv Sci Weinh Baden-Wurtt Ger. 2023;10: e2203573. 10.1002/advs.202203573 PMC10104667 36757050

[31] Massonneau J, Ouellet C, Lucien F, Dubois CM, Tyler J, Boissonneault G. Suboptimal extracellular pH values alter DNA damage response to induced double-strand breaks. FEBS Open Bio. 2018;8:416–25. 29511618 10.1002/2211-5463.12384 PMC5832969

[32] Guo L, Kong D, Liu J, Zhan L, Luo L, Zheng W, et al. Breast cancer heterogeneity and its implication in personalized precision therapy. Exp Hematol Oncol. 2023;12:3. 36624542 10.1186/s40164-022-00363-1 PMC9830930

[33] Sgarra R, Battista S, Cerchia L, Manfioletti G, Fedele M. Mechanism of action of lactic acid on histones in cancer. Antioxid Redox Signal. 2024;40:236–49. 36851901 10.1089/ars.2022.0190

[34] Yin Y, Feng W, Chen J, Chen X, Wang G, Wang S, et al. Immunosuppressive tumor microenvironment in the progression, metastasis, and therapy of hepatocellular carcinoma: from bench to bedside. Exp Hematol Oncol. 2024;13:72. 39085965 10.1186/s40164-024-00539-x PMC11292955

[35] Li Z, Zhang H. Reprogramming of glucose, fatty acid and amino acid metabolism for cancer progression. Cell Mol Life Sci. 2016;73:377–92. 26499846 10.1007/s00018-015-2070-4 PMC11108301

[36] Enríquez JA, Mittelbrunn M. Warburg effect reshapes tumor immunogenicity. Cancer Res. 2024;84:2043–5. 38657107 10.1158/0008-5472.CAN-24-1304

[37] Lu J, Tan M, Cai Q. The Warburg effect in tumor progression: mitochondrial oxidative metabolism as an anti-metastasis mechanism. Cancer Lett. 2015;356:156–64. 24732809 10.1016/j.canlet.2014.04.001 PMC4195816

[38] Birts CN, Banerjee A, Darley M, Dunlop CR, Nelson S, Nijjar SK, et al. p53 is regulated by aerobic glycolysis in cancer cells by the CtBP family of NADH-dependent transcriptional regulators. Sci Signal. 2020;13:eaau9529. 32371497 10.1126/scisignal.aau9529 PMC7244340

[39] Tarhonska K, Janasik B, Roszak J, Kowalczyk K, Lesicka M, Reszka E, et al. Environmental exposure to cadmium in breast cancer—association with the Warburg effect and sensitivity to tamoxifen. Biomed Pharmacother. 2023;161:114435. 36842352 10.1016/j.biopha.2023.114435

[40] Zhang Z, Li X, Yang F, Chen C, Liu P, Ren Y, et al. DHHC9-mediated GLUT1 S-palmitoylation promotes glioblastoma glycolysis and tumorigenesis. Nat Commun. 2021;12:5872. 34620861 10.1038/s41467-021-26180-4 PMC8497546

[41] Wu Q, Ba-Alawi W, Deblois G, Cruickshank J, Duan S, Lima-Fernandes E, et al. GLUT1 inhibition blocks growth of RB1-positive triple negative breast cancer. Nat Commun. 2020;11:4205. 32826891 10.1038/s41467-020-18020-8 PMC7442809

[42] Wang K, Dai X, Yu A, Feng C, Liu K, Huang L. Peptide-based PROTAC degrader of FOXM1 suppresses cancer and decreases GLUT1 and PD-L1 expression. J Exp Clin Cancer Res. 2022;41:289. 36171633 10.1186/s13046-022-02483-2 PMC9520815

[43] Zhao J, Sun R, Zhi L, Guo D, Ling S, Liang X, et al. METTL4 and METTL5 as biomarkers for recurrence-free survival in hepatocellular carcinoma patients. Future Oncol Lond Engl. 2024;21:331. 10.1080/14796694.2024.2442296 PMC11792863 39706798

[44] Xia P, Zhang H, Lu H, Xu K, Jiang X, Jiang Y, et al. METTL5 stabilizes c-Myc by facilitating USP5 translation to reprogram glucose metabolism and promote hepatocellular carcinoma progression. Cancer Commun. 2023;43:338–64. 10.1002/cac2.12403 PMC10009668 36602428

[45] Cao M, Isaac R, Yan W, Ruan X, Jiang L, Wan Y, et al. Cancer-cell-secreted extracellular vesicles suppress insulin secretion through miR-122 to impair systemic glucose homeostasis and contribute to tumour growth. Nat Cell Biol. 2022;24:954–67. 35637408 10.1038/s41556-022-00919-7 PMC9233030

[46] Huang Y, Chen Z, Lu T, Bi G, Li M, Liang J, et al. HIF-1α switches the functionality of TGF-β signaling via changing the partners of smads to drive glucose metabolic reprogramming in non-small cell lung cancer. J Exp Clin Cancer Res. 2021;40:398. 34930376 10.1186/s13046-021-02188-y PMC8690885

[47] Wei Z, Xia J, Li J, Cai J, Shan J, Zhang C, et al. SIRT1 promotes glucolipid metabolic conversion to facilitate tumor development in colorectal carcinoma. Int J Biol Sci. 2023;19:1925–40. 37063423 10.7150/ijbs.76704 PMC10092765

[48] Mates JM, Segura JA, Martin-Rufian M, Campos-Sandoval JA, Alonso FJ, Marquez J. Glutaminase isoenzymes as key regulators in metabolic and oxidative stress against cancer. Curr Mol Med. 2013;13:514–34. 22934847 10.2174/1566524011313040005

[49] Ma G, Zhang Z, Li P, Zhang Z, Zeng M, Liang Z, et al. Reprogramming of glutamine metabolism and its impact on immune response in the tumor microenvironment. Cell Commun Signal. 2022;20:114. 35897036 10.1186/s12964-022-00909-0 PMC9327201

[50] Koppula P, Zhuang L, Gan B. Cystine transporter SLC7A11/xCT in cancer: ferroptosis, nutrient dependency, and cancer therapy. Protein Cell. 2021;12:599–620. 33000412 10.1007/s13238-020-00789-5 PMC8310547

[51] Yoo HC, Park SJ, Nam M, Kang J, Kim K, Yeo JH, et al. A variant of SLC1A5 is a mitochondrial glutamine transporter for metabolic reprogramming in cancer cells. Cell Metab. 2020;31:267-283.e12. 31866442 10.1016/j.cmet.2019.11.020

[52] Tong Y, Guo D, Lin S-H, Liang J, Yang D, Ma C, et al. SUCLA2-coupled regulation of GLS succinylation and activity counteracts oxidative stress in tumor cells. Mol Cell. 2021;81:2303-2316.e8. 33991485 10.1016/j.molcel.2021.04.002

[53] Li Y, Li B, Xu Y, Qian L, Xu T, Meng G, et al. GOT2 silencing promotes reprogramming of glutamine metabolism and sensitizes hepatocellular carcinoma to glutaminase inhibitors. Cancer Res. 2022;82:3223–35. 35895805 10.1158/0008-5472.CAN-22-0042

[54] Labuschagne CF, van den Broek NJF, Mackay GM, Vousden KH, Maddocks ODK. Serine, but not glycine, supports one-carbon metabolism and proliferation of cancer cells. Cell Rep. 2014;7:1248–58. 24813884 10.1016/j.celrep.2014.04.045

[55] Ducker GS, Chen L, Morscher RJ, Ghergurovich JM, Esposito M, Teng X, et al. Reversal of cytosolic one-carbon flux compensates for loss of the mitochondrial folate pathway. Cell Metab. 2016;23:1140–53. 27211901 10.1016/j.cmet.2016.04.016 PMC4909566

[56] Geeraerts SL, Heylen E, De Keersmaecker K, Kampen KR. The ins and outs of serine and glycine metabolism in cancer. Nat Metab. 2021;3:131–41. 33510397 10.1038/s42255-020-00329-9

[57] Geng P, Qin W, Xu G. Proline metabolism in cancer. Amino Acids. 2021;53:1769–77. 34390414 10.1007/s00726-021-03060-1

[58] Niu F, Yu Y, Li Z, Ren Y, Li Z, Ye Q, et al. Arginase: an emerging and promising therapeutic target for cancer treatment. Biomed Pharmacother. 2022;149:112840. 35316752 10.1016/j.biopha.2022.112840

[59] Pham T-N, Liagre B, Girard-Thernier C, Demougeot C. Research of novel anticancer agents targeting arginase inhibition. Drug Discov Today. 2018;23:871–8. 29391126 10.1016/j.drudis.2018.01.046

[60] Bedford MT, Clarke SG. Protein arginine methylation in mammals: who, what, and why. Mol Cell. 2009;33:1–13. 19150423 10.1016/j.molcel.2008.12.013 PMC3372459

[61] Bryant J-P, Heiss J, Banasavadi-Siddegowda YK. Arginine methylation in brain tumors: tumor biology and therapeutic strategies. Cells. 2021;10:124. 33440687 10.3390/cells10010124 PMC7827394

[62] Koundouros N, Poulogiannis G. Reprogramming of fatty acid metabolism in cancer. Br J Cancer. 2020;122:4–22. 31819192 10.1038/s41416-019-0650-z PMC6964678

[63] Yang P, Qin H, Li Y, Xiao A, Zheng E, Zeng H, et al. CD36-mediated metabolic crosstalk between tumor cells and macrophages affects liver metastasis. Nat Commun. 2022;13:5782. 36184646 10.1038/s41467-022-33349-y PMC9527239

[64] Nelson ER, Wardell SE, Jasper JS, Park S, Suchindran S, Howe MK, et al. 27-hydroxycholesterol links hypercholesterolemia and breast cancer pathophysiology. Science. 2013;342:1094–8. 24288332 10.1126/science.1241908 PMC3899689

[65] He W, Wang M, Zhang X, Wang Y, Zhao D, Li W, et al. Estrogen induces LCAT to maintain cholesterol homeostasis and suppress hepatocellular carcinoma development. Cancer Res. 2024;84:2417–31. 38718297 10.1158/0008-5472.CAN-23-3966

[66] Chavarro JE, Kenfield SA, Stampfer MJ, Loda M, Campos H, Sesso HD, et al. Blood levels of saturated and monounsaturated fatty acids as markers of de novo lipogenesis and risk of prostate cancer. Am J Epidemiol. 2013;178:1246–55. 23989197 10.1093/aje/kwt136 PMC3792734

[67] Snaebjornsson MT, Janaki-Raman S, Schulze A. Greasing the wheels of the cancer machine: the role of lipid metabolism in cancer. Cell Metab. 2020;31:62–76. 31813823 10.1016/j.cmet.2019.11.010

[68] Maan M, Peters JM, Dutta M, Patterson AD. Lipid metabolism and lipophagy in cancer. Biochem Biophys Res Commun. 2018;504:582–9. 29438712 10.1016/j.bbrc.2018.02.097 PMC6086774

[69] Kaderi MA, Kanduri M, Buhl AM, Sevov M, Cahill N, Gunnarsson R, et al. LPL is the strongest prognostic factor in a comparative analysis of RNA-based markers in early chronic lymphocytic leukemia. Haematologica. 2011;96:1153–60. 21508119 10.3324/haematol.2010.039396 PMC3148909

[70] Bian X, Liu R, Meng Y, Xing D, Xu D, Lu Z. Lipid metabolism and cancer. J Exp Med. 2021;218: e20201606. 33601415 10.1084/jem.20201606 PMC7754673

[71] Nguyen TTT, Zhang Y, Shang E, Shu C, Torrini C, Zhao J, et al. HDAC inhibitors elicit metabolic reprogramming by targeting super-enhancers in glioblastoma models. J Clin Invest. 2020;130:3699–716. 32315286 10.1172/JCI129049 PMC7324177

[72] Geng F, Zhong Y, Su H, Lefai E, Magaki S, Cloughesy TF, et al. SREBP-1 upregulates lipophagy to maintain cholesterol homeostasis in brain tumor cells. Cell Rep. 2023;42:112790. 37436895 10.1016/j.celrep.2023.112790 PMC10528745

[73] Zhu L, Zhu X, Wu Y. Effects of glucose metabolism, lipid metabolism, and glutamine metabolism on tumor microenvironment and clinical implications. Biomolecules. 2022;12:580. 35454171 10.3390/biom12040580 PMC9028125

[74] Chen Y-J, Mahieu NG, Huang X, Singh M, Crawford PA, Johnson SL, et al. Lactate metabolism is associated with mammalian mitochondria. Nat Chem Biol. 2016;12:937–43. 27618187 10.1038/nchembio.2172 PMC5069139

[75] Guertin DA, Wellen KE. Acetyl-CoA metabolism in cancer. Nat Rev Cancer. 2023;23:156–72. 36658431 10.1038/s41568-022-00543-5 PMC11137663

[76] Gao X, Lin S-H, Ren F, Li J-T, Chen J-J, Yao C-B, et al. Acetate functions as an epigenetic metabolite to promote lipid synthesis under hypoxia. Nat Commun. 2016;7:11960. 27357947 10.1038/ncomms11960 PMC4931325

[77] Gu J, Zhou J, Chen Q, Xu X, Gao J, Li X, et al. Tumor metabolite lactate promotes tumorigenesis by modulating MOESIN lactylation and enhancing TGF-β signaling in regulatory T cells. Cell Rep. 2022;39:110986. 35732125 10.1016/j.celrep.2022.110986

[78] Lv H, Lv G, Chen C, Zong Q, Jiang G, Ye D, et al. NAD+ metabolism maintains inducible PD-L1 expression to drive tumor immune evasion. Cell Metab. 2021;33:110-127.e5. 33171124 10.1016/j.cmet.2020.10.021

[79] Shapouri-Moghaddam A, Mohammadian S, Vazini H, Taghadosi M, Esmaeili S, Mardani F, et al. Macrophage plasticity, polarization, and function in health and disease. J Cell Physiol. 2018;233:6425–40. 29319160 10.1002/jcp.26429 PMC13482560

[80] Guan F, Wang R, Yi Z, Luo P, Liu W, Xie Y, et al. Tissue macrophages: origin, heterogenity, biological functions, diseases and therapeutic targets. Signal Transduct Target Ther. 2025;10:1–67. 40055311 10.1038/s41392-025-02124-y PMC11889221

[81] Li M, Yang Y, Xiong L, Jiang P, Wang J, Li C. Metabolism, metabolites, and macrophages in cancer. J Hematol OncolJ Hematol Oncol. 2023;16:80. 37491279 10.1186/s13045-023-01478-6 PMC10367370

[82] Wang H, Yung MM, Xuan Y, Chen F, Chan W, Siu MK, et al. Polyunsaturated fatty acids promote M2-like TAM deposition via dampening RhoA-YAP1 signaling in the ovarian cancer microenvironment. Exp Hematol Oncol. 2024;13:90. 39198883 10.1186/s40164-024-00558-8 PMC11360340

[83] Toledo B, Zhu Chen L, Paniagua-Sancho M, Marchal JA, Perán M, Giovannetti E. Deciphering the performance of macrophages in tumour microenvironment: a call for precision immunotherapy. J Hematol OncolJ Hematol Oncol. 2024;17:44. 38863020 10.1186/s13045-024-01559-0 PMC11167803

[84] Cao M, Wang Z, Lan W, Xiang B, Liao W, Zhou J, et al. The roles of tissue resident macrophages in health and cancer. Exp Hematol Oncol. 2024;13:3. 38229178 10.1186/s40164-023-00469-0 PMC10790434

[85] Xiang X, Wang J, Lu D, Xu X. Targeting tumor-associated macrophages to synergize tumor immunotherapy. Signal Transduct Target Ther. 2021;6:75. 33619259 10.1038/s41392-021-00484-9 PMC7900181

[86] Vitale I, Manic G, Coussens LM, Kroemer G, Galluzzi L. Macrophages and metabolism in the tumor microenvironment. Cell Metab. 2019;30:36–50. 31269428 10.1016/j.cmet.2019.06.001

[87] Kelly B, O’Neill LA. Metabolic reprogramming in macrophages and dendritic cells in innate immunity. Cell Res. 2015;25:771–84. 26045163 10.1038/cr.2015.68 PMC4493277

[88] Wang Y, Wang D, Yang L, Zhang Y. Metabolic reprogramming in the immunosuppression of tumor-associated macrophages. Chin Med J. 2022;135:2405–16. 36385099 10.1097/CM9.0000000000002426 PMC9945195

[89] Qing J, Zhang Z, Novák P, Zhao G, Yin K. Mitochondrial metabolism in regulating macrophage polarization: an emerging regulator of metabolic inflammatory diseases. Acta Biochim Biophys Sin. 2020;52:917–26. 32785581 10.1093/abbs/gmaa081

[90] Palmieri EM, Menga A, Martín-Pérez R, Quinto A, Riera-Domingo C, De Tullio G, et al. Pharmacologic or genetic targeting of glutamine synthetase skews macrophages toward an M1-like phenotype and inhibits tumor metastasis. Cell Rep. 2017;20:1654–66. 28813676 10.1016/j.celrep.2017.07.054 PMC5575233

[91] Tan AC. Targeting the PI3K/akt/mTOR pathway in non-small cell lung cancer (NSCLC). Thorac Cancer. 2020;11:511–8. 31989769 10.1111/1759-7714.13328 PMC7049515

[92] Poczobutt JM, Gijon M, Amin J, Hanson D, Li H, Walker D, et al. Eicosanoid profiling in an orthotopic model of lung cancer progression by mass spectrometry demonstrates selective production of leukotrienes by inflammatory cells of the microenvironment. PLoS ONE. 2013;8: e79633. 24244531 10.1371/journal.pone.0079633 PMC3823604

[93] Colby JK, Jaoude J, Liu F, Shureiqi I. Oxygenated lipid signaling in tumor-associated macrophages—focus on colon cancer. Cancer Metastasis Rev. 2018;37:289–315. 29934822 10.1007/s10555-018-9743-z

[94] Eruslanov E, Daurkin I, Vieweg J, Daaka Y, Kusmartsev S. Aberrant PGE2 metabolism in bladder tumor microenvironment promotes immunosuppressive phenotype of tumor-infiltrating myeloid cells. Int Immunopharmacol. 2011;11:848–55. 21315786 10.1016/j.intimp.2011.01.033 PMC3241976

[95] Leone RD, Powell JD. Metabolism of immune cells in cancer. Nat Rev Cancer. 2020;20:516–31. 32632251 10.1038/s41568-020-0273-y PMC8041116

[96] Praharaj M, Shen F, Lee AJ, Zhao L, Nirschl TR, Theodros D, et al. Metabolic reprogramming of tumor-associated macrophages using glutamine antagonist JHU083 drives tumor immunity in myeloid-rich prostate and bladder cancers. Cancer Immunol Res. 2024;12:854–75. 38701369 10.1158/2326-6066.CIR-23-1105 PMC11217738

[97] Wang S, Liu R, Yu Q, Dong L, Bi Y, Liu G. Metabolic reprogramming of macrophages during infections and cancer. Cancer Lett. 2019;452:14–22. 30905817 10.1016/j.canlet.2019.03.015

[98] Zhang Q, Song Q, Liu S, Xu Y, Gao D, Lu P, et al. Integrated transcriptomic and metabolomic analysis reveals the metabolic programming of GM-CSF- and M-CSF- differentiated mouse macrophages. Front Immunol. 2023;14:1230772. 37818352 10.3389/fimmu.2023.1230772 PMC10560851

[99] Chen Z-Q, Zuo X-L, Cai J, Zhang Y, Han G-Y, Zhang L, et al. Hypoxia-associated circPRDM4 promotes immune escape via HIF-1α regulation of PD-L1 in hepatocellular carcinoma. Exp Hematol Oncol. 2023;12:17. 36747292 10.1186/s40164-023-00378-2 PMC9903500

[100] Shi Z, Hu C, Zheng X, Sun C, Li Q. Feedback loop between hypoxia and energy metabolic reprogramming aggravates the radioresistance of cancer cells. Exp Hematol Oncol. 2024;13:55. 38778409 10.1186/s40164-024-00519-1 PMC11110349

[101] Guo D, Tong Y, Jiang X, Meng Y, Jiang H, Du L, et al. Aerobic glycolysis promotes tumor immune evasion by hexokinase2-mediated phosphorylation of IκBα. Cell Metab. 2022;34:1312-1324.e6. 36007522 10.1016/j.cmet.2022.08.002

[102] Jiang H, Wei H, Wang H, Wang Z, Li J, Ou Y, et al. Zeb1-induced metabolic reprogramming of glycolysis is essential for macrophage polarization in breast cancer. Cell Death Dis. 2022;13:206. 35246504 10.1038/s41419-022-04632-z PMC8897397

[103] Xu B, Liu Y, Li N, Geng Q. Lactate and lactylation in macrophage metabolic reprogramming: current progress and outstanding issues. Front Immunol. 2024;15:1395786. 38835758 10.3389/fimmu.2024.1395786 PMC11148263

[104] Viola A, Munari F, Sánchez-Rodríguez R, Scolaro T, Castegna A. The metabolic signature of macrophage responses. Front Immunol. 2019;10:1462. 31333642 10.3389/fimmu.2019.01462 PMC6618143

[105] Hartley GP, Chow L, Ammons DT, Wheat WH, Dow SW. Programmed cell death ligand 1 (PD-L1) signaling regulates macrophage proliferation and activation. Cancer Immunol Res. 2018;6:1260–73. 30012633 10.1158/2326-6066.CIR-17-0537

[106] Maldonado LAG, Nascimento CR, Rodrigues Fernandes NA, Silva ALP, D’Silva NJ, Rossa C. Influence of tumor cell-derived TGF-β on macrophage phenotype and macrophage-mediated tumor cell invasion. Int J Biochem Cell Biol. 2022;153:106330. 36343916 10.1016/j.biocel.2022.106330

[107] Wu H, Han Y, Rodriguez Sillke Y, Deng H, Siddiqui S, Treese C, et al. Lipid droplet-dependent fatty acid metabolism controls the immune suppressive phenotype of tumor-associated macrophages. EMBO Mol Med. 2019;11: e10698. 31602788 10.15252/emmm.201910698 PMC6835560

[108] Huang SC-C, Everts B, Ivanova Y, O’Sullivan D, Nascimento M, Smith AM, et al. Cell-intrinsic lysosomal lipolysis is essential for alternative activation of macrophages. Nat Immunol. 2014;15:846–55. 25086775 10.1038/ni.2956 PMC4139419

[109] Su P, Wang Q, Bi E, Ma X, Liu L, Yang M, et al. Enhanced lipid accumulation and metabolism are required for the differentiation and activation of tumor-associated macrophages. Cancer Res. 2020;80:1438–50. 32015091 10.1158/0008-5472.CAN-19-2994 PMC7127942

[110] Mojsilovic SS, Mojsilovic S, Villar VH, Santibanez JF. The metabolic features of tumor-associated macrophages: opportunities for immunotherapy? Anal Cell Pathol Amst. 2021;2021:5523055. 34476174 10.1155/2021/5523055 PMC8407977

[111] Xiang W, Shi R, Kang X, Zhang X, Chen P, Zhang L, et al. Monoacylglycerol lipase regulates cannabinoid receptor 2-dependent macrophage activation and cancer progression. Nat Commun. 2018;9:2574. 29968710 10.1038/s41467-018-04999-8 PMC6030061

[112] Wu L, Zhang X, Zheng L, Zhao H, Yan G, Zhang Q, et al. RIPK3 orchestrates fatty acid metabolism in tumor-associated macrophages and hepatocarcinogenesis. Cancer Immunol Res. 2020;8:710–21. 32122992 10.1158/2326-6066.CIR-19-0261

[113] Hoppstädter J, Dembek A, Höring M, Schymik HS, Dahlem C, Sultan A, et al. Dysregulation of cholesterol homeostasis in human lung cancer tissue and tumour-associated macrophages. EBioMedicine. 2021;72:103578. 34571364 10.1016/j.ebiom.2021.103578 PMC8479395

[114] Goossens P, Rodriguez-Vita J, Etzerodt A, Masse M, Rastoin O, Gouirand V, et al. Membrane cholesterol efflux drives tumor-associated macrophage reprogramming and tumor progression. Cell Metab. 2019;29:1376-1389.e4. 30930171 10.1016/j.cmet.2019.02.016

[115] Liu Y, Xu R, Gu H, Zhang E, Qu J, Cao W, et al. Metabolic reprogramming in macrophage responses. Biomark Res. 2021;9:1. 33407885 10.1186/s40364-020-00251-y PMC7786975

[116] Liu P-S, Wang H, Li X, Chao T, Teav T, Christen S, et al. α-ketoglutarate orchestrates macrophage activation through metabolic and epigenetic reprogramming. Nat Immunol. 2017;18:985–94. 28714978 10.1038/ni.3796

[117] Choi J, Stradmann-Bellinghausen B, Yakubov E, Savaskan NE, Régnier-Vigouroux A. Glioblastoma cells induce differential glutamatergic gene expressions in human tumor-associated microglia/macrophages and monocyte-derived macrophages. Cancer Biol Ther. 2015;16:1205–13. 26047211 10.1080/15384047.2015.1056406 PMC4623498

[118] Mills C. M1 and M2 macrophages: oracles of health and disease. Crit Rev Immunol. 2012;32:463–88. 23428224 10.1615/critrevimmunol.v32.i6.10

[119] Kieler M, Hofmann M, Schabbauer G. More than just protein building blocks: how amino acids and related metabolic pathways fuel macrophage polarization. FEBS J. 2021;288:3694–714. 33460504 10.1111/febs.15715 PMC8359336

[120] Dussold C, Zilinger K, Turunen J, Heimberger AB, Miska J. Modulation of macrophage metabolism as an emerging immunotherapy strategy for cancer. J Clin Invest. 2024;134: e175445. 38226622 10.1172/JCI175445 PMC10786697

[121] Seo S-K, Kwon B. Immune regulation through tryptophan metabolism. Exp Mol Med. 2023;55:1371–9. 37394584 10.1038/s12276-023-01028-7 PMC10394086

[122] Xue C, Li G, Zheng Q, Gu X, Shi Q, Su Y, et al. Tryptophan metabolism in health and disease. Cell Metab. 2023;35:1304–26. 37352864 10.1016/j.cmet.2023.06.004

[123] Wang X-F, Wang H-S, Wang H, Zhang F, Wang K-F, Guo Q, et al. The role of indoleamine 2,3-dioxygenase (IDO) in immune tolerance: focus on macrophage polarization of THP-1 cells. Cell Immunol. 2014;289:42–8. 24721110 10.1016/j.cellimm.2014.02.005

[124] Shan X, Hu P, Ni L, Shen L, Zhang Y, Ji Z, et al. Serine metabolism orchestrates macrophage polarization by regulating the IGF1–p38 axis. Cell Mol Immunol. 2022;19:1263–78. 36180780 10.1038/s41423-022-00925-7 PMC9622887

[125] Fridlender ZG, Sun J, Kim S, Kapoor V, Cheng G, Ling L, et al. Polarization of tumor-associated neutrophil phenotype by TGF-β: “N1” versus “N2” TAN. Cancer Cell. 2009;16:183–94. 19732719 10.1016/j.ccr.2009.06.017 PMC2754404

[126] Rogers T, DeBerardinis RJ. Metabolic plasticity of neutrophils: relevance to pathogen responses and cancer. Trends Cancer. 2021;7:700–13. 34023325 10.1016/j.trecan.2021.04.007 PMC9270875

[127] Bodac A, Meylan E. Neutrophil metabolism in the cancer context. Semin Immunol. 2021;57:101583. 34963565 10.1016/j.smim.2021.101583

[128] Li D-D, Jawale CV, Zhou C, Lin L, Trevejo-Nunez GJ, Rahman SA, et al. Fungal sensing enhances neutrophil metabolic fitness by regulating antifungal Glut1 activity. Cell Host Microbe. 2022;30:530-544.e6. 35316647 10.1016/j.chom.2022.02.017 PMC9026661

[129] Kalafati L, Kourtzelis I, Schulte-Schrepping J, Li X, Hatzioannou A, Grinenko T, et al. Innate immune training of granulopoiesis promotes anti-tumor activity. Cell. 2020;183:771-785.e12. 33125892 10.1016/j.cell.2020.09.058 PMC7599076

[130] Casbon A-J, Reynaud D, Park C, Khuc E, Gan DD, Schepers K, et al. Invasive breast cancer reprograms early myeloid differentiation in the bone marrow to generate immunosuppressive neutrophils. Proc Natl Acad Sci U S A. 2015;112:E566–75. 25624500 10.1073/pnas.1424927112 PMC4330753

[131] Azevedo EP, Rochael NC, Guimarães-Costa AB, De Souza-Vieira TS, Ganilho J, Saraiva EM, et al. A metabolic shift toward pentose phosphate pathway is necessary for amyloid fibril- and phorbol 12-myristate 13-acetate-induced neutrophil extracellular trap (NET) formation. J Biol Chem. 2015;290:22174–83. 26198639 10.1074/jbc.M115.640094 PMC4571968

[132] Rodríguez-Espinosa O, Rojas-Espinosa O, Moreno-Altamirano MMB, López-Villegas EO, Sánchez-García FJ. Metabolic requirements for neutrophil extracellular traps formation. Immunology. 2015;145:213–24. 25545227 10.1111/imm.12437 PMC4427386

[133] Rice CM, Davies LC, Subleski JJ, Maio N, Gonzalez-Cotto M, Andrews C, et al. Tumour-elicited neutrophils engage mitochondrial metabolism to circumvent nutrient limitations and maintain immune suppression. Nat Commun. 2018;9:5099. 30504842 10.1038/s41467-018-07505-2 PMC6269473

[134] Hossain F, Al-Khami AA, Wyczechowska D, Hernandez C, Zheng L, Reiss K, et al. Inhibition of fatty acid oxidation modulates immunosuppressive functions of myeloid-derived suppressor cells and enhances cancer therapies. Cancer Immunol Res. 2015;3:1236–47. 26025381 10.1158/2326-6066.CIR-15-0036 PMC4636942

[135] García-Navas R, Gajate C, Mollinedo F. Neutrophils drive endoplasmic reticulum stress-mediated apoptosis in cancer cells through arginase-1 release. Sci Rep. 2021;11:12574. 34131176 10.1038/s41598-021-91947-0 PMC8206108

[136] Ancey P-B, Contat C, Boivin G, Sabatino S, Pascual J, Zangger N, et al. GLUT1 expression in tumor-associated neutrophils promotes lung cancer growth and resistance to radiotherapy. Cancer Res. 2021;81:2345–57. 33753374 10.1158/0008-5472.CAN-20-2870 PMC8137580

[137] Miao P, Sheng S, Sun X, Liu J, Huang G. Lactate dehydrogenase a in cancer: a promising target for diagnosis and therapy: LDHA in cancer. IUBMB Life. 2013;65:904–10. 24265197 10.1002/iub.1216

[138] Wang Y, Xu M, Sun J, Li X, Shi H, Wang X, et al. Glycolytic neutrophils accrued in the spleen compromise anti-tumour T cell immunity in breast cancer. Nat Metab. 2023;5:1408–22. 37563468 10.1038/s42255-023-00853-4

[139] Noman MZ, Desantis G, Janji B, Hasmim M, Karray S, Dessen P, et al. PD-L1 is a novel direct target of HIF-1α, and its blockade under hypoxia enhanced MDSC-mediated T cell activation. J Exp Med. 2014;211:781–90. 24778419 10.1084/jem.20131916 PMC4010891

[140] Patel S, Fu S, Mastio J, Dominguez GA, Purohit A, Kossenkov A, et al. Unique pattern of neutrophil migration and function during tumor progression. Nat Immunol. 2018;19:1236–47. 30323345 10.1038/s41590-018-0229-5 PMC6195445

[141] Fainsod-Levi T, Gershkovitz M, Völs S, Kumar S, Khawaled S, Sagiv JY, et al. Hyperglycemia impairs neutrophil mobilization leading to enhanced metastatic seeding. Cell Rep. 2017;21:2384–92. 29186678 10.1016/j.celrep.2017.11.010

[142] Oberg H-H, Wesch D, Kalyan S, Kabelitz D. Regulatory interactions between neutrophils, tumor cells and T cells. Front Immunol. 2019;10:1690. 31379875 10.3389/fimmu.2019.01690 PMC6657370

[143] Michaeli J, Shaul ME, Mishalian I, Hovav A-H, Levy L, Zolotriov L, et al. Tumor-associated neutrophils induce apoptosis of non-activated CD8 T-cells in a TNFα and NO-dependent mechanism, promoting a tumor-supportive environment. OncoImmunology. 2017;6: e1356965. 29147615 10.1080/2162402X.2017.1356965 PMC5674962

[144] Riffelmacher T, Clarke A, Richter FC, Stranks A, Pandey S, Danielli S, et al. Autophagy-dependent generation of free fatty acids is critical for normal neutrophil differentiation. Immunity. 2017;47:466-480.e5. 28916263 10.1016/j.immuni.2017.08.005 PMC5610174

[145] Lavoie J-PC, Simard M, Kalkan H, Rakotoarivelo V, Huot S, Di Marzo V, et al. Pharmacological evidence that the inhibitory effects of prostaglandin E2 are mediated by the EP2 and EP4 receptors in human neutrophils. J Leukoc Biol. 2024;115:1183–9. 38345417 10.1093/jleuko/qiae029 PMC11135612

[146] Veglia F, Tyurin VA, Blasi M, De Leo A, Kossenkov AV, Donthireddy L, et al. Fatty acid transport protein 2 reprograms neutrophils in cancer. Nature. 2019;569:73–8. 30996346 10.1038/s41586-019-1118-2 PMC6557120

[147] Que H, Fu Q, Lan T, Tian X, Wei X. Tumor-associated neutrophils and neutrophil-targeted cancer therapies. Biochim Biophys Acta BBA Rev Cancer. 2022;1877:188762. 10.1016/j.bbcan.2022.188762 35853517

[148] Shi K, Hou J, Zhang Q, Bi Y, Zeng X, Wang X. Neutrophil-to-high-density-lipoprotein-cholesterol ratio and mortality among patients with hepatocellular carcinoma. Front Nutr. 2023;10:1127913. 37215223 10.3389/fnut.2023.1127913 PMC10198653

[149] Wu C-F, Wang C-C, Tai T-S, Su Y-C. ER stress of cancer cell SCC25 induces LOX-1-expressed immunosuppressive neutrophils. Ann Oncol. 2019;30:xi55.

[150] Furukawa S, Saito H, Inoue T, Matsuda T, Fukatsu K, Han I, et al. Supplemental glutamine augments phagocytosis and reactive oxygen intermediate production by neutrophils and monocytes from postoperative patients in vitro. Nutrition. 2000;16:323–9. 10793298 10.1016/s0899-9007(00)00228-8

[151] Watts ER, Howden AJM, Morrison T, Sadiku P, Hukelmann J, Von Kriegsheim A, et al. Hypoxia drives murine neutrophil protein scavenging to maintain central carbon metabolism. J Clin Invest. 2021;131: e134073. 33822765 10.1172/JCI134073 PMC8121528

[152] Xiong T, He P, Zhou M, Zhong D, Yang T, He W, et al. Glutamate blunts cell-killing effects of neutrophils in tumor microenvironment. Cancer Sci. 2022;113:1955–67. 35363928 10.1111/cas.15355 PMC9207372

[153] Grzywa TM, Sosnowska A, Matryba P, Rydzynska Z, Jasinski M, Nowis D, et al. Myeloid cell-derived arginase in cancer immune response. Front Immunol. 2020;11:938. 32499785 10.3389/fimmu.2020.00938 PMC7242730

[154] Pang Y, Gara SK, Achyut BR, Li Z, Yan HH, Day C-P, et al. TGF-β signaling in myeloid cells is required for tumor metastasis. Cancer Discov. 2013;3:936–51. 23661553 10.1158/2159-8290.CD-12-0527 PMC4678771

[155] Wu Y, Ma J, Yang X, Nan F, Zhang T, Ji S, et al. Neutrophil profiling illuminates anti-tumor antigen-presenting potency. Cell. 2024;187:1422-1439.e24. 38447573 10.1016/j.cell.2024.02.005

[156] Shi J, Li J, Wang H, Li X, Wang Q, Zhao C, et al. Single-cell profiling of tumor-associated neutrophils in advanced non-small cell lung cancer. Lung Cancer Targets Ther. 2023;14:85–99. 10.2147/LCTT.S430967 PMC10676108 38025400

[157] Qiu H, Shao N, Liu J, Zhao J, Chen C, Li Q, et al. Amino acid metabolism in tumor: new shine in the fog? Clin Nutr. 2023;42:1521–30. 37321900 10.1016/j.clnu.2023.06.011

[158] Balan S, Arnold-Schrauf C, Abbas A, Couespel N, Savoret J, Imperatore F, et al. Large-scale human dendritic cell differentiation revealing notch-dependent lineage bifurcation and heterogeneity. Cell Rep. 2018;24:1902-1915.e6. 30110645 10.1016/j.celrep.2018.07.033 PMC6113934

[159] Krawczyk CM, Holowka T, Sun J, Blagih J, Amiel E, DeBerardinis RJ, et al. Toll-like receptor–induced changes in glycolytic metabolism regulate dendritic cell activation. Blood. 2010;115:4742–9. 20351312 10.1182/blood-2009-10-249540 PMC2890190

[160] Yi M, Niu M, Wu Y, Ge H, Jiao D, Zhu S, et al. Combination of oral STING agonist MSA-2 and anti-TGF-β/PD-L1 bispecific antibody YM101: A novel immune cocktail therapy for non-inflamed tumors. J Hematol OncolJ Hematol Oncol. 2022;15:142. 36209176 10.1186/s13045-022-01363-8 PMC9548169

[161] Shen M, Jiang X, Peng Q, Oyang L, Ren Z, Wang J, et al. The cGAS-STING pathway in cancer immunity: mechanisms, challenges, and therapeutic implications. J Hematol OncolJ Hematol Oncol. 2025;18:40. 40188340 10.1186/s13045-025-01691-5 PMC11972543

[162] Huang Q, Wang F, Hao D, Li X, Li X, Lei T, et al. Deciphering tumor-infiltrating dendritic cells in the single-cell era. Exp Hematol Oncol. 2023;12:97. 38012715 10.1186/s40164-023-00459-2 PMC10680280

[163] Oberkampf M, Guillerey C, Mouriès J, Rosenbaum P, Fayolle C, Bobard A, et al. Mitochondrial reactive oxygen species regulate the induction of CD8+ T cells by plasmacytoid dendritic cells. Nat Commun. 2018;9:2241. 29884826 10.1038/s41467-018-04686-8 PMC5993805

[164] Del Prete A, Salvi V, Soriani A, Laffranchi M, Sozio F, Bosisio D, et al. Dendritic cell subsets in cancer immunity and tumor antigen sensing. Cell Mol Immunol. 2023;20:432–47. 36949244 10.1038/s41423-023-00990-6 PMC10203372

[165] Kedia-Mehta N, Finlay DK. Competition for nutrients and its role in controlling immune responses. Nat Commun. 2019;10:2123. 31073180 10.1038/s41467-019-10015-4 PMC6509329

[166] Lundø K, Trauelsen M, Pedersen SF, Schwartz TW. Why warburg works: lactate controls immune evasion through GPR81. Cell Metab. 2020;31:666–8. 32268113 10.1016/j.cmet.2020.03.001

[167] Paardekooper LM, Dingjan I, Linders PTA, Staal AHJ, Cristescu SM, Verberk WCEP, et al. Human monocyte-derived dendritic cells produce millimolar concentrations of ROS in phagosomes per second. Front Immunol. 2019;10:1216. 31191556 10.3389/fimmu.2019.01216 PMC6548834

[168] Hu Z, Teng X-L, Zhang T, Yu X, Ding R, Yi J, et al. SENP3 senses oxidative stress to facilitate STING-dependent dendritic cell antitumor function. Mol Cell. 2021;81:940-952.e5. 33434504 10.1016/j.molcel.2020.12.024

[169] Peng X, He Y, Huang J, Tao Y, Liu S. Metabolism of dendritic cells in tumor microenvironment: for immunotherapy. Front Immunol. 2021;12:613492. 33732237 10.3389/fimmu.2021.613492 PMC7959811

[170] You Z, Chi H. Lipid metabolism in dendritic cell biology. Immunol Rev. 2023;317:137–51. 37172120 10.1111/imr.13215 PMC10523915

[171] Everts B, Amiel E, Huang SC-C, Smith AM, Chang C-H, Lam WY, et al. TLR-driven early glycolytic reprogramming via the kinases TBK1-IKKɛ supports the anabolic demands of dendritic cell activation. Nat Immunol. 2014;15:323–32. 24562310 10.1038/ni.2833 PMC4358322

[172] Brombacher EC, Everts B. Shaping of dendritic cell function by the metabolic micro-environment. Front Endocrinol. 2020;11:555. 10.3389/fendo.2020.00555 PMC7493661 33013685

[173] Kim S-H, Roszik J, Grimm EA, Ekmekcioglu S. Impact of l-arginine metabolism on immune response and anticancer immunotherapy. Front Oncol. 2018;8:67. 29616189 10.3389/fonc.2018.00067 PMC5864849

[174] Nouwen LV, Everts B. Pathogens MenTORing macrophages and dendritic cells: manipulation of mTOR and cellular metabolism to promote immune escape. Cells. 2020;9:161. 31936570 10.3390/cells9010161 PMC7017145

[175] Zhao Q, Han Y-M, Song P, Liu Z, Yuan Z, Zou M-H. Endothelial cell-specific expression of serine/threonine kinase 11 modulates dendritic cell differentiation. Nat Commun. 2022;13:648. 35115536 10.1038/s41467-022-28316-6 PMC8814147

[176] Dodd KM, Yang J, Shen MH, Sampson JR, Tee AR. mTORC1 drives HIF-1α and VEGF-A signalling via multiple mechanisms involving 4E-BP1, S6K1 and STAT3. Oncogene. 2015;34:2239–50. 24931163 10.1038/onc.2014.164 PMC4172452

[177] Brown TP, Bhattacharjee P, Ramachandran S, Sivaprakasam S, Ristic B, Sikder MOF, et al. The lactate receptor GPR81 promotes breast cancer growth via a paracrine mechanism involving antigen-presenting cells in the tumor microenvironment. Oncogene. 2020;39:3292–304. 32071396 10.1038/s41388-020-1216-5

[178] Caronni N, Simoncello F, Stafetta F, Guarnaccia C, Ruiz-Moreno JS, Opitz B, et al. Downregulation of Membrane trafficking proteins and lactate conditioning determine loss of dendritic cell function in lung cancer. Cancer Res. 2018;78:1685–99. 29363545 10.1158/0008-5472.CAN-17-1307

[179] Hu Z, Yu X, Ding R, Liu B, Gu C, Pan X-W, et al. Glycolysis drives STING signaling to facilitate dendritic cell antitumor function. J Clin Invest. 2023;133: e166031. 36821379 10.1172/JCI166031 PMC10065062

[180] Raychaudhuri D, Bhattacharya R, Sinha BP, Liu CSC, Ghosh AR, Rahaman O, et al. Lactate induces pro-tumor reprogramming in intratumoral plasmacytoid dendritic cells. Front Immunol. 2019;10:1878. 31440253 10.3389/fimmu.2019.01878 PMC6692712

[181] Burgdorf S, Porubsky S, Marx A, Popovic ZV. Cancer acidity and hypertonicity contribute to dysfunction of tumor-associated dendritic cells: potential impact on antigen cross-presentation machinery. Cancers. 2020;12:2403. 32847079 10.3390/cancers12092403 PMC7565485

[182] Liu J, Zhang X, Chen K, Cheng Y, Liu S, Xia M, et al. CCR7 chemokine receptor-inducible lnc-Dpf3 restrains dendritic cell migration by inhibiting HIF-1α-mediated glycolysis. Immunity. 2019;50:600-615.e15. 30824325 10.1016/j.immuni.2019.01.021

[183] Deng H, Yang W, Zhou Z, Tian R, Lin L, Ma Y, et al. Targeted scavenging of extracellular ROS relieves suppressive immunogenic cell death. Nat Commun. 2020;11:4951. 33009382 10.1038/s41467-020-18745-6 PMC7532538

[184] Herber DL, Cao W, Nefedova Y, Novitskiy SV, Nagaraj S, Tyurin VA, et al. Lipid accumulation and dendritic cell dysfunction in cancer. Nat Med. 2010;16:880–6. 20622859 10.1038/nm.2172 PMC2917488

[185] Gardner JK, Mamotte CDS, Patel P, Yeoh TL, Jackaman C, Nelson DJ. Mesothelioma tumor cells modulate dendritic cell lipid content phenotype and function. PLoS ONE. 2015;10: e0123563. 25886502 10.1371/journal.pone.0123563 PMC4401725

[186] Xiao Y, Yang Y, Xiong H, Dong G. The implications of FASN in immune cell biology and related diseases. Cell Death Dis. 2024;15:88. 38272906 10.1038/s41419-024-06463-6 PMC10810964

[187] Gao F, Liu C, Guo J, Sun W, Xian L, Bai D, et al. Radiation-driven lipid accumulation and dendritic cell dysfunction in cancer. Sci Rep. 2015;5:9613. 25923834 10.1038/srep09613 PMC4413852

[188] Cubillos-Ruiz JR, Silberman PC, Rutkowski MR, Chopra S, Perales-Puchalt A, Song M, et al. ER stress sensor XBP1 controls anti-tumor immunity by disrupting dendritic cell homeostasis. Cell. 2015;161:1527–38. 26073941 10.1016/j.cell.2015.05.025 PMC4580135

[189] Zhao F, Xiao C, Evans KS, Theivanthiran T, DeVito N, Holtzhausen A, et al. Paracrine Wnt5a-β-catenin signaling triggers a metabolic program that drives dendritic cell tolerization. Immunity. 2018;48:147-160.e7. 29343435 10.1016/j.immuni.2017.12.004 PMC5777287

[190] Yin X, Zeng W, Wu B, Wang L, Wang Z, Tian H, et al. PPARα inhibition overcomes tumor-derived exosomal lipid-induced dendritic cell dysfunction. Cell Rep. 2020;33:108278. 33086073 10.1016/j.celrep.2020.108278 PMC7771208

[191] Cao W, Ramakrishnan R, Tuyrin VA, Veglia F, Condamine T, Amoscato A, et al. Oxidized lipids block antigen cross-presentation by dendritic cells in cancer. J Immunol. 2014;192:2920–31. 24554775 10.4049/jimmunol.1302801 PMC3998104

[192] Villablanca EJ, Raccosta L, Zhou D, Fontana R, Maggioni D, Negro A, et al. Tumor-mediated liver X receptor-α activation inhibits CC chemokine receptor-7 expression on dendritic cells and dampens antitumor responses. Nat Med. 2010;16:98–105. 20037595 10.1038/nm.2074

[193] Giovanelli P, Sandoval TA, Cubillos-Ruiz JR. Dendritic cell metabolism and function in tumors. Trends Immunol. 2019;40:699–718. 31301952 10.1016/j.it.2019.06.004

[194] Gargaro M, Vacca C, Massari S, Scalisi G, Manni G, Mondanelli G, et al. Engagement of nuclear coactivator 7 by 3-hydroxyanthranilic acid enhances activation of aryl hydrocarbon receptor in immunoregulatory dendritic cells. Front Immunol. 2019;10:1973. 31481962 10.3389/fimmu.2019.01973 PMC6710348

[195] Mondanelli G, Iacono A, Carvalho A, Orabona C, Volpi C, Pallotta MT, et al. Amino acid metabolism as drug target in autoimmune diseases. Autoimmun Rev. 2019;18:334–48. 30797943 10.1016/j.autrev.2019.02.004

[196] Mondanelli G, Bianchi R, Pallotta MT, Orabona C, Albini E, Iacono A, et al. A relay pathway between arginine and tryptophan metabolism confers immunosuppressive properties on dendritic cells. Immunity. 2017;46:233–44. 28214225 10.1016/j.immuni.2017.01.005 PMC5337620

[197] Kheshtchin N, Arab S, Ajami M, Mirzaei R, Ashourpour M, Mousavi N, et al. Inhibition of HIF-1α enhances anti-tumor effects of dendritic cell-based vaccination in a mouse model of breast cancer. Cancer Immunol Immunother. 2016;65:1159–67. 27497816 10.1007/s00262-016-1879-5 PMC11029746

[198] Shang J, Hu S, Wang X. Targeting natural killer cells: from basic biology to clinical application in hematologic malignancies. Exp Hematol Oncol. 2024;13:21. 38396050 10.1186/s40164-024-00481-y PMC10885621

[199] Hou J, Xie S, Gao J, Jiang T, Zhu E, Yang X, et al. NK cell transfer overcomes resistance to PD-(L)1 therapy in aged mice. Exp Hematol Oncol. 2024;13:48. 38725070 10.1186/s40164-024-00511-9 PMC11080179

[200] Shi Y, Hao D, Qian H, Tao Z. Natural killer cell-based cancer immunotherapy: from basics to clinical trials. Exp Hematol Oncol. 2024;13:101. 39415291 10.1186/s40164-024-00561-z PMC11484118

[201] Marçais A, Cherfils-Vicini J, Viant C, Degouve S, Viel S, Fenis A, et al. The metabolic checkpoint kinase mTOR is essential for IL-15 signaling during the development and activation of NK cells. Nat Immunol. 2014;15:749–57. 24973821 10.1038/ni.2936 PMC4110708

[202] O’Brien KL, Finlay DK. Immunometabolism and natural killer cell responses. Nat Rev Immunol. 2019;19:282–90. 30808985 10.1038/s41577-019-0139-2

[203] Miao L, Lu C, Zhang B, Li H, Zhao X, Chen H, et al. Advances in metabolic reprogramming of NK cells in the tumor microenvironment on the impact of NK therapy. J Transl Med. 2024;22:229. 38433193 10.1186/s12967-024-05033-w PMC10909296

[204] Loftus RM, Assmann N, Kedia-Mehta N, O’Brien KL, Garcia A, Gillespie C, et al. Amino acid-dependent cMyc expression is essential for NK cell metabolic and functional responses in mice. Nat Commun. 2018;9:2341. 29904050 10.1038/s41467-018-04719-2 PMC6002377

[205] Lim SA, Moon Y, Shin MH, Kim T-J, Chae S, Yee C, et al. Hypoxia-driven HIF-1α activation reprograms pre-activated NK cells towards highly potent effector phenotypes via ERK/STAT3 pathways. Cancers. 2021;13:1904. 33920906 10.3390/cancers13081904 PMC8071270

[206] Harmon C, Robinson MW, Hand F, Almuaili D, Mentor K, Houlihan DD, et al. Lactate-mediated acidification of tumor microenvironment induces apoptosis of liver-resident NK cells in colorectal liver metastasis. Cancer Immunol Res. 2019;7:335–46. 30563827 10.1158/2326-6066.CIR-18-0481

[207] Zheng X, Qian Y, Fu B, Jiao D, Jiang Y, Chen P, et al. Mitochondrial fragmentation limits NK cell-based tumor immunosurveillance. Nat Immunol. 2019;20:1656–67. 31636463 10.1038/s41590-019-0511-1

[208] Raskovalova T, Huang X, Sitkovsky M, Zacharia LC, Jackson EK, Gorelik E. Gs protein-coupled adenosine receptor signaling and lytic function of activated NK cells. J Immunol. 2005;175:4383–91. 16177079 10.4049/jimmunol.175.7.4383

[209] Ni J, Wang X, Stojanovic A, Zhang Q, Wincher M, Bühler L, et al. Single-cell RNA sequencing of tumor-infiltrating NK cells reveals that inhibition of transcription factor HIF-1α unleashes NK cell activity. Immunity. 2020;52:1075-1087.e8. 32445619 10.1016/j.immuni.2020.05.001

[210] Salzberger W, Martrus G, Bachmann K, Goebels H, Heß L, Koch M, et al. Tissue-resident NK cells differ in their expression profile of the nutrient transporters Glut1, CD98 and CD71. PLoS ONE. 2018;13: e0201170. 30028872 10.1371/journal.pone.0201170 PMC6054388

[211] Assmann N, O’Brien KL, Donnelly RP, Dyck L, Zaiatz-Bittencourt V, Loftus RM, et al. Srebp-controlled glucose metabolism is essential for NK cell functional responses. Nat Immunol. 2017;18:1197–206. 28920951 10.1038/ni.3838

[212] Terrén I, Orrantia A, Vitallé J, Zenarruzabeitia O, Borrego F. NK cell metabolism and tumor microenvironment. Front Immunol. 2019;10:2278. 31616440 10.3389/fimmu.2019.02278 PMC6769035

[213] Tiwary S, Berzofsky JA, Terabe M. Altered lipid tumor environment and its potential effects on NKT cell function in tumor immunity. Front Immunol. 2019;10:2187. 31620124 10.3389/fimmu.2019.02187 PMC6759687

[214] Chang C-H, Qiu J, O’Sullivan D, Buck MD, Noguchi T, Curtis JD, et al. Metabolic competition in the tumor microenvironment is a driver of cancer progression. Cell. 2015;162:1229–41. 26321679 10.1016/j.cell.2015.08.016 PMC4864363

[215] Cong J, Wang X, Zheng X, Wang D, Fu B, Sun R, et al. Dysfunction of natural killer cells by FBP1-induced inhibition of glycolysis during lung cancer progression. Cell Metab. 2018;28:243-255.e5. 30033198 10.1016/j.cmet.2018.06.021

[216] Wu Q, Ishikawa T, Sirianni R, Tang H, McDonald JG, Yuhanna IS, et al. 27-hydroxycholesterol promotes cell-autonomous ER-positive breast cancer growth. Cell Rep. 2013;5:637–45. 24210818 10.1016/j.celrep.2013.10.006 PMC3950897

[217] Rossin D, Dias IHK, Solej M, Milic I, Pitt AR, Iaia N, et al. Increased production of 27-hydroxycholesterol in human colorectal cancer advanced stage: possible contribution to cancer cell survival and infiltration. Free Radic Biol Med. 2019;136:35–44. 30910555 10.1016/j.freeradbiomed.2019.03.020

[218] Wang F, Meng M, Mo B, Yang Y, Ji Y, Huang P, et al. Crosstalks between mTORC1 and mTORC2 variagate cytokine signaling to control NK maturation and effector function. Nat Commun. 2018;9:4874. 30451838 10.1038/s41467-018-07277-9 PMC6242843

[219] Viel S, Marçais A, Guimaraes FS-F, Loftus R, Rabilloud J, Grau M, et al. TGF-β inhibits the activation and functions of NK cells by repressing the mTOR pathway. Sci Signal. 2016;9:ra19. 26884601 10.1126/scisignal.aad1884

[220] Claps G, Faouzi S, Quidville V, Chehade F, Shen S, Vagner S, et al. The multiple roles of LDH in cancer. Nat Rev Clin Oncol. 2022;19:749–62. 36207413 10.1038/s41571-022-00686-2

[221] Brand A, Singer K, Koehl GE, Kolitzus M, Schoenhammer G, Thiel A, et al. LDHA-associated lactic acid production blunts tumor immunosurveillance by T and NK cells. Cell Metab. 2016;24:657–71. 27641098 10.1016/j.cmet.2016.08.011

[222] Ge W, Meng L, Cao S, Hou C, Zhu X, Huang D, et al. The SIX1/LDHA axis promotes lactate accumulation and leads to NK cell dysfunction in pancreatic cancer. J Immunol Res. 2023;2023:6891636. 36937004 10.1155/2023/6891636 PMC10022590

[223] Reddy AT, Lakshmi SP, Reddy RC. PPAR *γ* as a novel therapeutic target in lung cancer. PPAR Res. 2016;2016:1–7. 10.1155/2016/8972570 PMC5028876 27698657

[224] Jiao D, Sun R, Ren X, Wang Y, Tian P, Wang Y, et al. Lipid accumulation-mediated histone hypoacetylation drives persistent NK cell dysfunction in anti-tumor immunity. Cell Rep. 2023;42:113211. 37792534 10.1016/j.celrep.2023.113211

[225] Lamas B, Vergnaud-Gauduchon J, Goncalves-Mendes N, Perche O, Rossary A, Vasson M-P, et al. Altered functions of natural killer cells in response to L-arginine availability. Cell Immunol. 2012;280:182–90. 23399839 10.1016/j.cellimm.2012.11.018

[226] Westhaver LP, Nersesian S, Nelson A, MacLean LK, Carter EB, Rowter D, et al. Mitochondrial damage-associated molecular patterns trigger arginase-dependent lymphocyte immunoregulation. Cell Rep. 2022;39:110847. 35613582 10.1016/j.celrep.2022.110847

[227] Aponte-López A, Fuentes-Pananá EM, Cortes-Muñoz D, Muñoz-Cruz S. Mast cell, the neglected member of the tumor microenvironment: role in breast cancer. J Immunol Res. 2018;2018:2584243. 29651440 10.1155/2018/2584243 PMC5832101

[228] Liu J, Zhang Y, Zhao J, Yang Z, Li D, Katirai F, et al. Mast cell: insight into remodeling a tumor microenvironment. Cancer Metastasis Rev. 2011;30:177–84. 21267769 10.1007/s10555-011-9276-1

[229] Mendoza RP, Fudge DH, Brown JM. Cellular energetics of mast cell development and activation. Cells. 2021;10:524. 33801300 10.3390/cells10030524 PMC7999080

[230] Marshall JS. Mast-cell responses to pathogens. Nat Rev Immunol. 2004;4:787–99. 15459670 10.1038/nri1460

[231] Zhou Z, Zheng J, Lu Y, Mai Z, Lin Y, Lin P, et al. Optimizing CD8+ T cell-based immunotherapy via metabolic interventions: a comprehensive review of intrinsic and extrinsic modulators. Exp Hematol Oncol. 2024;13:103. 39438986 10.1186/s40164-024-00575-7 PMC11495118

[232] Van der Vreken A, Vanderkerken K, De Bruyne E, De Veirman K, Breckpot K, Menu E. Fueling CARs: metabolic strategies to enhance CAR T-cell therapy. Exp Hematol Oncol. 2024;13:66. 38987856 10.1186/s40164-024-00535-1 PMC11238373

[233] Almeida L, Dhillon-LaBrooy A, Carriche G, Berod L, Sparwasser T. CD4+ T-cell differentiation and function: unifying glycolysis, fatty acid oxidation, polyamines NAD mitochondria. J Allergy Clin Immunol. 2021;148:16–32. 33966898 10.1016/j.jaci.2021.03.033

[234] Sawant DV, Yano H, Chikina M, Zhang Q, Liao M, Liu C, et al. Adaptive plasticity of IL-10+ and IL-35+ Treg cells cooperatively promotes tumor T cell exhaustion. Nat Immunol. 2019;20:724–35. 30936494 10.1038/s41590-019-0346-9 PMC6531353

[235] Liao M, Yao D, Wu L, Luo C, Wang Z, Zhang J, et al. Targeting the Warburg effect: a revisited perspective from molecular mechanisms to traditional and innovative therapeutic strategies in cancer. Acta Pharm Sin B. 2024;14:953–1008. 38487001 10.1016/j.apsb.2023.12.003 PMC10935242

[236] Cao J, Liao S, Zeng F, Liao Q, Luo G, Zhou Y. Effects of altered glycolysis levels on CD8+ T cell activation and function. Cell Death Dis. 2023;14:407. 37422501 10.1038/s41419-023-05937-3 PMC10329707

[237] Chang C-H, Curtis JD, Maggi LB, Faubert B, Villarino AV, O’Sullivan D, et al. Posttranscriptional control of T cell effector function by aerobic glycolysis. Cell. 2013;153:1239–51. 23746840 10.1016/j.cell.2013.05.016 PMC3804311

[238] Sena LA, Li S, Jairaman A, Prakriya M, Ezponda T, Hildeman DA, et al. Mitochondria are required for antigen-specific T cell activation through reactive oxygen species signaling. Immunity. 2013;38:225–36. 23415911 10.1016/j.immuni.2012.10.020 PMC3582741

[239] Angelin A, Gil-de-Gómez L, Dahiya S, Jiao J, Guo L, Levine MH, et al. Foxp3 reprograms T cell metabolism to function in low-glucose high-lactate environments. Cell Metab. 2017;25:1282-1293.e7. 28416194 10.1016/j.cmet.2016.12.018 PMC5462872

[240] Yan Y, Huang L, Liu Y, Yi M, Chu Q, Jiao D, et al. Metabolic profiles of regulatory T cells and their adaptations to the tumor microenvironment: implications for antitumor immunity. J Hematol OncolJ Hematol Oncol. 2022;15:104. 35948909 10.1186/s13045-022-01322-3 PMC9364625

[241] Wenes M, Jaccard A, Wyss T, Maldonado-Pérez N, Teoh ST, Lepez A, et al. The mitochondrial pyruvate carrier regulates memory T cell differentiation and antitumor function. Cell Metab. 2022;34:731-746.e9. 35452600 10.1016/j.cmet.2022.03.013 PMC9116152

[242] Gnanaprakasam JNR, Sherman JW, Wang R. MYC and HIF in shaping immune response and immune metabolism. Cytokine Growth Factor Rev. 2017;35:63–70. 28363691 10.1016/j.cytogfr.2017.03.004

[243] Wang Y, Bi Y, Chen X, Li C, Li Y, Zhang Z, et al. Histone deacetylase SIRT1 negatively regulates the differentiation of interleukin-9-producing CD4 + T cells. Immunity. 2016;44:1337–49. 27317260 10.1016/j.immuni.2016.05.009

[244] Finlay DK, Rosenzweig E, Sinclair LV, Feijoo-Carnero C, Hukelmann JL, Rolf J, et al. PDK1 regulation of mTOR and hypoxia-inducible factor 1 integrate metabolism and migration of CD8+ T cells. J Exp Med. 2012;209:2441–53. 23183047 10.1084/jem.20112607 PMC3526360

[245] Zeng H, Cohen S, Guy C, Shrestha S, Neale G, Brown SA, et al. mTORC1 and mTORC2 kinase signaling and glucose metabolism drive follicular helper T cell differentiation. Immunity. 2016;45:540–54. 27637146 10.1016/j.immuni.2016.08.017 PMC5050556

[246] Chen Y, Xu Z, Sun H, Ouyang X, Han Y, Yu H, et al. Regulation of CD8+ T memory and exhaustion by the mTOR signals. Cell Mol Immunol. 2023;20:1023–39. 37582972 10.1038/s41423-023-01064-3 PMC10468538

[247] Böttcher M, Hofmann AD, Bruns H, Haibach M, Loschinski R, Saul D, et al. Mesenchymal stromal cells disrupt mTOR-signaling and aerobic glycolysis during T-cell activation. Stem Cells Dayt Ohio. 2016;34:516–21. 10.1002/stem.2234 26485560

[248] Macintyre AN, Gerriets VA, Nichols AG, Michalek RD, Rudolph MC, Deoliveira D, et al. The glucose transporter Glut1 is selectively essential for CD4 T cell activation and effector function. Cell Metab. 2014;20:61–72. 24930970 10.1016/j.cmet.2014.05.004 PMC4079750

[249] Sun R-X, Liu Y-F, Sun Y-S, Zhou M, Wang Y, Shi B-Z, et al. GPC3-targeted CAR-T cells expressing GLUT1 or AGK exhibit enhanced antitumor activity against hepatocellular carcinoma. Acta Pharmacol Sin. 2024;45:1937–50. 38750075 10.1038/s41401-024-01287-8 PMC11336244

[250] Shi Y, Kotchetkov IS, Dobrin A, Hanina SA, Rajasekhar VK, Healey JH, et al. GLUT1 overexpression enhances CAR T cell metabolic fitness and anti-tumor efficacy. Mol Ther J Am Soc Gene Ther. 2024;32:2393–405. 10.1016/j.ymthe.2024.05.006 PMC11286825 38720457

[251] Fu H, Vuononvirta J, Fanti S, Bonacina F, D’Amati A, Wang G, et al. The glucose transporter 2 regulates CD8+ T cell function via environment sensing. Nat Metab. 2023;5:1969–85. 37884694 10.1038/s42255-023-00913-9 PMC10663157

[252] Feng Q, Liu Z, Yu X, Huang T, Chen J, Wang J, et al. Lactate increases stemness of CD8 + T cells to augment anti-tumor immunity. Nat Commun. 2022;13:4981. 36068198 10.1038/s41467-022-32521-8 PMC9448806

[253] Silic-Benussi M, Sharova E, Ciccarese F, Cavallari I, Raimondi V, Urso L, et al. mTOR inhibition downregulates glucose-6-phosphate dehydrogenase and induces ROS-dependent death in T-cell acute lymphoblastic leukemia cells. Redox Biol. 2022;51:102268. 35248829 10.1016/j.redox.2022.102268 PMC8899410

[254] Wang R, Dillon CP, Shi LZ, Milasta S, Carter R, Finkelstein D, et al. The transcription factor Myc controls metabolic reprogramming upon T lymphocyte activation. Immunity. 2011;35:871–82. 22195744 10.1016/j.immuni.2011.09.021 PMC3248798

[255] Daneshmandi S, Cassel T, Higashi RM, Fan TW-M, Seth P. 6-Phosphogluconate dehydrogenase (6PGD), a key checkpoint in reprogramming of regulatory T cells metabolism and function. Elife. 2021;10: e67476. 34709178 10.7554/eLife.67476 PMC8553335

[256] Daneshmandi S, Cassel T, Lin P, Higashi RM, Wulf GM, Boussiotis VA, et al. Blockade of 6-phosphogluconate dehydrogenase generates CD8+ effector T cells with enhanced anti-tumor function. Cell Rep. 2021;34:108831. 33691103 10.1016/j.celrep.2021.108831 PMC8051863

[257] Ma R, Ji T, Zhang H, Dong W, Chen X, Xu P, et al. A Pck1-directed glycogen metabolic program regulates formation and maintenance of memory CD8+ T cells. Nat Cell Biol. 2018;20:21–7. 29230018 10.1038/s41556-017-0002-2

[258] Lam C, Low J-Y, Tran PT, Wang H. The hexosamine biosynthetic pathway and cancer: current knowledge and future therapeutic strategies. Cancer Lett. 2021;503:11–8. 33484754 10.1016/j.canlet.2021.01.010

[259] Swamy M, Pathak S, Grzes KM, Damerow S, Sinclair LV, van Aalten DMF, et al. Glucose and glutamine fuel protein O-GlcNAcylation to control T cell self-renewal and malignancy. Nat Immunol. 2016;17:712–20. 27111141 10.1038/ni.3439 PMC4900450

[260] Sun W, Liu R, Gao X, Lin Z, Tang H, Cui H, et al. Targeting serine-glycine-one-carbon metabolism as a vulnerability in cancers. Biomark Res. 2023;11:48. 37147729 10.1186/s40364-023-00487-4 PMC10161514

[261] Ma EH, Bantug G, Griss T, Condotta S, Johnson RM, Samborska B, et al. Serine is an essential metabolite for effector T cell expansion. Cell Metab. 2017;25:345–57. 28111214 10.1016/j.cmet.2016.12.011

[262] Matias MI, Yong CS, Foroushani A, Goldsmith C, Mongellaz C, Sezgin E, et al. Regulatory T cell differentiation is controlled by αKG-induced alterations in mitochondrial metabolism and lipid homeostasis. Cell Rep. 2021;37:109911. 34731632 10.1016/j.celrep.2021.109911 PMC10167917

[263] Xu T, Stewart KM, Wang X, Liu K, Xie M, Ryu JK, et al. Metabolic control of TH17 and induced Treg cell balance by an epigenetic mechanism. Nature. 2017;548:228–33. 28783731 10.1038/nature23475 PMC6701955

[264] Gudgeon N, Munford H, Bishop EL, Hill J, Fulton-Ward T, Bending D, et al. Succinate uptake by T cells suppresses their effector function via inhibition of mitochondrial glucose oxidation. Cell Rep. 2022;40:111193. 35977513 10.1016/j.celrep.2022.111193 PMC9638018

[265] Huang L, Li H, Zhang C, Chen Q, Liu Z, Zhang J, et al. Unlocking the potential of T-cell metabolism reprogramming: advancing single-cell approaches for precision immunotherapy in tumour immunity. Clin Transl Med. 2024;14: e1620. 38468489 10.1002/ctm2.1620 PMC10928360

[266] Ho P-C, Bihuniak JD, Macintyre AN, Staron M, Liu X, Amezquita R, et al. Phosphoenolpyruvate Is a metabolic checkpoint of anti-tumor T cell responses. Cell. 2015;162:1217–28. 26321681 10.1016/j.cell.2015.08.012 PMC4567953

[267] He J, Shangguan X, Zhou W, Cao Y, Zheng Q, Tu J, et al. Glucose limitation activates AMPK coupled SENP1-Sirt3 signalling in mitochondria for T cell memory development. Nat Commun. 2021;12:4371. 34272364 10.1038/s41467-021-24619-2 PMC8285428

[268] Wang T, Gnanaprakasam JNR, Chen X, Kang S, Xu X, Sun H, et al. Inosine is an alternative carbon source for CD8+-T-cell function under glucose restriction. Nat Metab. 2020;2:635–47. 32694789 10.1038/s42255-020-0219-4 PMC7371628

[269] Liu Y, Liang G, Xu H, Dong W, Dong Z, Qiu Z, et al. Tumors exploit FTO-mediated regulation of glycolytic metabolism to evade immune surveillance. Cell Metab. 2021;33:1221-1233.e11. 33910046 10.1016/j.cmet.2021.04.001

[270] Lei J, Yang Y, Lu Z, Pan H, Fang J, Jing B, et al. Taming metabolic competition via glycolysis inhibition for safe and potent tumor immunotherapy. Biochem Pharmacol. 2022;202:115153. 35750199 10.1016/j.bcp.2022.115153

[271] Vardhana SA, Hwee MA, Berisa M, Wells DK, Yost KE, King B, et al. Impaired mitochondrial oxidative phosphorylation limits the self-renewal of T cells exposed to persistent antigen. Nat Immunol. 2020;21:1022–33. 32661364 10.1038/s41590-020-0725-2 PMC7442749

[272] Hong HS, Mbah NE, Shan M, Loesel K, Lin L, Sajjakulnukit P, et al. OXPHOS promotes apoptotic resistance and cellular persistence in TH17 cells in the periphery and tumor microenvironment. Sci Immunol. 2022;7:8182. 10.1126/sciimmunol.abm8182 PMC9853437 36399539

[273] Jackson CM, Pant A, Dinalankara W, Choi J, Jain A, Nitta R, et al. The cytokine Meteorin-like inhibits anti-tumor CD8+ T cell responses by disrupting mitochondrial function. Immunity. 2024;57:1864-1877.e9. 39111315 10.1016/j.immuni.2024.07.003 PMC11324406

[274] Buck MD, O’Sullivan D, Klein Geltink RI, Curtis JD, Chang C-H, Sanin DE, et al. Mitochondrial dynamics controls T cell fate through metabolic programming. Cell. 2016;166:63–76. 27293185 10.1016/j.cell.2016.05.035 PMC4974356

[275] Glorieux C, Liu S, Trachootham D, Huang P. Targeting ROS in cancer: rationale and strategies. Nat Rev Drug Discov. 2024;23:583–606. 38982305 10.1038/s41573-024-00979-4

[276] Siska PJ, Beckermann KE, Mason FM, Andrejeva G, Greenplate AR, Sendor AB, et al. Mitochondrial dysregulation and glycolytic insufficiency functionally impair CD8 T cells infiltrating human renal cell carcinoma. JCI Insight. 2017;2: e93411. 28614802 10.1172/jci.insight.93411 PMC5470888

[277] Scharping NE, Menk AV, Moreci RS, Whetstone RD, Dadey RE, Watkins SC, et al. The tumor microenvironment represses T cell mitochondrial biogenesis to drive intratumoral T cell metabolic insufficiency and dysfunction. Immunity. 2016;45:374–88. 27496732 10.1016/j.immuni.2016.07.009 PMC5207350

[278] Field CS, Baixauli F, Kyle RL, Puleston DJ, Cameron AM, Sanin DE, et al. Mitochondrial integrity regulated by lipid metabolism is a cell-intrinsic checkpoint for Treg suppressive function. Cell Metab. 2020;31:422-437.e5. 31883840 10.1016/j.cmet.2019.11.021 PMC7001036

[279] Berod L, Friedrich C, Nandan A, Freitag J, Hagemann S, Harmrolfs K, et al. De novo fatty acid synthesis controls the fate between regulatory T and T helper 17 cells. Nat Med. 2014;20:1327–33. 25282359 10.1038/nm.3704

[280] Trompette A, Gollwitzer ES, Pattaroni C, Lopez-Mejia IC, Riva E, Pernot J, et al. Dietary fiber confers protection against flu by shaping Ly6c− patrolling monocyte hematopoiesis and CD8+ T cell metabolism. Immunity. 2018;48:992-1005.e8. 29768180 10.1016/j.immuni.2018.04.022

[281] Bachem A, Makhlouf C, Binger KJ, de Souza DP, Tull D, Hochheiser K, et al. Microbiota-derived short-chain fatty acids promote the memory potential of antigen-activated CD8+ T cells. Immunity. 2019;51:285-297.e5. 31272808 10.1016/j.immuni.2019.06.002

[282] Haghikia A, Jörg S, Duscha A, Berg J, Manzel A, Waschbisch A, et al. Dietary fatty acids directly impact central nervous system autoimmunity via the small intestine. Immunity. 2015;43:817–29. 26488817 10.1016/j.immuni.2015.09.007

[283] Lochner M, Berod L, Sparwasser T. Fatty acid metabolism in the regulation of T cell function. Trends Immunol. 2015;36:81–91. 25592731 10.1016/j.it.2014.12.005

[284] Endo Y, Onodera A, Obata-Ninomiya K, Koyama-Nasu R, Asou HK, Ito T, et al. ACC1 determines memory potential of individual CD4+ T cells by regulating de novo fatty acid biosynthesis. Nat Metab. 2019;1:261–75. 32694782 10.1038/s42255-018-0025-4

[285] Lee J, Walsh MC, Hoehn KL, James DE, Wherry EJ, Choi Y. Regulator of fatty acid metabolism, acetyl coenzyme a carboxylase 1. Controls T Cell Immunity J Immunol. 2014;192:3190–9. 24567531 10.4049/jimmunol.1302985 PMC3965631

[286] Hunt EG, Hurst KE, Riesenberg BP, Kennedy AS, Gandy EJ, Andrews AM, et al. Acetyl-CoA carboxylase obstructs CD8+ T cell lipid utilization in the tumor microenvironment. Cell Metab. 2024;36:969-983.e10. 38490211 10.1016/j.cmet.2024.02.009 PMC12010431

[287] Pearce EL, Walsh MC, Cejas PJ, Harms GM, Shen H, Wang L-S, et al. Enhancing CD8 T-cell memory by modulating fatty acid metabolism. Nature. 2009;460:103–7. 19494812 10.1038/nature08097 PMC2803086

[288] Rolf J, Zarrouk M, Finlay DK, Foretz M, Viollet B, Cantrell DA. AMPK α1: a glucose sensor that controls CD 8 T -cell memory. Eur J Immunol. 2013;43:889–96. 23310952 10.1002/eji.201243008 PMC3734624

[289] Ecker C, Guo L, Voicu S, Gil-de-Gómez L, Medvec A, Cortina L, et al. Differential reliance on lipid metabolism as a salvage pathway underlies functional differences of T cell subsets in poor nutrient environments. Cell Rep. 2018;23:741–55. 29669281 10.1016/j.celrep.2018.03.084 PMC5929999

[290] Kidani Y, Elsaesser H, Hock MB, Vergnes L, Williams KJ, Argus JP, et al. Sterol regulatory element-binding proteins are essential for the metabolic programming of effector T cells and adaptive immunity. Nat Immunol. 2013;14:489–99. 23563690 10.1038/ni.2570 PMC3652626

[291] Bensinger SJ, Bradley MN, Joseph SB, Zelcer N, Janssen EM, Hausner MA, et al. LXR signaling couples sterol metabolism to proliferation in the acquired immune response. Cell. 2008;134:97–111. 18614014 10.1016/j.cell.2008.04.052 PMC2626438

[292] Kennewick KT, Bensinger SJ. Decoding the crosstalk between mevalonate metabolism and T cell function. Immunol Rev. 2023;317:71–94. 36999733 10.1111/imr.13200 PMC12136499

[293] Chapman NM, Boothby MR, Chi H. Metabolic coordination of T cell quiescence and activation. Nat Rev Immunol. 2020;20:55–70. 31406325 10.1038/s41577-019-0203-y

[294] Cui C, Merritt R, Fu L, Pan Z. Targeting calcium signaling in cancer therapy. Acta Pharm Sin B. 2017;7:3–17. 28119804 10.1016/j.apsb.2016.11.001 PMC5237760

[295] Okada N, Sugiyama K, Kitamura H, Taketomi A. Inhibition of diacylglycerol kinase alpha to augment antitumor effector T cells in tumor-bearing host. J Clin Oncol. 2019;37:293–293.

[296] Summers SA, Chaurasia B, Holland WL. Metabolic messengers: ceramides. Nat Metab. 2019;1:1051–8. 32694860 10.1038/s42255-019-0134-8 PMC7549391

[297] Li G, Liu D, Kimchi ET, Kaifi JT, Qi X, Manjunath Y, et al. Nanoliposome C6-ceramide increases the anti-tumor immune response and slows growth of liver tumors in mice. Gastroenterology. 2018;154:1024-1036.e9. 29408569 10.1053/j.gastro.2017.10.050 PMC5908238

[298] Ohtani N, Hara E. Gut-liver axis-mediated mechanism of liver cancer: a special focus on the role of gut microbiota. Cancer Sci. 2021;112:4433–43. 34533882 10.1111/cas.15142 PMC8586687

[299] Smith PM, Howitt MR, Panikov N, Michaud M, Gallini CA, Bohlooly-Y M, et al. The microbial metabolites, short-chain fatty acids, regulate colonic T _reg_ cell homeostasis. Science. 2013;341:569–73. 23828891 10.1126/science.1241165 PMC3807819

[300] Manzo T, Prentice BM, Anderson KG, Raman A, Schalck A, Codreanu GS, et al. Accumulation of long-chain fatty acids in the tumor microenvironment drives dysfunction in intrapancreatic CD8+ T cells. J Exp Med. 2020;217: e20191920. 32491160 10.1084/jem.20191920 PMC7398173

[301] Wang H, Franco F, Tsui Y-C, Xie X, Trefny MP, Zappasodi R, et al. CD36-mediated metabolic adaptation supports regulatory T cell survival and function in tumors. Nat Immunol. 2020;21:298–308. 32066953 10.1038/s41590-019-0589-5 PMC7043937

[302] Ma X, Xiao L, Liu L, Ye L, Su P, Bi E, et al. CD36-mediated ferroptosis dampens intratumoral CD8+ T cell effector function and impairs their antitumor ability. Cell Metab. 2021;33:1001-1012.e5. 33691090 10.1016/j.cmet.2021.02.015 PMC8102368

[303] Xu S, Chaudhary O, Rodríguez-Morales P, Sun X, Chen D, Zappasodi R, et al. Uptake of oxidized lipids by the scavenger receptor CD36 promotes lipid peroxidation and dysfunction in CD8+ T cells in tumors. Immunity. 2021;54:1561-1577.e7. 34102100 10.1016/j.immuni.2021.05.003 PMC9273026

[304] Guo H-Z, Feng R-X, Zhang Y-J, Yu Y-H, Lu W, Liu J-J, et al. A CD36-dependent non-canonical lipid metabolism program promotes immune escape and resistance to hypomethylating agent therapy in AML. Cell Rep Med. 2024;5:101592. 38843841 10.1016/j.xcrm.2024.101592 PMC11228649

[305] Christofides A, Konstantinidou E, Jani C, Boussiotis VA. The role of peroxisome proliferator-activated receptors (PPAR) in immune responses. Metabolism. 2021;114:154338. 32791172 10.1016/j.metabol.2020.154338 PMC7736084

[306] Klotz L, Burgdorf S, Dani I, Saijo K, Flossdorf J, Hucke S, et al. The nuclear receptor PPAR gamma selectively inhibits Th17 differentiation in a T cell-intrinsic fashion and suppresses CNS autoimmunity. J Exp Med. 2009;206:2079–89. 19737866 10.1084/jem.20082771 PMC2757877

[307] Cipolletta D, Feuerer M, Li A, Kamei N, Lee J, Shoelson SE, et al. PPAR-γ is a major driver of the accumulation and phenotype of adipose tissue Treg cells. Nature. 2012;486:549–53. 22722857 10.1038/nature11132 PMC3387339

[308] Tian M, Hao F, Jin X, Sun X, Jiang Y, Wang Y, et al. ACLY ubiquitination by CUL3-KLHL25 induces the reprogramming of fatty acid metabolism to facilitate iTreg differentiation. Elife. 2021;10: e62394. 34491895 10.7554/eLife.62394 PMC8423445

[309] Michalek RD, Gerriets VA, Jacobs SR, Macintyre AN, MacIver NJ, Mason EF, et al. Cutting edge: distinct glycolytic and lipid oxidative metabolic programs are essential for effector and regulatory CD4+ T cell subsets. J Immunol Baltim Md. 1950;2011(186):3299–303. 10.4049/jimmunol.1003613 PMC3198034 21317389

[310] Hao F, Tian M, Zhang X, Jin X, Jiang Y, Sun X, et al. Butyrate enhances CPT1A activity to promote fatty acid oxidation and iTreg differentiation. Proc Natl Acad Sci. 2021;118: e2014681118. 34035164 10.1073/pnas.2014681118 PMC8179238

[311] Howie D, Ten Bokum A, Cobbold SP, Yu Z, Kessler BM, Waldmann H. A novel role for triglyceride metabolism in Foxp3 expression. Front Immunol. 2019;10:1860. 31456800 10.3389/fimmu.2019.01860 PMC6701200

[312] Take Y, Koizumi S, Nagahisa A. Prostaglandin E receptor 4 antagonist in cancer immunotherapy: mechanisms of action. Front Immunol. 2020;11:324. 32210957 10.3389/fimmu.2020.00324 PMC7076081

[313] Lacher SB, Dörr J, De Almeida GP, Hönninger J, Bayerl F, Hirschberger A, et al. PGE2 limits effector expansion of tumour-infiltrating stem-like CD8+ T cells. Nature. 2024;629:417–25. 38658748 10.1038/s41586-024-07254-x PMC11078747

[314] Yang W, Bai Y, Xiong Y, Zhang J, Chen S, Zheng X, et al. Potentiating the antitumour response of CD8(+) T cells by modulating cholesterol metabolism. Nature. 2016;531:651–5. 26982734 10.1038/nature17412 PMC4851431

[315] Yan C, Zheng L, Jiang S, Yang H, Guo J, Jiang L-Y, et al. Exhaustion-associated cholesterol deficiency dampens the cytotoxic arm of antitumor immunity. Cancer Cell. 2023;41:1276-1293.e11. 37244259 10.1016/j.ccell.2023.04.016

[316] Shuwen H, Yinhang W, Jing Z, Qiang Y, Yizhen J, Quan Q, et al. Cholesterol induction in CD8+ T cell exhaustion in colorectal cancer via the regulation of endoplasmic reticulum-mitochondria contact sites. Cancer Immunol Immunother. 2023;72:4441–56. 37919522 10.1007/s00262-023-03555-8 PMC10991466

[317] Picarda E, Ren X, Zang X. Tumor cholesterol up T cells down. Cell Metab. 2019;30:12–3. 31269422 10.1016/j.cmet.2019.06.007

[318] Mossmann D, Park S, Hall MN. mTOR signalling and cellular metabolism are mutual determinants in cancer. Nat Rev Cancer. 2018;18:744–57. 30425336 10.1038/s41568-018-0074-8

[319] Ping Y, Shan J, Qin H, Li F, Qu J, Guo R, et al. PD-1 signaling limits expression of phospholipid phosphatase 1 and promotes intratumoral CD8+ T cell ferroptosis. Immunity. 2024;57:2122-2139.e9. 39208806 10.1016/j.immuni.2024.08.003

[320] Zarrin AA, Bao K, Lupardus P, Vucic D. Kinase inhibition in autoimmunity and inflammation. Nat Rev Drug Discov. 2021;20:39–63. 33077936 10.1038/s41573-020-0082-8 PMC7569567

[321] Turner JA, Fredrickson MA, D’Antonio M, Katsnelson E, MacBeth M, Van Gulick R, et al. Lysophosphatidic acid modulates CD8 T cell immunosurveillance and metabolism to impair anti-tumor immunity. Nat Commun. 2023;14:3214. 37270644 10.1038/s41467-023-38933-4 PMC10239450

[322] Ma S, Sandhoff R, Luo X, Shang F, Shi Q, Li Z, et al. Serine enrichment in tumors promotes regulatory T cell accumulation through sphinganine-mediated regulation of c-Fos. Sci Immunol. 2024;9:eadg8817. 38640251 10.1126/sciimmunol.adg8817

[323] Liu X, Hartman CL, Li L, Albert CJ, Si F, Gao A, et al. Reprogramming lipid metabolism prevents effector T cell senescence and enhances tumor immunotherapy. Sci Transl Med. 2021;13:eaaz6314. 33790024 10.1126/scitranslmed.aaz6314 PMC12040281

[324] Hu X, Guo F. Amino acid sensing in metabolic homeostasis and health. Endocr Rev. 2021;42:56–76. 33053153 10.1210/endrev/bnaa026

[325] Nakaya M, Xiao Y, Zhou X, Chang J-H, Chang M, Cheng X, et al. Inflammatory T cell responses rely on amino acid transporter ASCT2 facilitation of glutamine uptake and mTORC1 kinase activation. Immunity. 2014;40:692–705. 24792914 10.1016/j.immuni.2014.04.007 PMC4074507

[326] Huang H, Zhou P, Wei J, Long L, Shi H, Dhungana Y, et al. In vivo CRISPR screening reveals nutrient signaling processes underpinning CD8+ T cell fate decisions. Cell. 2021;184:1245-1261.e21. 33636132 10.1016/j.cell.2021.02.021 PMC8101447

[327] Ijare OB, Hambarde S, Brasil Da Costa FH, Lopez S, Sharpe MA, Helekar SA, et al. Glutamine anaplerosis is required for amino acid biosynthesis in human meningiomas. Neuro-Oncol. 2022;24:556–68. 34515312 10.1093/neuonc/noab219 PMC8972231

[328] Li X, Peng X, Li Y, Wei S, He G, Liu J, et al. Glutamine addiction in tumor cell: oncogene regulation and clinical treatment. Cell Commun Signal. 2024;22:12. 38172980 10.1186/s12964-023-01449-x PMC10763057

[329] Leone RD, Zhao L, Englert JM, Sun I-M, Oh M-H, Sun I-H, et al. Glutamine blockade induces divergent metabolic programs to overcome tumor immune evasion. Science. 2019;366:1013–21. 31699883 10.1126/science.aav2588 PMC7023461

[330] Peng H, Wang Y, Luo W. Multifaceted role of branched-chain amino acid metabolism in cancer. Oncogene. 2020;39:6747–56. 32978521 10.1038/s41388-020-01480-z PMC7606751

[331] Fotiadis D, Kanai Y, Palacín M. The SLC3 and SLC7 families of amino acid transporters. Mol Aspects Med. 2013;34:139–58. 23506863 10.1016/j.mam.2012.10.007

[332] Zhang Y, Hu H, Liu W, Yan S-M, Li Y, Tan L, et al. Amino acids and RagD potentiate mTORC1 activation in CD8 ^+^ T cells to confer antitumor immunity. J Immunother Cancer. 2021;9: e002137. 33883257 10.1136/jitc-2020-002137 PMC8061841

[333] Kang YJ, Song W, Lee SJ, Choi SA, Chae S, Yoon BR, et al. Inhibition of BCAT1-mediated cytosolic leucine metabolism regulates Th17 responses via the mTORC1-HIF1α pathway. Exp Mol Med. 2024;56:1776–90. 39085353 10.1038/s12276-024-01286-z PMC11372109

[334] Huang X, Sun T, Wang J, Hong X, Chen H, Yan T, et al. Metformin reprograms tryptophan metabolism to stimulate CD8+ T-cell function in colorectal cancer. Cancer Res. 2023;83:2358–71. 37195082 10.1158/0008-5472.CAN-22-3042

[335] Munn DH, Sharma MD, Baban B, Harding HP, Zhang Y, Ron D, et al. GCN2 kinase in T cells mediates proliferative arrest and anergy induction in response to indoleamine 2,3-dioxygenase. Immunity. 2005;22:633–42. 15894280 10.1016/j.immuni.2005.03.013

[336] Qin R, Zhao C, Wang C-J, Xu W, Zhao J-Y, Lin Y, et al. Tryptophan potentiates CD8 ^+^ T cells against cancer cells by TRIP12 tryptophanylation and surface PD-1 downregulation. J Immunother Cancer. 2021;9: e002840. 34326168 10.1136/jitc-2021-002840 PMC8323461

[337] Bessede A, Peyraud F, Le Moulec S, Cousin S, Cabart M, Chomy F, et al. Upregulation of indoleamine 2,3-dioxygenase 1 in tumor cells and tertiary lymphoid structures is a hallmark of inflamed non-small cell lung cancer. Clin Cancer Res. 2023;29:4883–93. 37756581 10.1158/1078-0432.CCR-23-1928 PMC10690088

[338] Liu Y, Chen S, Wan X, Wang R, Luo H, Chang C, et al. Tryptophan 2,3-dioxygenase-positive matrix fibroblasts fuel breast cancer lung metastasis via kynurenine-mediated ferroptosis resistance of metastatic cells and T cell dysfunction. Cancer Commun Lond Engl. 2024;44:1261–86. 10.1002/cac2.12608 PMC11570772 39221971

[339] Huang J, Liu D, Wang Y, Liu L, Li J, Yuan J, et al. Ginseng polysaccharides alter the gut microbiota and kynurenine/tryptophan ratio, potentiating the antitumour effect of antiprogrammed cell death 1/programmed cell death ligand 1 (anti-PD-1/PD-L1) immunotherapy. Gut. 2022;71:734–45. 34006584 10.1136/gutjnl-2020-321031 PMC8921579

[340] Campesato LF, Budhu S, Tchaicha J, Weng C-H, Gigoux M, Cohen IJ, et al. Blockade of the AHR restricts a Treg-macrophage suppressive axis induced by L-Kynurenine. Nat Commun. 2020;11:4011. 32782249 10.1038/s41467-020-17750-z PMC7419300

[341] L-Arginine Regulates T-cell Metabolism to Promote Antitumor Activity. Cancer Discov. 2016; 6:1302–1302

[342] Jungnickel KEJ, Parker JL, Newstead S. Structural basis for amino acid transport by the CAT family of SLC7 transporters. Nat Commun. 2018;9:550. 29416041 10.1038/s41467-018-03066-6 PMC5803215

[343] Meza-Perez S, Liu M, Silva-Sanchez A, Morrow CD, Eipers PG, Lefkowitz EJ, et al. Proteobacteria impair anti-tumor immunity in the omentum by consuming arginine. Cell Host Microbe. 2024;32:1177-1191.e7. 38942027 10.1016/j.chom.2024.06.003 PMC11245731

[344] Zou Z, Cheng Q, Zhou J, Guo C, Hadjinicolaou AV, Salio M, et al. ATF4-SLC7A11-GSH axis mediates the acquisition of immunosuppressive properties by activated CD4+ T cells in low arginine condition. Cell Rep. 2024;43:113995. 38527061 10.1016/j.celrep.2024.113995

[345] McGovern N, Shin A, Low G, Low D, Duan K, Yao LJ, et al. Human fetal dendritic cells promote prenatal T-cell immune suppression through arginase-2. Nature. 2017;546:662–6. 28614294 10.1038/nature22795 PMC6588541

[346] Yeh C-L, Tanuseputero SA, Wu J-M, Tseng Y-R, Yang P-J, Lee P-C, et al. Intravenous arginine administration benefits CD4+ T-cell homeostasis and attenuates liver inflammation in mice with polymicrobial sepsis. Nutrients. 2020;12:1047. 32290120 10.3390/nu12041047 PMC7231035

[347] Bedford MT, Richard S. Arginine methylation an emerging regulator of protein function. Mol Cell. 2005;18:263–72. 15866169 10.1016/j.molcel.2005.04.003

[348] Jiang Y, Yuan Y, Chen M, Li S, Bai J, Zhang Y, et al. PRMT5 disruption drives antitumor immunity in cervical cancer by reprogramming T cell-mediated response and regulating PD-L1 expression. Theranostics. 2021;11:9162–76. 34522232 10.7150/thno.59605 PMC8419032

[349] Sanderson SM, Gao X, Dai Z, Locasale JW. Methionine metabolism in health and cancer: a nexus of diet and precision medicine. Nat Rev Cancer. 2019;19:625–37. 31515518 10.1038/s41568-019-0187-8

[350] Pandit M, Kil Y-S, Ahn J-H, Pokhrel RH, Gu Y, Mishra S, et al. Methionine consumption by cancer cells drives a progressive upregulation of PD-1 expression in CD4 T cells. Nat Commun. 2023;14:2593. 37147330 10.1038/s41467-023-38316-9 PMC10162977

[351] Marchingo JM, Sinclair LV, Howden AJ, Cantrell DA. Quantitative analysis of how Myc controls T cell proteomes and metabolic pathways during T cell activation. Elife. 2020;9: e53725. 32022686 10.7554/eLife.53725 PMC7056270

[352] Bian Y, Li W, Kremer DM, Sajjakulnukit P, Li S, Crespo J, et al. Cancer SLC43A2 alters T cell methionine metabolism and histone methylation. Nature. 2020;585:277–82. 32879489 10.1038/s41586-020-2682-1 PMC7486248

[353] Gjuka D, Adib E, Garrison K, Chen J, Zhang Y, Li W, et al. Enzyme-mediated depletion of methylthioadenosine restores T cell function in MTAP-deficient tumors and reverses immunotherapy resistance. Cancer Cell. 2023;41:1774-1787.e9. 37774699 10.1016/j.ccell.2023.09.005 PMC10591910

[354] Yang M, Vousden KH. Serine and one-carbon metabolism in cancer. Nat Rev Cancer. 2016;16:650–62. 27634448 10.1038/nrc.2016.81

[355] Kurniawan H, Franchina DG, Guerra L, Bonetti L, Baguet LS, Grusdat M, et al. Glutathione restricts serine metabolism to preserve regulatory T cell function. Cell Metab. 2020;31:920-936.e7. 32213345 10.1016/j.cmet.2020.03.004 PMC7265172

[356] Hu X, Zhang J, Wang J, Fu J, Li T, Zheng X, et al. Landscape of B cell immunity and related immune evasion in human cancers. Nat Genet. 2019;51:560–7. 30742113 10.1038/s41588-018-0339-x PMC6773274

[357] Fridman WH, Meylan M, Petitprez F, Sun C-M, Italiano A, Sautès-Fridman C. B cells and tertiary lymphoid structures as determinants of tumour immune contexture and clinical outcome. Nat Rev Clin Oncol. 2022;19:441–57. 35365796 10.1038/s41571-022-00619-z

[358] Laumont CM, Nelson BH. B cells in the tumor microenvironment: multi-faceted organizers, regulators, and effectors of anti-tumor immunity. Cancer Cell. 2023;41:466–89. 36917951 10.1016/j.ccell.2023.02.017

[359] Michaud D, Steward CR, Mirlekar B, Pylayeva-Gupta Y. Regulatory B cells in cancer. Immunol Rev. 2021;299:74–92. 33368346 10.1111/imr.12939 PMC7965344

[360] Jellusova J, Cato MH, Apgar JR, Ramezani-Rad P, Leung CR, Chen C, et al. Gsk3 is a metabolic checkpoint regulator in B cells. Nat Immunol. 2017;18:303–12. 28114292 10.1038/ni.3664 PMC5310963

[361] Waters LR, Ahsan FM, Wolf DM, Shirihai O, Teitell MA. Initial B cell activation induces metabolic reprogramming and mitochondrial remodeling. iScience. 2018;5:99–109. 30240649 10.1016/j.isci.2018.07.005 PMC6123864

[362] Chen D, Wang Y, Manakkat Vijay GK, Fu S, Nash CW, Xu D, et al. Coupled analysis of transcriptome and BCR mutations reveals role of OXPHOS in affinity maturation. Nat Immunol. 2021;22:904–13. 34031613 10.1038/s41590-021-00936-y

[363] Imahashi N, Basar R, Huang Y, Wang F, Baran N, Banerjee PP, et al. Activated B cells suppress T-cell function through metabolic competition. J Immunother Cancer. 2022;10: e005644. 36543374 10.1136/jitc-2022-005644 PMC9772692

[364] Piper CJM, Rosser EC, Oleinika K, Nistala K, Krausgruber T, Rendeiro AF, et al. Aryl hydrocarbon receptor contributes to the transcriptional program of IL-10-producing regulatory B cells. Cell Rep. 2019;29:1878-1892.e7. 31722204 10.1016/j.celrep.2019.10.018 PMC6856759

[365] Kim M, Qie Y, Park J, Kim CH. Gut microbial metabolites fuel host antibody responses. Cell Host Microbe. 2016;20:202–14. 27476413 10.1016/j.chom.2016.07.001 PMC4982788

[366] Zhou X, Zhu X, Li C, Li Y, Ye Z, Shapiro VS, et al. Stearoyl-CoA desaturase-mediated monounsaturated fatty acid availability supports humoral immunity. Cell Rep. 2021;34:108601. 33406440 10.1016/j.celrep.2020.108601 PMC7839063

[367] Ma J, Wu Y, Ma L, Yang X, Zhang T, Song G, et al. A blueprint for tumor-infiltrating B cells across human cancers. Science. 2024;384:eadj4857. 38696569 10.1126/science.adj4857

[368] Wang Z, Lu Z, Lin S, Xia J, Zhong Z, Xie Z, et al. Leucine-tRNA-synthetase-2-expressing B cells contribute to colorectal cancer immunoevasion. Immunity. 2022;55:1067-1081.e8. 35659337 10.1016/j.immuni.2022.04.017

[369] Wang H, Franco F, Ho P-C. Metabolic regulation of Tregs in cancer: opportunities for immunotherapy. Trends Cancer. 2017;3:583–92. 28780935 10.1016/j.trecan.2017.06.005

[370] Tay C, Tanaka A, Sakaguchi S. Tumor-infiltrating regulatory T cells as targets of cancer immunotherapy. Cancer Cell. 2023;41:450–65. 36917950 10.1016/j.ccell.2023.02.014

[371] Wu M, Chen X, Lou J, Zhang S, Zhang X, Huang L, et al. Changes in regulatory T cells in patients with ovarian cancer undergoing surgery: Preliminary results. Int Immunopharmacol. 2017;47:244–50. 28437737 10.1016/j.intimp.2017.04.004

[372] Vacchelli E, Semeraro M, Adam J, Dartigues P, Zitvogel L, Kroemer G. Immunosurveillance in esophageal carcinoma: the decisive impact of regulatory T cells. OncoImmunology. 2016;5: e1064581. 27057430 10.1080/2162402X.2015.1064581 PMC4801440

[373] Wei T, Zhong W, Li Q. Role of heterogeneous regulatory T cells in the tumor microenvironment. Pharmacol Res. 2020;153:104659. 31982490 10.1016/j.phrs.2020.104659

[374] Wang Y, Li X-L, Mo Y-Z, Fan C-M, Tang L, Xiong F, et al. Effects of tumor metabolic microenvironment on regulatory T cells. Mol Cancer. 2018;17:168. 30477520 10.1186/s12943-018-0913-y PMC6260778

[375] Chiang Y, Lu L-F, Tsai C-L, Tsai Y-C, Wang C-C, Hsueh F-J, et al. C-C chemokine receptor 4 (CCR4)-positive regulatory T cells interact with tumor-associated macrophages to facilitate metastatic potential after radiation. Eur J Cancer. 2024;198:113521. 38171115 10.1016/j.ejca.2023.113521

[376] Haist M, Stege H, Grabbe S, Bros M. The functional crosstalk between myeloid-derived suppressor cells and regulatory T cells within the immunosuppressive tumor microenvironment. Cancers. 2021;13:210. 33430105 10.3390/cancers13020210 PMC7827203

[377] Fan H, Wu Y, Yu S, Li X, Wang A, Wang S, et al. Critical role of mTOR in regulating aerobic glycolysis in carcinogenesis (Review). Int J Oncol. 2020;58:9–19. 33367927 10.3892/ijo.2020.5152

[378] Rao D, Verburg F, Renner K, Peeper DS, Lacroix R, Blank CU. Metabolic profiles of regulatory T cells in the tumour microenvironment. Cancer Immunol Immunother. 2021;70:2417–27. 33576875 10.1007/s00262-021-02881-z PMC10991195

[379] Charbonnier L-M, Cui Y, Stephen-Victor E, Harb H, Lopez D, Bleesing JJ, et al. Functional reprogramming of regulatory T cells in the absence of Foxp3. Nat Immunol. 2019;20:1208–19. 31384057 10.1038/s41590-019-0442-x PMC6707855

[380] Alon R. A sweet solution: glycolysis-dependent Treg cell migration. Immunity. 2017;47:805–7. 29166580 10.1016/j.immuni.2017.11.006

[381] Hirsch T, Rothoeft T, Teig N, Bauer JW, Pellegrini G, De Rosa L, et al. Regeneration of the entire human epidermis using transgenic stem cells. Nature. 2017;551:327–32. 29144448 10.1038/nature24487 PMC6283270

[382] Kishore M, Cheung KCP, Fu H, Bonacina F, Wang G, Coe D, et al. Regulatory T cell migration is dependent on glucokinase-mediated glycolysis. Immunity. 2017;47:875-889.e10. 29166588 10.1016/j.immuni.2017.10.017 PMC5714502

[383] Apostolidis SA, Rodríguez-Rodríguez N, Suárez-Fueyo A, Dioufa N, Ozcan E, Crispín JC, et al. Phosphatase PP2A is requisite for the function of regulatory T cells. Nat Immunol. 2016;17:556–64. 26974206 10.1038/ni.3390 PMC4837024

[384] Alegre M-L. Treg respiration. Am J Transplant. 2019;19:969.

[385] Weinberg SE, Singer BD, Steinert EM, Martinez CA, Mehta MM, Martínez-Reyes I, et al. Mitochondrial complex III is essential for suppressive function of regulatory T cells. Nature. 2019;565:495–9. 30626970 10.1038/s41586-018-0846-z PMC6345596

[386] Li M, Yu J, Ju L, Wang Y, Jin W, Zhang R, et al. USP43 stabilizes c-Myc to promote glycolysis and metastasis in bladder cancer. Cell Death Dis. 2024;15:44. 38218970 10.1038/s41419-024-06446-7 PMC10787741

[387] Cargill KR, Stewart CA, Park EM, Ramkumar K, Gay CM, Cardnell RJ, et al. Targeting MYC-enhanced glycolysis for the treatment of small cell lung cancer. Cancer Metab. 2021;9:33. 34556188 10.1186/s40170-021-00270-9 PMC8461854

[388] Tateishi K, Iafrate AJ, Ho Q, Curry WT, Batchelor TT, Flaherty KT, et al. Myc-driven glycolysis is a therapeutic target in glioblastoma. Clin Cancer Res. 2016;22:4452–65. 27076630 10.1158/1078-0432.CCR-15-2274 PMC5010492

[389] Watson MJ, Vignali PDA, Mullett SJ, Overacre-Delgoffe AE, Peralta RM, Grebinoski S, et al. Metabolic support of tumour-infiltrating regulatory T cells by lactic acid. Nature. 2021;591:645–51. 33589820 10.1038/s41586-020-03045-2 PMC7990682

[390] Bogdanov A, Bogdanov A, Chubenko V, Volkov N, Moiseenko F, Moiseyenko V. Tumor acidity: from hallmark of cancer to target of treatment. Front Oncol. 2022;12:979154. 36106097 10.3389/fonc.2022.979154 PMC9467452

[391] Huber V, Camisaschi C, Berzi A, Ferro S, Lugini L, Triulzi T, et al. Cancer acidity: an ultimate frontier of tumor immune escape and a novel target of immunomodulation. Semin Cancer Biol. 2017;43:74–89. 28267587 10.1016/j.semcancer.2017.03.001

[392] Nief C, Chelales E, Previs R, Ramanujam N. Ethanol ablation reliably achieves an anti-metastatic response after modulating tumor acidity and regulatory T cells. Gynecol Oncol. 2021;162:S150.

[393] Rao D, Stunnenberg JA, Lacroix R, Dimitriadis P, Kaplon J, Verburg F, et al. Acidity-mediated induction of FoxP3+ regulatory T cells. Eur J Immunol. 2023;53:2250258. 10.1002/eji.202250258 36788428

[394] Shan Y, Xie T, Sun Y, Lu Z, Topatana W, Juengpanich S, et al. Lipid metabolism in tumor-infiltrating regulatory T cells: perspective to precision immunotherapy. Biomark Res. 2024;12:41. 38644503 10.1186/s40364-024-00588-8 PMC11034130

[395] Hu M, Eviston D, Hsu P, Mariño E, Chidgey A, Santner-Nanan B, et al. Decreased maternal serum acetate and impaired fetal thymic and regulatory T cell development in preeclampsia. Nat Commun. 2019;10:3031. 31292453 10.1038/s41467-019-10703-1 PMC6620275

[396] Coutzac C, Jouniaux J-M, Paci A, Schmidt J, Mallardo D, Seck A, et al. Systemic short chain fatty acids limit antitumor effect of CTLA-4 blockade in hosts with cancer. Nat Commun. 2020;11:2168. 32358520 10.1038/s41467-020-16079-x PMC7195489

[397] Schwarz A, Philippsen R, Schwarz T. Induction of regulatory T cells and correction of cytokine disbalance by short-chain fatty acids: implications for psoriasis therapy. J Invest Dermatol. 2021;141:95-104.e2. 32544478 10.1016/j.jid.2020.04.031

[398] Zhu S, Zhang J, Jiang X, Wang W, Chen YQ. Free fatty acid receptor 4 deletion attenuates colitis by modulating Treg cells via ZBED6-IL33 pathway. EBioMedicine. 2022;80:104060. 35588628 10.1016/j.ebiom.2022.104060 PMC9120243

[399] Howie D, Cobbold SP, Adams E, Ten Bokum A, Necula AS, Zhang W, et al. Foxp3 drives oxidative phosphorylation and protection from lipotoxicity. JCI Insight. 2017;2: e89160. 28194435 10.1172/jci.insight.89160 PMC5291728

[400] Miao Y, Zhang C, Yang L, Zeng X, Hu Y, Xue X, et al. The activation of PPARγ enhances Treg responses through up-regulating CD36/CPT1-mediated fatty acid oxidation and subsequent N-glycan branching of TβRII/IL-2Rα. Cell Commun Signal. 2022;20:48. 35392915 10.1186/s12964-022-00849-9 PMC8991706

[401] Miska J, Lee-Chang C, Rashidi A, Muroski ME, Chang AL, Lopez-Rosas A, et al. HIF-1α Is a metabolic switch between glycolytic-driven migration and oxidative phosphorylation-driven immunosuppression of Tregs in glioblastoma. Cell Rep. 2019;27:226-237.e4. 30943404 10.1016/j.celrep.2019.03.029 PMC6461402

[402] Yan J, Zeng Y, Guan Z, Li Z, Luo S, Niu J, et al. Inherent preference for polyunsaturated fatty acids instigates ferroptosis of Treg cells that aggravates high-fat-diet-related colitis. Cell Rep. 2024;43:114636. 39154340 10.1016/j.celrep.2024.114636

[403] Schmitz T, Freuer D, Linseisen J, Meisinger C. Associations between serum cholesterol and immunophenotypical characteristics of circulatory B cells and Tregs. J Lipid Res. 2023;64:100399. 37276940 10.1016/j.jlr.2023.100399 PMC10394386

[404] Mailer RKW, Gisterå A, Polyzos KA, Ketelhuth DFJ, Hansson GK. Hypercholesterolemia induces differentiation of regulatory T cells in the liver. Circ Res. 2017;120:1740–53. 28420668 10.1161/CIRCRESAHA.116.310054

[405] Elkins C, Sivasami P, Bae J, Li C. Cellular cholesterol homeostasis supports visceral adipose tissue (VAT) regulatory T cell (Treg) accumulation and promotes metabolic health. J Immunol. 2023;210:76.16-76.16.

[406] Shi H, Chapman NM, Wen J, Guy C, Long L, Dhungana Y, et al. Amino acids license kinase mTORC1 activity and Treg cell function via small G proteins rag and Rheb. Immunity. 2019;51:1012-1027.e7. 31668641 10.1016/j.immuni.2019.10.001 PMC6948188

[407] Yahsi B, Gunaydin G. Immunometabolism—the role of branched-chain amino acids. Front Immunol. 2022;13:886822. 35812393 10.3389/fimmu.2022.886822 PMC9259854

[408] Long Y, Tao H, Karachi A, Grippin AJ, Jin L, Chang Y, et al. Dysregulation of glutamate transport enhances Treg function that promotes VEGF blockade resistance in glioblastoma. Cancer Res. 2020;80:499–509. 31723000 10.1158/0008-5472.CAN-19-1577

[409] Lowe MM, Boothby I, Clancy S, Ahn RS, Liao W, Nguyen DN, et al. Regulatory T cells use arginase 2 to enhance their metabolic fitness in tissues. JCI Insight. 2019;4: e129756. 31852848 10.1172/jci.insight.129756 PMC6975275

[410] Zhai L, Qian J, Ladomersky E, Lauing K, Scholtens D, Lukas R, et al. IMMU-41. IDO1 increases treg recruitment independent of tryptophan metabolism in a model of glioblastoma. Neuro-Oncol. 2018;20:vi130–vi130.

[411] Gabrilovich DI, Bronte V, Chen S-H, Colombo MP, Ochoa A, Ostrand-Rosenberg S, et al. The terminology issue for myeloid-derived suppressor cells. Cancer Res. 2007;67:425. 17210725 10.1158/0008-5472.CAN-06-3037 PMC1941787

[412] Lu J, Luo Y, Rao D, Wang T, Lei Z, Chen X, et al. Myeloid-derived suppressor cells in cancer: therapeutic targets to overcome tumor immune evasion. Exp Hematol Oncol. 2024;13:39. 38609997 10.1186/s40164-024-00505-7 PMC11010322

[413] Okła K. Myeloid-derived suppressor cells (MDSCs) in ovarian cancer—looking back and forward. Cells. 2023;12:1912. 37508575 10.3390/cells12141912 PMC10377883

[414] Hu C, Pang B, Lin G, Zhen Y, Yi H. Energy metabolism manipulates the fate and function of tumour myeloid-derived suppressor cells. Br J Cancer. 2020;122:23–9. 31819182 10.1038/s41416-019-0644-x PMC6964679

[415] Gabrilovich DI. Myeloid-derived suppressor cells. Cancer Immunol Res. 2017;5:3–8. 28052991 10.1158/2326-6066.CIR-16-0297 PMC5426480

[416] Wang Y, Jia A, Bi Y, Wang Y, Liu G. Metabolic regulation of myeloid-derived suppressor cell function in cancer. Cells. 2020;9:1011. 32325683 10.3390/cells9041011 PMC7226088

[417] Deng Y, Yang J, Luo F, Qian J, Liu R, Zhang D, et al. mTOR-mediated glycolysis contributes to the enhanced suppressive function of murine tumor-infiltrating monocytic myeloid-derived suppressor cells. Cancer Immunol Immunother. 2018;67:1355–64. 29968153 10.1007/s00262-018-2177-1 PMC11028128

[418] Li J, Chen J, Zhang M, Zhang C, Wu R, Yang T, et al. The mTOR deficiency in monocytic myeloid-derived suppressor cells protects mouse cardiac allografts by inducing allograft tolerance. Front Immunol. 2021;12:661338. 33897705 10.3389/fimmu.2021.661338 PMC8062712

[419] De Veirman K, Menu E, Maes K, De Beule N, De Smedt E, Maes A, et al. Myeloid-derived suppressor cells induce multiple myeloma cell survival by activating the AMPK pathway. Cancer Lett. 2019;442:233–41. 30419344 10.1016/j.canlet.2018.11.002

[420] Jian S-L, Chen W-W, Su Y-C, Su Y-W, Chuang T-H, Hsu S-C, et al. Glycolysis regulates the expansion of myeloid-derived suppressor cells in tumor-bearing hosts through prevention of ROS-mediated apoptosis. Cell Death Dis. 2017;8:e2779–e2779. 28492541 10.1038/cddis.2017.192 PMC5520713

[421] Hayes C, Donohoe CL, Davern M, Donlon NE. The oncogenic and clinical implications of lactate induced immunosuppression in the tumour microenvironment. Cancer Lett. 2021;500:75–86. 33347908 10.1016/j.canlet.2020.12.021

[422] Zhao J-L, Ye Y-C, Gao C-C, Wang L, Ren K-X, Jiang R, et al. Notch-mediated lactate metabolism regulates MDSC development through the Hes1/MCT2/c-Jun axis. Cell Rep. 2022;38:110451. 35263597 10.1016/j.celrep.2022.110451

[423] Payen VL, Mina E, Van Hée VF, Porporato PE, Sonveaux P. Monocarboxylate transporters in cancer. Mol Metab. 2020;33:48–66. 31395464 10.1016/j.molmet.2019.07.006 PMC7056923

[424] Lemos H, Huang L, Prendergast GC, Mellor AL. Immune control by amino acid catabolism during tumorigenesis and therapy. Nat Rev Cancer. 2019;19:162–75. 30696923 10.1038/s41568-019-0106-z

[425] Ren Y, Dong X, Liu Y, Kang H, Guan L, Huang Y, et al. Rapamycin antagonizes angiogenesis and lymphangiogenesis through myeloid-derived suppressor cells in corneal transplantation. Am J Transplant. 2023;23:1359–74. 37225089 10.1016/j.ajt.2023.05.017

[426] Cimen Bozkus C, Elzey BD, Crist SA, Ellies LG, Ratliff TL. Expression of cationic amino acid transporter 2 is required for myeloid-derived suppressor cell-mediated control of T cell immunity. J Immunol. 2015;195:5237–50. 26491198 10.4049/jimmunol.1500959 PMC4655170

[427] Platten M, Nollen EAA, Röhrig UF, Fallarino F, Opitz CA. Tryptophan metabolism as a common therapeutic target in cancer, neurodegeneration and beyond. Nat Rev Drug Discov. 2019;18:379–401. 30760888 10.1038/s41573-019-0016-5

[428] Munn DH, Mellor AL. Indoleamine 2,3 dioxygenase and metabolic control of immune responses. Trends Immunol. 2013;34:137–43. 23103127 10.1016/j.it.2012.10.001 PMC3594632

[429] Opitz CA, Litzenburger UM, Sahm F, Ott M, Tritschler I, Trump S, et al. An endogenous tumour-promoting ligand of the human aryl hydrocarbon receptor. Nature. 2011;478:197–203. 21976023 10.1038/nature10491

[430] Li P, Xu W, Liu F, Zhu H, Zhang L, Ding Z, et al. The emerging roles of IDO2 in cancer and its potential as a therapeutic target. Biomed Pharmacother. 2021;137:111295. 33550042 10.1016/j.biopha.2021.111295

[431] Li A, Barsoumian HB, Schoenhals JE, Cushman TR, Caetano MS, Wang X, et al. Indoleamine 2,3-dioxygenase 1 inhibition targets anti-PD1-resistant lung tumors by blocking myeloid-derived suppressor cells. Cancer Lett. 2018;431:54–63. 29746927 10.1016/j.canlet.2018.05.005 PMC6027590

[432] Zhai L, Bell A, Ladomersky E, Lauing KL, Bollu L, Nguyen B, et al. Tumor cell IDO enhances immune suppression and decreases survival independent of tryptophan metabolism in glioblastoma. Clin Cancer Res. 2021;27:6514–28. 34479957 10.1158/1078-0432.CCR-21-1392 PMC8639612

[433] Srivastava MK, Sinha P, Clements VK, Rodriguez P, Ostrand-Rosenberg S. Myeloid-derived suppressor cells inhibit T-cell activation by depleting cystine and cysteine. Cancer Res. 2010;70:68–77. 20028852 10.1158/0008-5472.CAN-09-2587 PMC2805057

[434] Al-Khami AA, Zheng L, Del Valle L, Hossain F, Wyczechowska D, Zabaleta J, et al. Exogenous lipid uptake induces metabolic and functional reprogramming of tumor-associated myeloid-derived suppressor cells. OncoImmunology. 2017;6: e1344804. 29123954 10.1080/2162402X.2017.1344804 PMC5665069

[435] Wang Q, Zhang X, Li C, Xiong M, Bai W, Sun S, et al. Intracellular lipid accumulation drives the differentiation of decidual polymorphonuclear myeloid-derived suppressor cells via arachidonic acid metabolism. Front Immunol. 2022;13:868669. 35664000 10.3389/fimmu.2022.868669 PMC9159278

[436] Song C, Ji Y, Wang W, Tao N. Ginger polysaccharide promotes myeloid-derived suppressor cell apoptosis by regulating lipid metabolism. Phytother Res. 2023;37:2894–901. 36806265 10.1002/ptr.7784

[437] Zhang Y, You P, Liu R, Lu Y, Li J, Lei Y, et al. Artificial intelligence in clinical trials of lung cancer: current and future prospects. Intell Oncol. 2025;1:34–51.

[438] Cioce M, Pulito C, Strano S, Blandino G, Fazio VM. Metformin: metabolic rewiring faces tumor heterogeneity. Cells. 2020;9:2439. 33182253 10.3390/cells9112439 PMC7695274

[439] Jia D, Lu M, Jung KH, Park JH, Yu L, Onuchic JN, et al. Elucidating cancer metabolic plasticity by coupling gene regulation with metabolic pathways. Proc Natl Acad Sci. 2019;116:3909–18. 30733294 10.1073/pnas.1816391116 PMC6397570

[440] Yu Z, Wang Y, Wang B, Zhai J. Metformin affects paclitaxel sensitivity of ovarian cancer cells through autophagy mediated by long noncoding RNASNHG7/miR-3127-5p Axis. Cancer Biother Radiopharm. 2022;37:792–801. 32522016 10.1089/cbr.2019.3390

[441] Dixon-Zegeye M, Shaw R, Collins L, Perez-Smith K, Ooms A, Qiao M, et al. Cancer precision-prevention trial of metformin in adults with Li fraumeni syndrome (MILI) undergoing yearly MRI surveillance: a randomised controlled trial protocol. Trials. 2024;25:103. 38308321 10.1186/s13063-024-07929-w PMC10837926

[442] Metts JL, Trucco M, Weiser DA, Thompson P, Sandler E, Smith T, et al. A phase I trial of metformin in combination with vincristine, irinotecan, and temozolomide in children with relapsed or refractory solid and central nervous system tumors: a report from the national pediatric cancer foundation. Cancer Med. 2023;12:4270–81. 36151773 10.1002/cam4.5297 PMC9972017

[443] Sadeghi N, Abbruzzese JL, Yeung S-CJ, Hassan M, Li D. Metformin use is associated with better survival of diabetic patients with pancreatic cancer. Clin Cancer Res. 2012;18:2905–12. 22465831 10.1158/1078-0432.CCR-11-2994 PMC3381457

[444] Delgir S, Bastami M, Ilkhani K, Safi A, Seif F, Alivand MR. The pathways related to glutamine metabolism, glutamine inhibitors and their implication for improving the efficiency of chemotherapy in triple-negative breast cancer. Mutat Res Mutat Res. 2021;787:108366. 10.1016/j.mrrev.2021.108366 34083056

[445] Hong J, Shen Y-A, Hsu C-Y, Huang P, Tomaszewski A, Gabrielson E, et al. Targeting glutamine metabolism enhances responses to platinum-based chemotherapy in triple-negative breast cancers (TNBC). Genes Dis. 2022;9:1408–11. 36157486 10.1016/j.gendis.2022.02.009 PMC9485268

[446] Zhao Y, Feng X, Chen Y, Selfridge JE, Gorityala S, Du Z, et al. 5-fluorouracil enhances the antitumor activity of the glutaminase inhibitor CB-839 against *PIK3CA* -mutant colorectal cancers. Cancer Res. 2020;80:4815–27. 32907836 10.1158/0008-5472.CAN-20-0600 PMC7642187

[447] Yang W-H, Qiu Y, Stamatatos O, Janowitz T, Lukey MJ. Enhancing the efficacy of glutamine metabolism inhibitors in cancer therapy. Trends Cancer. 2021;7:790–804. 34020912 10.1016/j.trecan.2021.04.003 PMC9064286

[448] Timofeeva N, Ayres ML, Baran N, Santiago-O’Farrill JM, Bildik G, Lu Z, et al. Preclinical investigations of the efficacy of the glutaminase inhibitor CB-839 alone and in combinations in chronic lymphocytic leukemia. Front Oncol. 2023;13:1161254. 37228498 10.3389/fonc.2023.1161254 PMC10203524

[449] DiNardo CD, Verma D, Baran N, Bhagat TD, Skwarska A, Lodi A, et al. Glutaminase inhibition in combination with azacytidine in myelodysplastic syndromes: a phase 1b/2 clinical trial and correlative analyses. Nat Cancer. 2024;5:1515–33. 39300320 10.1038/s43018-024-00811-3 PMC13318259

[450] Zhang Y, Sun M, Zhao H, Wang Z, Shi Y, Dong J, et al. Neuroprotective effects and therapeutic potential of dichloroacetate: targeting metabolic disorders in nervous system diseases. Int J Nanomed. 2023;18:7559–81. 10.2147/IJN.S439728 PMC10725694 38106446

[451] Gray LR, Tompkins SC, Taylor EB. Regulation of pyruvate metabolism and human disease. Cell Mol Life Sci CMLS. 2014;71:2577–604. 24363178 10.1007/s00018-013-1539-2 PMC4059968

[452] Chen I-C, Awasthi D, Hsu C-L, Song M, Chae C-S, Dannenberg AJ, et al. High-fat diet-induced obesity alters dendritic cell homeostasis by enhancing mitochondrial fatty acid oxidation. J Immunol. 2022;209:69–76. 35697385 10.4049/jimmunol.2100567 PMC9247030

[453] Adeshakin AO, Liu W, Adeshakin FO, Afolabi LO, Zhang M, Zhang G, et al. Regulation of ROS in myeloid-derived suppressor cells through targeting fatty acid transport protein 2 enhanced anti-PD-L1 tumor immunotherapy. Cell Immunol. 2021;362:104286. 33524739 10.1016/j.cellimm.2021.104286

[454] Tan VP, Miyamoto S. HK2/hexokinase-II integrates glycolysis and autophagy to confer cellular protection. Autophagy. 2015;11:963–4. 26075878 10.1080/15548627.2015.1042195 PMC4502787

[455] Feng J, Li J, Wu L, Yu Q, Ji J, Wu J, et al. Emerging roles and the regulation of aerobic glycolysis in hepatocellular carcinoma. J Exp Clin Cancer Res CR. 2020;39:126. 32631382 10.1186/s13046-020-01629-4 PMC7336654

[456] Bost F, Decoux-Poullot A-G, Tanti JF, Clavel S. Energy disruptors: rising stars in anticancer therapy? Oncogenesis. 2016;5:e188–e188. 26779810 10.1038/oncsis.2015.46 PMC4728676

[457] Jin T, Mehrens H, Wang P, Kim S-G. Glucose metabolism-weighted imaging with chemical exchange-sensitive MRI of 2-deoxyglucose (2DG) in brain: sensitivity and biological sources. Neuroimage. 2016;143:82–90. 27570111 10.1016/j.neuroimage.2016.08.040 PMC5124509

[458] Zhou H, Luby-Phelps K, Mickey BE, Habib AA, Mason RP, Zhao D. Dynamic near-infrared optical imaging of 2-deoxyglucose uptake by intracranial glioma of athymic mice. PLoS ONE. 2009;4: e8051. 19956682 10.1371/journal.pone.0008051 PMC2778127

[459] Wang Q, Liang B, Shirwany NA, Zou M-H. 2-deoxy-D-glucose treatment of endothelial cells induces autophagy by reactive oxygen species-mediated activation of the AMP-activated protein kinase. PLoS ONE. 2011;6: e17234. 21386904 10.1371/journal.pone.0017234 PMC3046135

[460] Emmett L, Subramaniam S, Joshua AM, Crumbaker M, Martin A, Zhang AY, et al. ENZA-p trial protocol: a randomized phase II trial using prostate-specific membrane antigen as a therapeutic target and prognostic indicator in men with metastatic castration-resistant prostate cancer treated with enzalutamide (ANZUP 1901). BJU Int. 2021;128:642–51. 34028967 10.1111/bju.15491

[461] Mena E, Shih J, Chung J-Y, Jones J, Rabiee A, Monge C, et al. Functional imaging of liver cancer (FLIC): study protocol of a phase 2 trial of 18F-DCFPyL PET/CT imaging for patients with hepatocellular carcinoma. PLoS ONE. 2022;17: e0277407. 36367894 10.1371/journal.pone.0277407 PMC9651549

[462] Karbhari A, Mosessian S, Trivedi KH, Valla F, Jacobson M, Truty MJ, et al. Gallium-68-labeled fibroblast activation protein inhibitor-46 PET in patients with resectable or borderline resectable pancreatic ductal adenocarcinoma: a phase 2, multicenter, single arm, open label non-randomized study protocol. PLoS ONE. 2023;18: e0294564. 38011131 10.1371/journal.pone.0294564 PMC10681241

[463] Simons AL, Ahmad IM, Mattson DM, Dornfeld KJ, Spitz DR. 2-deoxy- d -glucose combined with cisplatin enhances cytotoxicity via metabolic oxidative stress in human head and neck cancer cells. Cancer Res. 2007;67:3364–70. 17409446 10.1158/0008-5472.CAN-06-3717 PMC3852417

[464] Ma S, Jia R, Li D, Shen B. Targeting cellular metabolism chemosensitizes the doxorubicin-resistant human breast adenocarcinoma cells. BioMed Res Int. 2015;2015:1–8. 10.1155/2015/453986 PMC4628972 26558272

[465] Kim B, Sun R, Oh W, Kim AMJ, Schwarz JR, Lim S. Saccharide analog, 2-deoxy- d -glucose enhances 4–1BB-mediated antitumor immunity via PD-L1 deglycosylation. Mol Carcinog. 2020;59:691–700. 32115801 10.1002/mc.23170

[466] Stein M, Lin H, Jeyamohan C, Dvorzhinski D, Gounder M, Bray K, et al. Targeting tumor metabolism with 2-deoxyglucose in patients with castrate-resistant prostate cancer and advanced malignancies. Prostate. 2010;70:1388–94. 20687211 10.1002/pros.21172 PMC4142700

[467] Menendez JA, Lupu R. Fatty acid synthase and the lipogenic phenotype in cancer pathogenesis. Nat Rev Cancer. 2007;7:763–77. 17882277 10.1038/nrc2222

[468] Sardesai SD, Thomas A, Gallagher C, Lynce F, Ottaviano YL, Ballinger TJ, et al. Inhibiting fatty acid synthase with omeprazole to improve efficacy of neoadjuvant chemotherapy in patients with operable TNBC. Clin Cancer Res. 2021;27:5810–7. 34400413 10.1158/1078-0432.CCR-21-0493

[469] Kelly W, Diaz Duque AE, Michalek J, Konkel B, Caflisch L, Chen Y, et al. Phase II investigation of TVB-2640 (denifanstat) with bevacizumab in patients with first relapse high-grade astrocytoma. Clin Cancer Res. 2023;29:2419–25. 37093199 10.1158/1078-0432.CCR-22-2807 PMC10320469

[470] Kang Y-K, Chin K, Chung HC, Kadowaki S, Oh SC, Nakayama N, et al. S-1 plus leucovorin and oxaliplatin versus S-1 plus cisplatin as first-line therapy in patients with advanced gastric cancer (SOLAR): a randomised, open-label, phase 3 trial. Lancet Oncol. 2020;21:1045–56. 32682457 10.1016/S1470-2045(20)30315-6

[471] Bonora M, Patergnani S, Rimessi A, De Marchi E, Suski JM, Bononi A, et al. ATP synthesis and storage. Purinergic Signal. 2012;8:343–57. 22528680 10.1007/s11302-012-9305-8 PMC3360099

[472] Greene J, Segaran A, Lord S. Targeting OXPHOS and the electron transport chain in cancer; molecular and therapeutic implications. Semin Cancer Biol. 2022;86:851–9. 35122973 10.1016/j.semcancer.2022.02.002

[473] Yap TA, Daver N, Mahendra M, Zhang J, Kamiya-Matsuoka C, Meric-Bernstam F, et al. Complex I inhibitor of oxidative phosphorylation in advanced solid tumors and acute myeloid leukemia: phase I trials. Nat Med. 2023;29:115–26. 36658425 10.1038/s41591-022-02103-8 PMC11975418

[474] Ye D, Guan K-L, Xiong Y. Metabolism, activity, and targeting of D- and L-2-hydroxyglutarates. Trends Cancer. 2018;4:151–65. 29458964 10.1016/j.trecan.2017.12.005 PMC5884165

[475] Zhang C, Moore LM, Li X, Yung WKA, Zhang W. IDH1/2 mutations target a key hallmark of cancer by deregulating cellular metabolism in glioma. Neuro-Oncol. 2013;15:1114–26. 23877318 10.1093/neuonc/not087 PMC3748922

[476] Shallis RM, Podoltsev NA. Maintenance therapy for acute myeloid leukemia: Sustaining the pursuit for sustained remission. Curr Opin Hematol. 2021;28:110–21. 33394722 10.1097/MOH.0000000000000637

[477] Li Q, Zhou Z-W, Lu J, Luo H, Wang S-N, Peng Y, et al. PD-L1P146R is prognostic and a negative predictor of response to immunotherapy in gastric cancer. Mol Ther J Am Soc Gene Ther. 2022;30:621–31. 10.1016/j.ymthe.2021.09.013 PMC8821936 34547468

[478] Szeto GL, Finley SD. Integrative approaches to cancer immunotherapy. Trends Cancer. 2019;5:400–10. 31311655 10.1016/j.trecan.2019.05.010 PMC7467854

[479] Chen J, Cao X, Li B, Zhao Z, Chen S, Lai SWT, et al. warburg effect is a cancer immune evasion mechanism against macrophage immunosurveillance. Front Immunol. 2021;11:621757. 33603751 10.3389/fimmu.2020.621757 PMC7884830

[480] Li X, Wenes M, Romero P, Huang SC-C, Fendt S-M, Ho P-C. Navigating metabolic pathways to enhance antitumour immunity and immunotherapy. Nat Rev Clin Oncol. 2019;16:425–41. 30914826 10.1038/s41571-019-0203-7

[481] Carlino MS, Larkin J, Long GV. Immune checkpoint inhibitors in melanoma. Lancet Lond Engl. 2021;398:1002–14. 10.1016/S0140-6736(21)01206-X 34509219

[482] Lontos K, Wang Y, Joshi SK, Frisch AT, Watson MJ, Kumar A, et al. Metabolic reprogramming via an engineered PGC-1α improves human chimeric antigen receptor T-cell therapy against solid tumors. J Immunother Cancer. 2023;11: e006522. 36914208 10.1136/jitc-2022-006522 PMC10016249

[483] Ando M, Ito M, Srirat T, Kondo T, Yoshimura A. Memory T cell, exhaustion, and tumor immunity. Immunol Med. 2020;43:1–9. 31822213 10.1080/25785826.2019.1698261

[484] Specenier P. Nivolumab in melanoma. Expert Rev Anticancer Ther. 2016;16:1247–61. 27776441 10.1080/14737140.2016.1249856

[485] Sloan AE, Winter K, Gilbert MR, Aldape K, Choi S, Wen PY, et al. NRG-BN002: phase I study of ipilimumab, nivolumab, and the combination in patients with newly diagnosed glioblastoma. Neuro-Oncol. 2024;26:1628–37. 38874333 10.1093/neuonc/noae058 PMC11376446

[486] Zibelman M, MacFarlane AW, Costello K, McGowan T, O’Neill J, Kokate R, et al. A phase 1 study of nivolumab in combination with interferon-gamma for patients with advanced solid tumors. Nat Commun. 2023;14:4513. 37500647 10.1038/s41467-023-40028-z PMC10374608

[487] Botsen D, Chabaud S, Perrier H, Ammarguellat H, Jestin-Le-Tallec V, Olesinski J, et al. Trifluridine/tipiracil + oxaliplatin ± nivolumab vs FOLFOX ± nivolumab in HER2 negative advanced oesogastric adenocarcinoma: the PRODIGE73-UCGI40-LOGICAN trial. Dig Liver Dis. 2024;56:1281–7. 38762353 10.1016/j.dld.2024.04.032

[488] Postow MA, Chesney J, Pavlick AC, Robert C, Grossmann K, McDermott D, et al. Nivolumab and ipilimumab versus ipilimumab in untreated melanoma. N Engl J Med. 2015;372:2006–17. 25891304 10.1056/NEJMoa1414428 PMC5744258

[489] Specenier P. Ipilimumab in melanoma. Expert Rev Anticancer Ther. 2016;16:811–26. 27403706 10.1080/14737140.2016.1211936

[490] Van Coillie S, Wiernicki B, Xu J. Molecular and cellular functions of CTLA-4. Adv Exp Med Biol. 2020;1248:7–32. 32185705 10.1007/978-981-15-3266-5_2

[491] Park JS, Kim J, Jeon J, Lee J, Jang WS, Lee SH, et al. The role of cytoreductive nephrectomy in metastatic renal cell carcinoma in immune-oncology era (SEVURO-CN): study protocol for a multi-center, prospective, randomized trial. Trials. 2024;25:447. 38961439 10.1186/s13063-024-08234-2 PMC11223430

[492] Zhou Y, Liu Z, Yu A, Zhao G, Chen B. Immune checkpoint inhibitor combined with antiangiogenic agent synergistically improving the treatment efficacy for solid tumors. ImmunoTargets Ther. 2024;13:813–29. 39763508 10.2147/ITT.S494670 PMC11700879

[493] Koshkin VS, Danchaivijitr P, Bae WK, Semenov A, Ozyilkan O, Su Y-L, et al. Pembrolizumab retreatment in patients with advanced or metastatic urothelial carcinoma who responded to first-course pembrolizumab-based therapy. Eur Urol. 2025;87:390–5. 39709248 10.1016/j.eururo.2024.11.012

[494] Zugman M, Nguyen CB. Pembrolizumab retreatment: lessons from a selected group of patients with urothelial carcinoma. Eur Urol. 2025;S0302–2838(25):6–5. 10.1016/j.eururo.2025.01.002 39827020

[495] Zsiros E, Lynam S, Attwood KM, Wang C, Chilakapati S, Gomez EC, et al. Efficacy and safety of pembrolizumab in combination with bevacizumab and oral metronomic cyclophosphamide in the treatment of recurrent ovarian cancer: a phase 2 nonrandomized clinical trial. JAMA Oncol. 2021;7:78–85. 33211063 10.1001/jamaoncol.2020.5945 PMC7677872

[496] Yang C, Blum NT, Lin J, Qu J, Huang P. Biomaterial scaffold-based local drug delivery systems for cancer immunotherapy. Sci Bull. 2020;65:1489–504. 10.1016/j.scib.2020.04.012 36747406

[497] Zhu X, Li S. Nanomaterials in tumor immunotherapy: new strategies and challenges. Mol Cancer. 2023;22:94. 37312116 10.1186/s12943-023-01797-9 PMC10262535

[498] Zhu K, Wang L, Xiao Y, Zhang X, You G, Chen Y, et al. Nanomaterial-related hemoglobin-based oxygen carriers, with emphasis on liposome and nano-capsules, for biomedical applications: current status and future perspectives. J Nanobiotechnol. 2024;22:336. 10.1186/s12951-024-02606-1 PMC11180412 38880905

[499] Chen Y, Huang Y, Li Q, Luo Z, Zhang Z, Huang H, et al. Targeting Xkr8 via nanoparticle-mediated in situ co-delivery of siRNA and chemotherapy drugs for cancer immunochemotherapy. Nat Nanotechnol. 2023;18:193–204. 36424448 10.1038/s41565-022-01266-2 PMC9974593

[500] Xu J, Zhao W, Sun J, Huang Y, Wang P, Venkataramanan R, et al. Novel glucosylceramide synthase inhibitor based prodrug copolymer micelles for delivery of anticancer agents. J Controlled Release. 2018;288:212–26. 10.1016/j.jconrel.2018.09.011 PMC6177216 30223045

[501] Terse PS, Joshi PS, Bordelon NR, Brys AM, Patton KM, Arndt TP, et al. 2-deoxy-d-glucose (2-DG)-induced cardiac toxicity in rat: NT-proBNP and BNP as potential early cardiac safety biomarkers. Int J Toxicol. 2016;35:284–93. 26838190 10.1177/1091581815624397 PMC4864115

[502] Futamura M, Ishihara K, Nagao Y, Ogiso A, Niwa Y, Nakada T, et al. Neoadjuvant chemotherapy using nanoparticle albumin-bound paclitaxel plus trastuzumab and pertuzumab followed by epirubicin and cyclophosphamide for operable HER2-positive primary breast cancer: a multicenter phase II clinical trial (PerSeUS-BC04). Breast Cancer Tokyo Jpn. 2023;30:293–301. 10.1007/s12282-022-01425-2 PMC9950177 36609911

[503] Bennett S, Verry C, Kaza E, Miao X, Dufort S, Boux F, et al. Quantifying gadolinium-based nanoparticle uptake distributions in brain metastases via magnetic resonance imaging. Sci Rep. 2024;14:11959. 38796495 10.1038/s41598-024-62389-1 PMC11128019

[504] Zuo Y, Lu W, Xia Y, Meng J, Zhou Y, Xiao Y, et al. Glucometer readout for portable detection of heterogeneous circulating tumor cells in lung cancer captured on a dual aptamer functionalized wrinkled cellulose hydrogel interface. ACS Sens. 2023;8:187–96. 36562728 10.1021/acssensors.2c02029

[505] Zhu Y, Hao Q, Zhu H, Zhao R, Feng L, He S, et al. Thermoelectric nanoheterojunction-mediated multiple energy conversion for enhanced cancer therapy. ACS Nano. 2024;18:34257–71. 39630424 10.1021/acsnano.4c12261

[506] Suvarna S, Das U, Kc S, Mishra S, Sudarshan M, Saha KD, et al. Synthesis of a novel glucose capped gold nanoparticle as a better theranostic candidate. PLoS ONE. 2017;12: e0178202. 28582426 10.1371/journal.pone.0178202 PMC5459428

[507] Farag AF, Hassabou NF. CD24-gold nanocomposite as promising and sensitive biomarker for cancer stem cells in salivary gland tumors. Nanomed Nanotechnol Biol Med. 2022;46:102598. 10.1016/j.nano.2022.102598 36089234

[508] Zmerli I, Ibrahim N, Cressey P, Denis S, Makky A. Design and synthesis of new PEGylated polydopamine-based nanoconstructs bearing ROS-responsive linkers and a photosensitizer for bimodal photothermal and photodynamic therapies against cancer. Mol Pharm. 2021;18:3623–37. 34431682 10.1021/acs.molpharmaceut.1c00597

[509] Zhu Q, Ademuyiwa FO, Young C, Appleton C, Covington MF, Ma C, et al. Early assessment window for predicting breast cancer neoadjuvant therapy using biomarkers, ultrasound, and diffuse optical tomography. Breast Cancer Res Treat. 2021;188:615–30. 33970392 10.1007/s10549-021-06239-y PMC8487763

